# Design, synthesis, molecular docking, and molecular dynamic studies of novel quinazoline derivatives as phosphodiesterase 7 inhibitors

**DOI:** 10.3389/fphar.2024.1389076

**Published:** 2024-04-22

**Authors:** Afaf A. El-Malah, Magdy M. Gineinah, Maan T. Khayat, Anfal S. Aljahdali, Marwa M. Safar, Hadeel A. Almazmumi, Roaa M. Khinkar

**Affiliations:** ^1^ Department of Pharmaceutical Chemistry, College of Pharmacy, King Abdulaziz University, Jeddah, Saudi Arabia; ^2^ Department of Pharmacology and Toxicology, Faculty of Pharmacy, Cairo University, Giza, Egypt; ^3^ Department of Pharmacology and Biochemistry, Faculty of Pharmacy, The British University in Egypt, Cairo, Egypt; ^4^ Department of Pharmacy Practice, College of Pharmacy, King Abdulaziz University, Jeddah, Saudi Arabia

**Keywords:** phosphodiesterase 7 inhibitors, hydrazinoquinazolines, triazoloquinazolines anti-inflammatory, molecular docking, molecular dynamic, *in vitro* assay

## Abstract

**Introduction:** Phosphodiesterase 7 (PDE7) is a high-affinity cyclic AMP (cAMP)-specific PDE that is expressed in immune and proinflammatory cells. In this work, we explore the possibility that selective small molecule inhibitors of this enzyme family could provide a novel approach to alleviate the inflammation that is associated with many inflammatory diseases.

**Methods:** A series of novel substituted 4-hydrazinoquinazoline derivatives and fused triazoloquinazolines were designed, synthesized, and evaluated *in vitro* for their PDE7A inhibition activities, in comparison with Theophylline, a non-selective PDE inhibitor, and BRL50481, a selective PDE7A inhibitor. This series of novel quinazoline derivatives were synthesized via multi-step reactions. The reaction sequence began with selective monohydrazinolysis of compounds 2a,b to give 3a,b. Schiff bases 4a-h were synthesized by the reaction of the quinazolylhydrazines 3a,b with various substituted aromatic aldehydes. The reaction of 4a-h with bromine in acetic acid, in turn, gave fused triazoloquinazolines 5a-h. These compounds were characterized by satisfied spectrum analyses mainly including ^1^HNMR, ^13^CNMR, and MS together with elemental analyses.

**Results and discussion:** The results of *in vitro* PDE7A inhibition activity clearly indicated that compounds 4b, 4g, 5c, and 5f exhibited good potency. Molecular docking and molecular dynamic simulation studies further supported our findings and provided the basis of interaction in terms of conventional hydrogen bonds and *π*-*π* stacking patterns. The present results lay the groundwork for developing lead compounds with improved phosphodiesterase seven inhibitory activities.

## Introduction

Anti-inflammatory agents are a symptomatic therapy that inhibits the whole or any portion of an acute or chronic inflammation reaction. There are different categories of anti-inflammatory agents: nonsteroidal acidic compounds, nonsteroidal nonacidic compounds, and steroids. Although nonsteroidal anti-inflammatory drugs (NSAIDs) and corticosteroids are enormous medications for inflammation, the long-term side effects are very dangerous (Hart and Huskisson, 1984; El-Malah et al., 2022; Hassan et al., 2022; Sohail et al., 2023). As a consequence, scientists search for new anti-inflammatory drugs. Diseases characterized by chronic inflammation are often associated with increased levels of phosphodiesterases (PDEs) (Paterniti et al., 2011).

Phosphodiesterases are a large group of phosphohydrolase enzymes that metabolize adenosine and guanosine 3′, 5′- cyclic monophosphates (c-AMP and c-GMP) to inactive 5′-monophosphates. PDEs consisting of 11 families (PDE 1-11) distribute differently in cells and tissues (Kumar et al., 2013). Cytokines are signaling molecules that play a key role in the immune response and can contribute to the development of inflammation. By increasing the levels of c-AMP and c-GMP, PDE inhibitors can inhibit the activation of transcription factors such as nuclear factor Kappa B (NF.KB) and activator protein 1 (AP-1), which regulate the expression of pro-inflammatory cytokines such as interleukin- 1 (IL-1), interleukin-6 (IL-6), and tumor necrosis factor-alpha (TNF-α) (Maurice et al., 2014).

Another mechanism by which PDE inhibitors can exert anti-inflammatory effects is by modulating the activity of immune cells, such as macrophages and T lymphocytes. PDE inhibitors can promote the differentiation of regulatory T cells (T regs), which are immune cells that suppress the activity of other immune hemostasis. Overall, the anti-inflammatory effects of PDE inhibitors are likely to be due to a combination of these different Pathways (Zhang et al., 2008). So, targeting PDEs has been confirmed to be a fruitful and productive strategy for inflammation control. In terms of chemical structure, quinazoline derivatives are found to be potent drugs that have a wide pharmaceutical application and a range of biological activities such as antitumor (Deli et al., 1984; Giorgi-Renault et al., 1991; Jackman et al., 1991; Gangjee et al., 1995; Jackman et al., 1995; Hamel et al., 1996; Jantová et al., 1997; Griffin et al., 1998; Raffa et al., 1999; Rosowsky et al., 1999; Bavetsias et al., 2000), antituberculosis (Devi and Kachroo, 2014), anticonvulsant (Buchanan and Sable, 1972) and anti-inflammatory (Ozaki et al., 1985; Gineinah et al., 2000).

**FIGURE 1:**
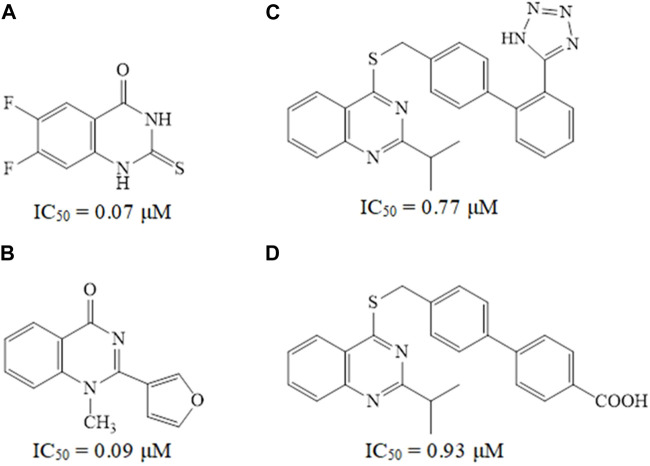
Some reported quinazoline derivatives as PDE7 inhibitors.

Quinazoline derivatives A and B have been identified as inhibitors of PDE7 (Elfeky et al., 2020), moreover, sulfarylquinazolines C and D have been reported to exhibit inhibition of PDE7 (Sánchez et al., 2013). Additionally, it has been observed that all these quinazoline derivatives possess significant anti-inflammatory activity (Figure 1). It is worth knowing that PDE7 is a cAMP-specific phosphodiesterase enzyme (Keravis and Lugnier, 2012; Amin, et al., 2021). PDE7 consists of two members, one of them is PDE7A, which is widely expressed in skeletal muscle and T-lymphocytes. It plays a significant role in regulating the immune system and inflammation (Menniti et al., 2006; Brandon and Rotella, 2007). Inhibitors of PDE7A are promising targets showing anti-inflammatory activity with minimal clinical side effects (Morales-Garcia et al., 2011).

Recently, a series of triazole derivatives were designed and synthesized as PDE7 inhibitors and evaluated further for anti-inflammatory activity (Grewal et al., 2017). So, focusing on PDE7A might offer a new approach to boost cAMP levels while reducing the risk of adverse drug reactions associated with previous types of phosphodiesterase inhibitors. Based on the aforementioned literature survey, the objective of this study is to create and produce new quinazoline derivatives with varied structural characteristics that exhibit PDE7A inhibition activity for use as anti-inflammatory agents. The design of these novel derivatives involves introducing various heterocyclic rings, such as furan, pyridine, indole, and quinoline, to the quinazoline nucleus, along with incorporating a triazole moiety into the quinazoline core. Additionally, a chloro group will be integrated into the benzene ring of some novel quinazoline derivatives. Incorporating heterocycles and/or a chloro group onto the quinazoline core could potentially enhance stacking interactions and improve hydrophobic interactions at the enzyme binding site. In the present contribution, biological screening was accomplished for all synthesized quinazoline derivatives as PDE7A inhibitors. Additionally, we studied the molecular docking and molecular dynamic simulations of some of our novel synthesized quinazolines.

## Materials and methods

### Chemistry

Sigma-Aldrich Company is the only supplier of all the used chemicals. Melting points were obtained on a Griffin apparatus and were uncorrected. Microanalyses for C, H, and N were carried out at the Regional Center for Mycology and Biotechnology, Faculty of Pharmacy, Al-Azhar University, and values were accepted within a range of ±0.4% of theoretically calculated percentages. In addition, Mass spectra were recorded on Jeol-Gas chromatography-mass spectrometry, JMS-Q1500GC (Tokyo, Japan) controlled with Escrime software.^1^HNMR spectra were carried out on Bruker 400 MHz (Bruker Corp., Billerica, MA, USA) spectrophotometer, Faculty of Pharmacy, Cairo University, Cairo, Egypt. Chemical shifts were recorded in ppm on *δ* scale, coupling constants (*J*) were given in Hz, and peak multiplicities are designed as follows: s, singlet; d, doublet; dd, doublet of doublet; t, triplet; m, multiplet. ^13^CNMR spectra were carried out on Bruker 100 MHz spectrophotometer, Faculty of Pharmacy, Cairo University, Cairo, Egypt. Progress of the reactions was monitored by TLC using precoated aluminium sheet silica gel MERCK 60F 254 and was visualized by UV lamp. Compounds **1a,b** were prepared according to the reported procedures (Russo et al., 1966). In addition, compounds **2a,b** were synthesized and reported (Curd et al., 1947; El-kerdawy et al., 1990).

#### General procedure of preparation of compounds (3a,b)

Anhydrous hydrazine (0.6 g, 19 mmol) was titrated to an ethanolic solution (20 mL) of the dichloroquinazoline derivative (2.5 mmol) at 0°C. The mixture was stirred for 30 min at the same temperature. The formed solid was filtered, washed with ethanol, and recrystallized from the EtOH/CH_2_Cl_2_.

#### General procedure of preparation of compounds (4a-h)

A mixture of the specific quinazolylhydrazine derivative **3a** or **3b** (1.54 mol), the respective substituted aldehyde (1.62 mol), and absolute ethanol (25 mL) were all stirred at room temperature for 30 min. The resulting precipitate was collected by filtration and the solid was recrystallized from acetonitrile.

#### 2-Chloro-4-[2-(5-hydroxymethyl-2-furylmethylene)hydrazino]quinazoline (4a)

Yield, 39%; mp < 280°C; ^1^HNMR (DMSO-d6) ppm δ: 11.21 (d, 1H, NH, D_2_O exchangeable), 7.89-7.87 (m, 2H, Ar-H), 7.64-7.60 (m, 2H, Ar-H), 7.17-7.14 (m, 3H, Ar-H, ArCH = N), 6.50 (s, 1H, OH, D_2_O exchangeable), 4.48 (s, 2H, CH_2_); ^13^CNMR (DMSO-d6) ppm δ: 163.3, 159.8, 150.7, 148.8, 141.3, 135.4, 127.4, 122.7, 118.7, 115.7, 114.7, 110.0, 56.2; MS m/z (%): 302 (M^+^, 24), 256 (100). Calcd. For C_14_H_11_ClN_4_O_2_ (302.72): C, 55.54; H, 3.66; N, 18.51. Found: C, 55.70; H, 3.84; N, 18.68.

#### 2-Chloro-4-[2-(3-pyridylmethylene)hydrazino]quinazoline (4b)

Yield, 35%; mp < 280°C; ^1^HNMR (DMSO-d6) ppm δ: 11.21 (d, 1H, NH, D_2_O exchangeable), 9.01 (s, 1H, Ar-H), 8.77 (s, 1H, Ar-H), 8.69 (d, *J* = 4 Hz, 1H, Ar-H), 8.26 (d, *J* = 8 Hz, 1H, Ar-H), 7.88 (dd, *J* = 8, 4 Hz, 1H, Ar-H), 7.64-7.62 (m, 1H, Ar-H), 7.60-7.52 (m, 1H, Ar-H), 7.18 (d, 1H, ArCH = N), 7.16-7.14 (m, 1H, Ar-H); ^13^CNMR (DMSO-d6) ppm δ: 163.3, 160.1, 152.4, 150.7, 150.3, 141.3, 135.4, 129.8, 127.4, 124.5, 122.7, 115.7, 114.7; MS m/z (%): 283 (M^+^, 13), 89 (100). Calcd. For C_14_H_10_ClN_5_ (283.72): C, 59.26; H, 3.55; N, 24.68. Found: C, 59.46; H, 3.68; N, 24.85.

#### 4-[2-(5-bromo-1H-3-indolylmethylene)hydrazino]-2-chloroquinazoline (4c)

Yield, 75%; mp 238-240°C; ^1^HNMR (DMSO-d6) ppm δ: 11.91 (s, 1H, NH indole, D_2_O exchangeable), 11.21 (d, 1H, NH, D_2_O exchangeable),8.92 (s, 1H, Ar-H), 8.50 (d, *J* = 4 Hz, 1H, Ar-H), 7.99 (d, *J* = 4 Hz, 1H, Ar-H), 7.90-7.88 (m, 1H, Ar-H), 7.65-7.61 (m, 1H, Ar-H), 7.46 (d, 1H, ArCH = N), 7.36 (d, *J* = 2 Hz, 1H, Ar-H), 7.34 (d, *J* = 2 Hz, 1H, Ar-H), 7.19-7.15 (m, 1H, Ar-H); ^13^CNMR (DMSO-d6) ppm δ: 163.0, 155.7, 150.7, 141.3, 136.4, 135.4, 133.8, 127.4, 126.8, 125.6, 124.6, 122.7, 115.7, 114.7, 114.5, 113.7, 112.0; Calcd. For C_17_H_11_BrClN_5_ (400.66): C, 50.95; H, 2.76; N, 17.48. Found: C, 51.23; H, 3.10; N, 17.65.

#### 2-Chloro-4-[2-(4-quinolinylmethylene)hydrazino]quinazoline (4d)

Yield, 54%; mp 205-207°C; ^1^HNMR (DMSO-d6) ppm δ: 11.21 (d, 1H, NH, D_2_O exchangeable), 9.42 (s, 1H, Ar-H), 9.05 (d, *J* = 4 Hz, 1H, Ar-H), 9.02 (d, *J* = 8 Hz, 1H, Ar-H), 8.16 (d, *J* = 8 Hz, 1H, Ar-H), 8.03 (d, *J* = 4 Hz, 1H, Ar-H), 7.90-7.86 (m, 2H, Ar-H), 7.65-7.61 (m, 2H, Ar-H), 7.19-7.16 (m, 2H, Ar-H, ArCH = N); ^13^CNMR (DMSO-d6) ppm δ: 163.3, 160.2, 150.9, 150.7, 148.9, 141.3, 136.9, 135.4, 133.2, 130.3, 128.3,127.4, 125.5, 125.3, 122.7, 122.3, 115.7, 114.7; MS m/z (%): 333 (M^+^, 23), 151 (100). Calcd. For C_18_H_12_ClN_5_ (333.77): C, 64.76; H, 3.62; N, 20.98. Found: C, 64.52; H, 3.78; N, 21.06.

#### 2,6-Dichloro-4-[2-(5-hydroxymethyl-2-furylmethylene)hydrazino]quinazoline (4e)

Yield, 67%; mp < 280°C; ^1^HNMR (DMSO-d6) ppm δ: 11.37 (d, 1H, NH, D_2_O exchangeable), 7.82-7.80 (m, 4H, Ar-H, ArCH = N), 7.68 (d, *J* = 8 Hz, 1H, Ar-H), 7.20 (d, *J* = 8 Hz, 1H, Ar-H), 6.52 (br. s, 1H, OH, D_2_O exchangeable), 4.48 (s, 2H, CH_2_); ^13^CNMR (DMSO-d6) ppm δ: 162.3, 150.5, 140.2, 135.2, 126.7, 126.3, 118.0, 116.2. Calcd. For C_14_H_10_Cl_2_N_4_O_2_ (337.16): C, 49.86; H, 2.98; N, 16.62. Found: C, 50.13; H, 3.17; N, 16.85.

#### 2,6-Dichloro-4-[2-(3-pyridylmethylene)hydrazino]quinazoline (4f)

Yield, 66%; mp 198-200°C; ^1^HNMR (DMSO-d6) ppm δ: 11.38 (d, 1H, NH, D_2_O exchangeable), 8.97 (s, 1H, Ar-H), 8.83-8.65 (m, 2H, Ar-H), 8.54-8.20 (m, 1H, Ar-H), 8.06-7.74 (m, 1H, Ar-H), 7.62-7.44 (m, 2H, Ar-H), 7.22-7.20 (d, 1H, ArCH = N); ^13^CNMR (DMSO-d6) ppm δ: 162.2, 160.0, 150.4, 150.1, 149.2, 140.1, 135.4, 135.1, 126.7, 126.2, 124.6, 124.4, 118.0, 116.1. Calcd. For C_14_H_9_Cl_2_N_5_ (318.16): C, 52.84; H, 2.85; N, 22.01. Found: C, 53.17; H, 3.01; N, 22.19.

#### 4-[2-(5-bromo-1H-3-indolylmethylene)hydrazino]-2,6-dichloroquinazoline (4g)

Yield, 70%; mp < 280°C; ^1^HNMR (DMSO-d6) ppm δ: 12.12 (s, 1H, NH indole, D_2_O exchangeable), 12.00 (s, 1H, NH, D_2_O exchangeable), 8.91 (s, 1H, Ar-H), 8.58-8.42 (m, 2H, Ar-H), 8.06-7.87 (m, 2H, Ar-H), 7.80 (d, 1H, ArCH = N), 7.46 (d, J = 8 Hz, 1H, Ar-H), 7.34 (d, J = 8 Hz 1H, Ar-H); ^13^CNMR (DMSO-d6) ppm δ: 162.3, 155.7, 150.5, 140.2, 136.3, 135.2, 133.8, 126.8, 126.7, 126.3, 126.0, 125.9, 125.6, 124.6, 124.1, 123.3, 118.0. Calcd. For C_17_H_10_BrCl_2_N_5_ (435.10): C, 46.92; H, 2.31; N, 16.09. Found: C, 47.20; H, 2.49; N, 16.31.

#### 2,6-Dichloro-4-[2-(4-quinolinylmethylene)hydrazino]quinazoline (4h)

Yield, 65%; mp < 280°C; ^1^HNMR (DMSO-d6) ppm δ: 11.38 (d, 1H, NH, D_2_O exchangeable), 8.89-8.81 (m, 2H, Ar-H), 8.79-8.75 (m, 2H, Ar-H), 8.06-7.99 (m, 2H, Ar-H), 7.94-7.61 (m, 3H, Ar-H), 7.18 (d, 1H, ArCH = N); ^13^CNMR (DMSO-d6) ppm δ: 162.2, 150.4, 150.0, 148.6, 140.1, 135.2, 129.9, 127.6, 126.7, 126.3, 117.9, 117.1, 116.1. Calcd. For C_18_H_11_Cl_2_N_5_ (368.22): C, 58.70; H, 3.01; N, 19.02. Found: C, 58.92; H, 3.23; N, 19.28.

#### General procedure of preparation of compounds (5a-h)

A solution of bromine (0.02 mL) in glacial acetic acid (0.1 mL) was added to a suspension of anhydrous sodium acetate (0.3 g) and the appropriate 1-arylidene-2-(4-quinazolyl)hydrazine (0.39 mol) in acetic acid (1 mL). This mixture was stirred at room temperature for 30 min. The precipitate formed by pouring on ice/cold 0.5 N NaOH was filtered, washed with water, and recrystallized from ethanol.

#### 5-Chloro-3-(5-hydroxymethyl-2-furyl)-1,2,4-triazolo[4,3-c]quinazoline (5a)

Yield, 30%; mp < 280°C; MS m/z (%): 300 (M^+^, 25), 267 (100). Calcd. For C_14_H_9_ClN_4_O_2_ (300.70): C, 55.92; H, 3.02; N, 18.63. Found: C, 56.17; H, 3.23; N, 18.90.

#### 5-Chloro-3-(3-pyridyl)-1,2,4-triazolo[4,3-c]quinazoline (5b)

Yield, 36%; mp < 280°C; MS m/z (%): 281 (M^+^, 12), 179 (100). Calcd. For C_14_H_8_ClN_5_ (281.70): C, 59.68; H, 2.86; N, 24.86. Found: C, 59.86; H, 2.97; N, 25.11.

#### 3-(5-Bromo-3-indolyl)-5-chloro-1,2,4-triazolo[4,3-c]quinazoline (5c)

Yield, 61%; mp 200-202°C; ^1^HNMR (DMSO-d6) ppm δ: 11.90 (s, 1H, NH, D_2_O exchangeable), 8.92 (s, 1H, Ar-H), 8.50 (d, *J* = 4 Hz, 1H, Ar-H), 8.04-7.90 (m, 4H, Ar-H), 7.47 (d, *J* = 8 Hz, 1H, Ar-H), 7.35 (dd, *J* = 8, 4 Hz, 1H, Ar-H); ^13^CNMR (DMSO-d6) ppm δ: 155.7, 136.4, 133.8, 126.8, 125.6, 124.6, 114.5, 113.7, 112.0; Calcd. For C_17_H_9_BrClN_5_ (398.64): C, 51.21; H, 2.27; N, 17.57. Found: C, 51.38; H, 2.40; N, 17.81.

#### 5-Chloro-3-(4-quinolinyl)-1,2,4-triazolo[4,3-c]quinazoline (5d)

Yield, 52%; mp 220-222°C; ^1^HNMR (DMSO-d6) ppm δ: 9.42-9.40 (m, 2H, Ar-H), 9.10 (d, *J* = 4 Hz, 2H, Ar-H), 9.02 (d, *J* = 8 Hz, 2H, Ar-H), 8.16 (d, *J* = 8 Hz 1H, Ar-H), 8.05 (d, *J* = 4 Hz, 1H, Ar-H), 7.88 (t, 1H, Ar-H), 7.77 (t, 1H, Ar-H); ^13^CNMR (DMSO-d6) ppm δ: 160.2, 151.0, 148.9, 136.9, 130.3, 130.2, 128.3, 125.5, 125.3, 122.3; Calcd. For C_18_H_10_ClN_5_ (331.76): C, 65.16; H, 3.03; N, 21.11. Found: C, 65.37; H, 3.20; N, 21.39.

#### 5,9-Dichloro-3-(5-hydroxymethyl-2-furyl)-1,2,4-triazolo[4,3-c]quinazoline (5e)

Yield, 78%; mp < 280°C; ^1^HNMR (DMSO-d6) ppm δ: 7.82 (d, *J* = 4 Hz, 1H, Ar-H), 7.67 (dd, *J* = 8, 4 Hz, 1H, Ar-H), 7.50 (s, 1H, Ar-H), 7.19 (d, *J* = 8Hz, 1H, Ar-H), 6.93 (s, 1H, Ar-H), 6.45 (s, 1H, OH, D_2_O exchangeable), 4.47 (s, 2H, CH_2_); Calcd. For C_14_H_8_Cl_2_N_4_O_2_ (335.14): C, 50.17; H, 2.40; N, 16.72. Found: C, 50.42; H, 2.63; N, 16.89.

#### 5,9-Dichloro-3-(3-pyridyl)-1,2,4-triazolo[4,3-c]quinazoline (5f)

Yield, 97%; mp 219-221°C; ^1^HNMR (DMSO-d6) ppm δ: 8.98 (s, 1H, Ar-H), 8.83 (s, 1H, Ar-H), 8.56 (d, 1H, Ar-H), 8.23-7.89 (m, 2H, Ar-H), 7.50-7.10 (m, 2H, Ar-H); Calcd. For C_14_H_7_Cl_2_N_5_ (316.15): C, 53.18; H, 2.23; N, 22.15. Found: C, 53.40; H, 2.35; N, 22.41.

#### 3-(5-Bromo-3-indolyl)- 5,9-dichloro-1,2,4-triazolo[4,3-c]quinazoline (5g)

Yield, 98%; mp < 280°C; ^1^HNMR (DMSO-d6) ppm δ: 12.03 (s, 1H, NH, D_2_O exchangeable), 8.91 (s, 1H, Ar-H), 8.57-8.39 (m, 1H, Ar-H), 8.09-7.89 (m, 1H, Ar-H), 7.54-7.44 (m, 2H, Ar-H), 7.43-7.17 (m, 2H, Ar-H); ^13^CNMR (DMSO-d6) ppm δ: 136.6, 134.8, 134.4, 133.1, 132.7, 129.0, 126.1, 126.0, 125.7, 125.1, 124.1, 123.6, 121.4, 116.8, 114.8, 113.7, 113.6; Calcd. For C_17_H_8_BrCl_2_N_5_ (433.09): C, 47.14; H, 1.86; N, 16.17. Found: C, 47.35; H, 2.03; N, 16.43.

#### 5,9-Dichloro-3-(4-quinolinyl)-1,2,4-triazolo[4,3-c]quinazoline (5h)

Yield, 97%; mp < 280°C; ^1^HNMR (DMSO-d6) ppm δ: 9.24 (s, 1H, Ar-H), 8.90-8.50 (m, 2H, Ar-H), 8.11-7.99 (m, 2H, Ar-H), 7.84-7.62 (m, 2H, Ar-H), 7.54-7.40 (m, 1H, Ar-H), 7.21-7.17 (m, 1H, Ar-H); ^13^CNMR (DMSO-d6) ppm δ: 175.7, 150.3, 148.9, 135.0, 130.0, 129.8, 129.6, 126.3, 125.7, 125.4, 123.8; Calcd. For C_18_H_9_Cl_2_N_5_ (366.21): C, 59.03; H, 2.48; N, 19.12. Found: C, 59.27; H, 2.75; N, 19.40.

### *In vitro* PDE7A inhibitory activity assay

All the newly synthesized compounds were evaluated for their *in vitro* PDE7A enzyme inhibitory activity, in comparison with BRL50481 and Theophylline, using the PDE7A Assay Kit (catalog no.60373). The inhibitory activity of the compounds was determined as half maximal inhibitory concentration IC_50_ in μM (Malik et al., 2008; Pekkinen et al., 2008).

### Ligand and protein preparation

The crystal structure of PDE7A catalytic domain in complex with 3-(2,6-difluorophenyl)-2-(methylthio)quinazolin-4(3H)-one was retrieved from the protein data bank (PDB ID: 3G3N). Subsequently, the protein underwent preparation using the “Protein Preparation Wizard” tool in Maestro Schrödinger (Schrödinger Release: LigPrep, 2023). This process involved adding missing hydrogen atoms to the residues, correcting the metal ionization state, and removing water molecules greater than 5 Å from protein residues. Further refinement steps included predicting the pKa for the ionizable residues using PROPKA, and removing water molecules greater than 3 Å that did not participate in water bridges. Next, the protein was energy-minimized by applying the OPLS4 force field.

The compounds were also prepared and energy minimized using the “LigPrep” tool (Castaño et al., 2009). This involved converting the 2D structures to 3D, followed by energy-minimization using the OPLS3 force field. Hydrogen atoms were added, and all possible ionization states and tautomeric forms were generated at a pH of 7.0 ± 0.2 by Epik, including a desalt option. Optimization of hydrogen bonds was carried out by predicting the pKa of ionizable groups using PROPKA.

### Grid generation and molecular docking

A grid box around the active site of PDE7A (PDB ID: 3G3N) was generated utilizing Glide’s Receptor-Grid-Generation tool (Schrödinger Release: Glide, 2023). The box was centered around the co-crystallized ligand using default parameters, which included a van der Waals radius scaling factor 1.0 and partial charge cutoff 0.25. Then, the compounds were docked within the generated grid box using both standard precision (SP) and extra precision (XP) scoring function, sequentially.

### Prime MM-GBSA calculations

Molecular mechanics with generalized born surface area (MM-GBSA) was utilized to predict the relative binding affinities of protein-ligand complexes derived from Glide XP docking, employing the Prime module within the Schrödinger package (Prime, Schrödinger, LLC, New York, NY, 2024). The estimation of relative binding free energy, ΔG_bind_, followed this equation:

$$\Delta G_{\mathrm{bind}}=E_{\mathrm{complex}}\left(\mathrm{minimized}\right)\;-\;\left[E_{\mathrm{ligand}}\left(\mathrm{unbound},\mathrm{minimized}\right)+E_{\mathrm{target}}\left(\mathrm{unbound},\mathrm{minimized}\right)\right]$$

Where ΔG_bind_ represents the predicted relative free energy including both ligand and receptor strain energy. E_complex_ (minimized) denotes the MM-GBSA energy of the minimized complex, while E_ligand_ (unbound, minimized) indicates the MM-GBSA energy of the ligand post-separation from the complex and relaxation. E_receptor_ (unbound, minimized) represents the MM-GBSA energy of the protein after separating it from the ligand. Lower values of ΔG_bind_ indicate stronger binding affinity.

### Molecular dynamic (MD) simulations

The complexes obtained from XP Glide docking were exported for MD simulation using Desmond in Schrödinger suite (Schrödinger Release: Desmond Molecular Dynamics System, 2023). The system builder tool was employed to solvate the system in a TIP3P water model and Na ions were added to maintain physiological ionic concentration. In addition, an orthorhombic simulation box with dimensions of 10 Å × 10 Å × 10 Å around the complex was generated. The MD simulation was run using the OPLS4 force field for a duration of 100 nanosecond (ns) at a temperature of 300 K, pH of 7.0 ± 0.2, and a standard pressure of 1.01325 bar. The ensemble class was set as NPT to ensure constant temperature and pressure throughout the simulation run. Upon completion, the results of the MD simulation were analyzed.

## Results and discussions

### Chemistry

**SCHEME 1:**
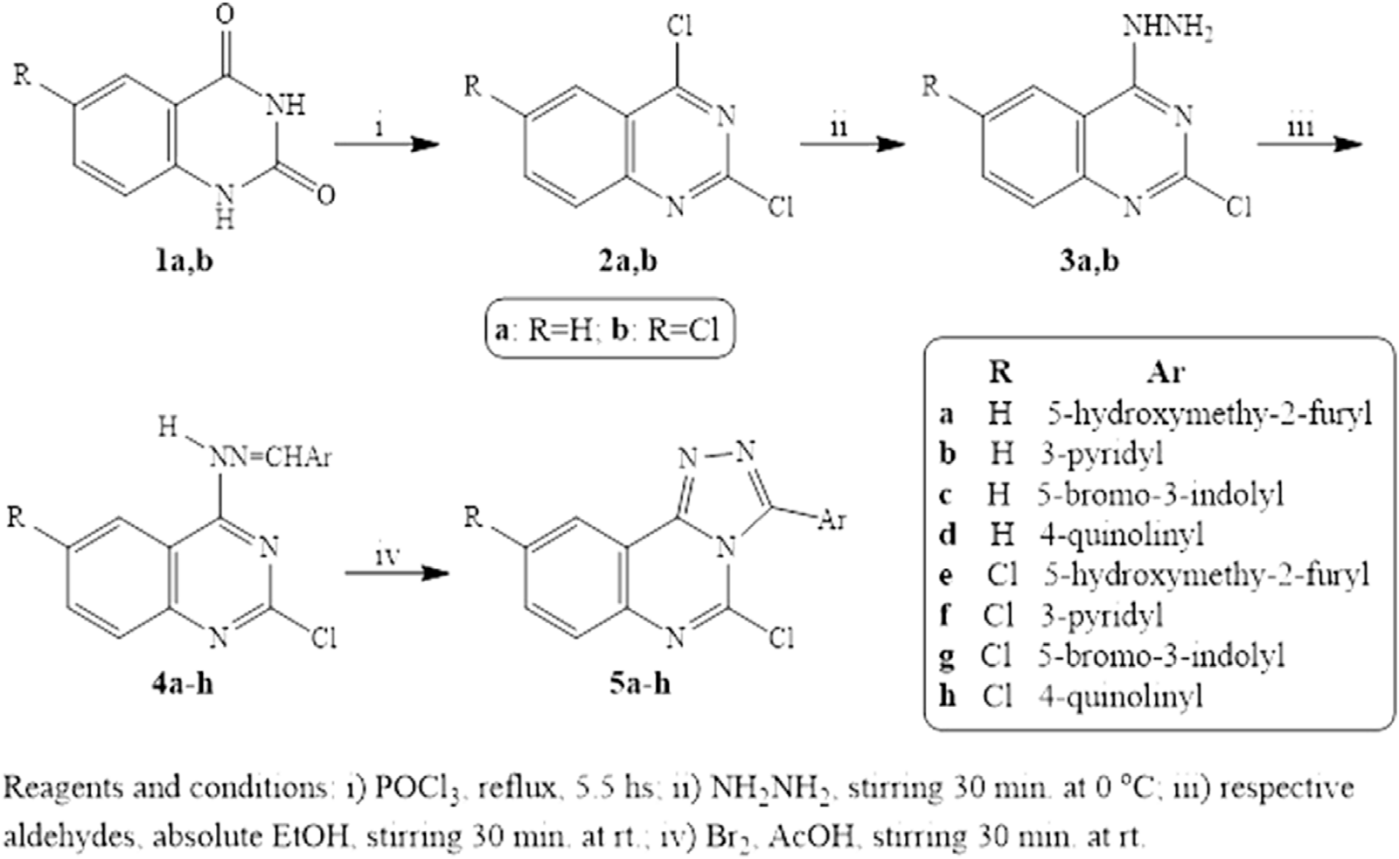
Reagents and Conditions.

The preparation of the target quinazoline derivatives is described in Scheme 1. Anthranilic acid and 5-chloranthranilic acid are the precursors for the construction of the quinazoline skeletons 1a,b according to the reported method (Russo et al., 1966). 2,4-Dichloroquinazolines 2a,b were prepared from 2,4-dioxoquinazolines 1a,b through reaction with a phosphoryl chloride/N,N-dimethyl aniline mixture (Curd et al., 1947; El-kerdawy et al., 1990). Compounds 3a,b were synthesized from the corresponding 2,4-dichloroquinazolines 2a,b through preferential monohydrazinolysis utilizing the higher reactivity of the 4-chloro group over that at the two-position in the quinazoline ring. In this investigation, 4- hydrazonoquinazolines 4a-h were formed through a facile reaction using the selected aldehydes in ethanol with the quinazolylhydrazines 3a,b. Triazoloquinazolines 5a-h were prepared from the corresponding hydrazones by treatment with a mixture of bromine, sodium acetate, and acetic acid. The cyclization step proceeds via an attack on the benzylic carbon by the three-position nitrogen of the quinazoline moiety. The ^1^HNMR spectroscopy of Schiff bases 4a-h shows a band for NH that appeared at δ 11.21-12.60 ppm which was no longer detectable in triazoloquinazolines 5a-h. We concluded that cyclization was achieved.

Novel quinazoline derivatives synthesized in this work were characterized by ^1^HNMR, ^13^CNMR spectroscopy, mass spectroscopy, and elemental analysis. The ^1^HNMR spectra of 5-hydroxymethyl furyl quinazolylhydrazones 4a and 4e revealed the presence of the methylene singlet signal at δ 4.48 ppm, along with an exchangeable OH at δ 6.50-6.52 ppm in addition to increase in aromatic signals assigned for aromatic protons. Moreover, the triazolo derivative of this hydrazone 5e showed a singlet signal of methylene at δ 4.47 ppm in addition to OH at δ 6.45 ppm which was exchangeable with D2O. Furthermore, ^1^HNMR spectra of 5-bromoindolylquinazoline derivatives 4c, 4g, 5c, and 5g showed the presence of NH of indole moiety at δ 11.90-12.12 ppm. It is noteworthy that the 3-aryl substitution of triazoloquinazolines 5a-h showed an increase in the normal pattern of the aromatic carbons in ^13^CNMR spectra of these compounds. In addition, the structures of the newly synthesized compounds were confirmed using elemental analysis of C, H, and N.

### Biological evaluation

#### *In vitro* PDE7A inhibition activity

All the newly synthesized compounds **4a-h** and **5a-h** were evaluated for *in vitro* PDE7A inhibitory activity using a fluorescence polarization PDE7A Assay Kit (Catalog no. 60373; BPS Bioscience) (Malik et al., 2008; Pekkinen et al., 2008). The assay is based on the binding of fluorescent nucleotide, a monophosphate generated by PDE7A to the binding agent. PDE7A catalyzes the hydrolysis of the phosphodiester bond in dye-labeled cyclic adenosine monophosphate (cAMP). Nanoparticle beads selectively bind the phosphate group in the nucleotide product. This increases the size of the nucleotide relative to unreacted cAMP. Since the degree of polarization of a fluorophore is inversely related to its molecular rotation, dyes attached to the slowly rotating nucleotide-bead complexes will not have time to reorient and therefore will emit highly polarized light. Conversely, dyes attached to the rapidly rotating cyclic monophosphates will obtain random orientations and emit light with low polarization.

The PDE7A inhibitor screening assay kit comes in a convenient 384-well format, including purified PDE7A enzyme, fluorescently labeled PDE7A substrate (cAMP), binding agent, and PDE assay buffer for 384 enzyme reactions. The key to the PDE7A Assay Kit is the specific binding agent. Using this kit, only two simple steps on a microtiter plate are required for PDE7A reactions. First, the fluorescently labeled cAMP is incubated with a sample containing PDE7A for 1 h. Second, a binding agent is added to the reaction mix to produce a change in fluorescent polarization that can then be measured using a fluorescence reader. The microtiter plate reader is capable of excitation at wavelengths ranging from 485 ± 15 nm and detection of emitted light ranging from 528 ± 10 nm. Theophylline, a non-selective PDE inhibitor, was used as a reference standard. Moreover, BRL50481, a selective PDE7A inhibitor, was also used to compare the selectivity. The inhibitory activity of the tested compounds was determined as half maximal inhibitory concentration IC_50_ in μM compared to the two reference standards.

**FIGURE 2:**
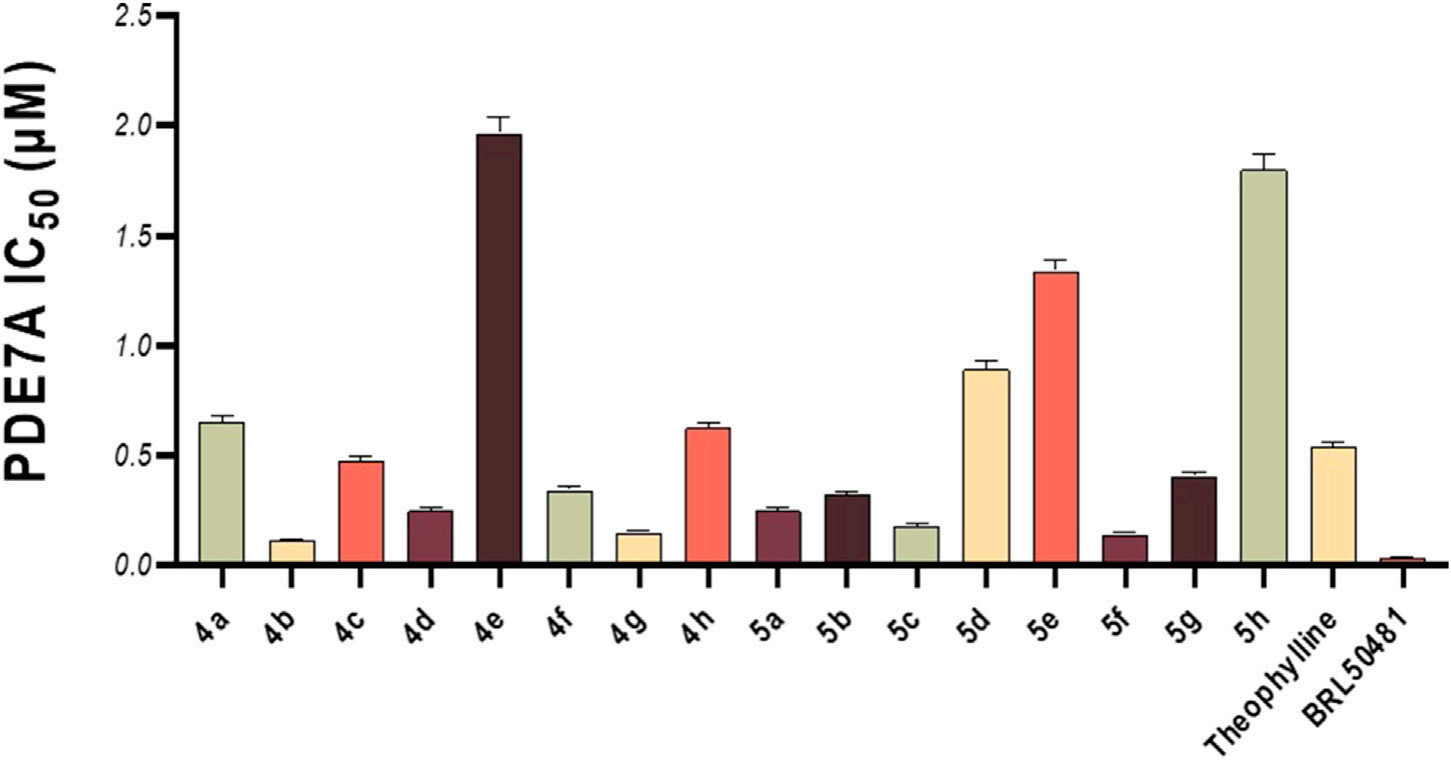
*In vitro* PDE7A inhibitory activity of the tested compounds compared to theophylline and BRL50481.

This *in vitro* testing assay was carried out at the confirmatory diagnostic unit, Vacsera, Egypt, and the results are presented in Table 1 and represented in Figure 2. From the data obtained, it can be deduced that all the synthesized compounds had PDE7A inhibitory activity in the range of IC_50_ values 0.114-1.966 μM. Compounds **4b-d, 4f, 4g, 5a-c, 5f** and **5g** were more potent than Theophylline which is used as a non-selective PDE inhibitor. Among these compounds there were four compounds (**4b, 4g, 5c**, and **5f**) had potencies against PDE7A (IC_50_ 0.114-0.18 μM) comparable to the selective PDE7A inhibitor used in this assay, BRL50481 (IC_50_ = 0.034 μM). Furthermore, Compounds **4c, 4d, 4f, 5a, 5b**, and **5g** had appreciable activities (IC_50_ 0.249-0.478 μM) to the same standard BRL50481. From the biological point of interest, the following structure-activity relationships could be concluded. Halogen atoms are lipophilic substituents commonly used in the design of novel biologically active molecules. In the currently screened ligands, screening results showed that compounds containing chlorine atom are less active than the non-chlorinated analogues with few exceptions. This might be due to the lack of the corresponding lipophilic portion on the receptor. Apart from compound **5a**, compounds bearing the 5-hydroxymethyl-2-furyl moiety (**4a**, **4e**, and **5e**) are among the least effective compounds as PDE7 inhibitors.

**TABLE 1:** *In vitro* PDE7A inhibitory activity of the tested compounds compared to theophylline and BRL50481.

Compound	R	Ar	PDE7A (IC_50_ μM)	SD ±
**4a**	H	5-Hydroxymethyl-2-furyl	0.653	0.025
**4b**	H	3-Pyridyl	0.114	0.004
**4c**	H	5-Bromo-3-indolyl	0.478	0.018
**4d**	H	4-Quinolinyl	0.249	0.009
**4e**	Cl	5-Hydroxymethyl-2-furyl	1.966	0.074
**4f**	Cl	3-Pyridyl	0.343	0.013
**4g**	Cl	5-Bromo-3-indolyl	0.15	0.006
**4h**	Cl	4-Quinolinyl	0.627	0.024
**5a**	H	5-Hydroxymethyl-2-furyl	0.251	0.011
**5b**	H	3-Pyridyl	0.32	0.014
**5c**	H	5-Bromo-3-indolyl	0.18	0.007
**5d**	H	4-Quinolinyl	0.894	0.034
**5e**	Cl	5-Hydroxymethyl-2-furyl	1.338	0.051
**5f**	Cl	3-Pyridyl	0.142	0.005
**5g**	Cl	5-Bromo-3-indolyl	0.407	0.015
**5h**	Cl	4-Quinolinyl	1.802	0.068
**Theophylline**	-	-	0.539	0.024
**BRL50481**	-	-	0.034	0.002

The reason for this may be the absence of an electrophilic region at the receptor to interact with the high electron density on the furan ring as well as the electron pair on the oxygen atom. On the other hand, compounds with the π-deficient pyridine ring (**4b**, **4f**, **5b**, and **5f**) manifested high PDE7 inhibitory potencies. This series of compounds includes the most active ligands, **4b** and **5f**. This high activity might be attributed to the existence of the receptor of a nucleophilic center capable of interacting with the pyridine nucleus. On the contrary, compounds containing the quinoline nucleus (**4h**, **5d**, and **5h**), despite being π-deficient, displayed a very low PDE7 inhibitory effect. The reason behind this may lie in the fact that the quinoline ring is bigger than the pyridine moiety, which may not fit the receptor site. Finally, on average, quinazoline pharmacophore in which 5-bromo-3-indolyl functionality was incorporated (**4g**, **5c**, and **5g**) showed good activity as inhibitors of PDE7.

### Molecular docking study and MM-GBSA binding energy calculations

To further rationalize the biological results, a molecular docking study and MM-GBSA calculation were carried out to evaluate the binding affinities of the top compounds, **4b** and **5f**, in addition to BRL50481, which was used as a reference compound in the biological assays. The compounds were docked against the PDE7A binding site using Glide docking software within Schrödinger suite (Schrödinger Release: Glide, 2023; Friesner et al., 2004; Halgren et al., 2004). Prior to the molecular docking, the catalytic domain of PDE7A was retrieved from the Protein Data Bank (PDB ID 3G3N) (Castaño et al., 2009). Subsequently, the structure was optimized and energy-minimized using Schrödinger’s Protein Preparation Wizard (Schrödinger Release: LigPrep, 2023; Castaño et al., 2009).

The compounds underwent preparation by converting their 2D structures into energy-minimized 3D structures and generating all possible ionization and tautomeric starts using Schrödinger’s LigPrep tool (Schrödinger Release: LigPrep, 2023; Sastry et al., 2013). For the docking experiment, a grid box around the binding site was generated to locate the docking site using a Receptor Grid Generation tool in Maestro (Schrödinger Release: Glide, 2023; Friesner et al., 2004). The docking experiment employed a standard precision (SP) followed by the extra-precision (XP) scoring function to ensure maximum accuracy (Friesner et al., 2006). The docking scores were calculated using various scoring functions, including emodel, gscore, and XP gscore. The emodel scoring ranks the compounds based on the best docked pose. Subsequently, these poses were ranked based on their gscore, while those generated by Glide XP were ranked using the XP gscore.

Typically, Glide utilizes the gscore scoring function to rank the docked compounds. The resultant docked complexes were subjected to MM-GBSA calculations using Prime module in Maestro (Prime, Schrödinger, LLC, New York, NY, 2024). MM-GBSA calculate the free binding energy of the protein-ligand interactions by integrating molecular mechanics (MM) calculations for their interaction with a continuum solvent model called generalized born (GB) and surface area (SA) terms. A higher negative value for binding free energy indicates a stronger binding affinity. Table 2 presents the docking results and MM-GBSA scores of compounds 4b, 5f and BRL50481. The docking scores indicate that compounds 4b and 5f exhibited higher docking scores compared to BRL50481, with gscore values of −6.887, and −6.314, respectively, in contrast to BRL50481s score of −5.096. Notably, compound 4b showed the most favorable docking score. Furthermore, MM-GBSA calculations revealed more negative values for compounds 4b and 5f (−45.3 and −46.68 kcal/mol, respectively) compared to BRL5048 (−31.52 kcal/mol). These results indicate that compounds 4b and 5f possess superior predicted binding affinity to PDE7A.

**TABLE 2:** Docking results and MM-GBSA scores of 4b, 5f, and BRL50481with PDE7A.

Compound	Docking score	Glide gscore	Glide emodel	XP GScore	MM-GBSA (kcal/mol)
**4b**	−6.887	−6.887	−50.199	−6.887	−45.3
**5f**	−6.313	−6.314	−45.096	−6.314	−46.68
**BRL50481**	−5.096	−5.096	−33.927	−5.096	−31.52

**FIGURE 3:**
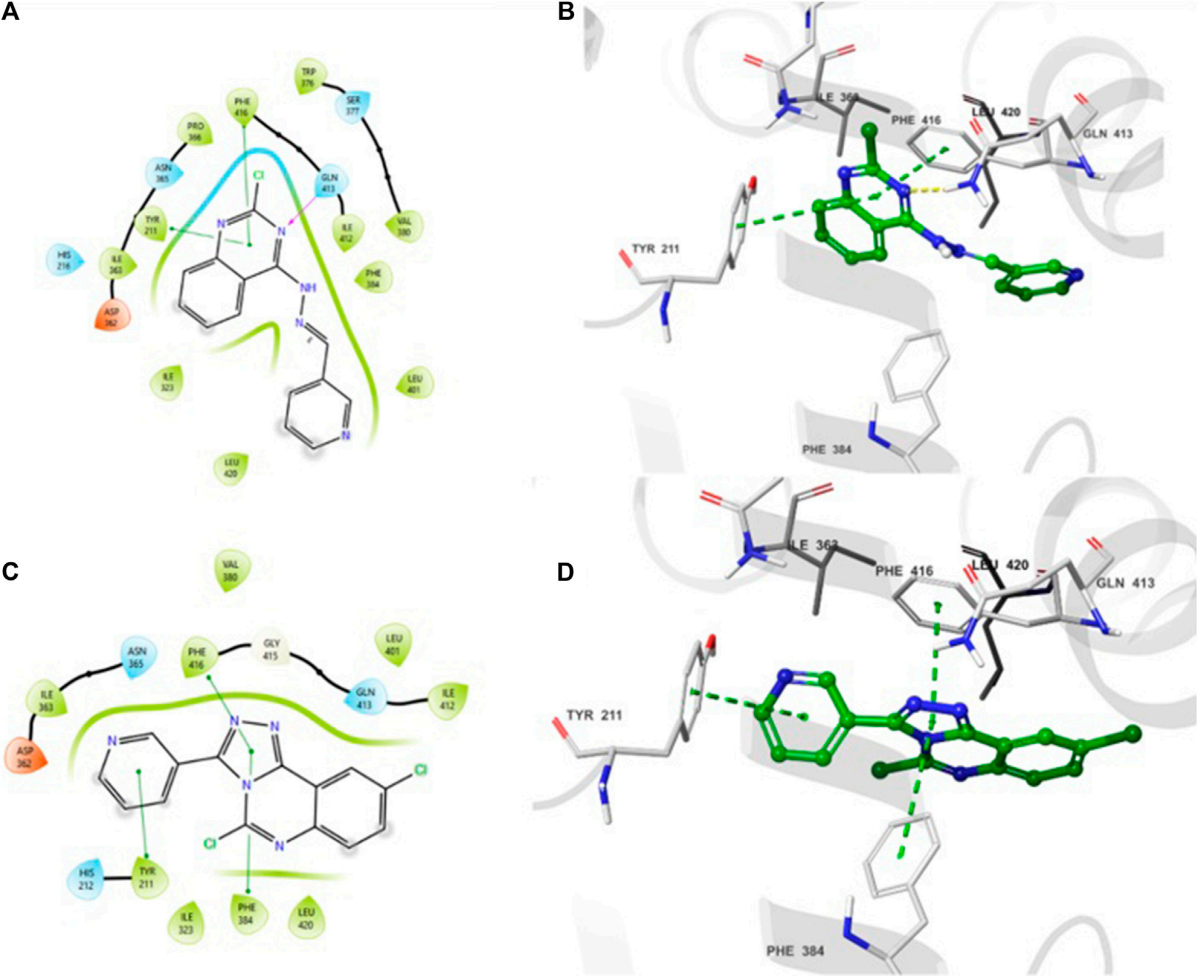
2D- and 3D-interactions of the docked compounds at the binding site of PDE7A **(A)** 2D interaction of **4b** **(B)** 3D interaction of **4b** **(C)** 2D interaction of **5f** **(D)** 3D interaction of **5f**.

The 2D- and 3D-binding interactions of **4b** and **5f** at the binding site of PDE7A. Compound **4b** (Figures 3A, B) showed that the pyrimidine ring of quinazoline formed a π-π stacking interactions with Phe416 and Tyr211. Additionally, the nitrogen atom of the quinazoline ring participated in hydrogen bond interaction with Gln413. Compound **5f** (Figures 3C, D) interacted through π-π stacking, where the triazole ring interacted with Phe416 and Phe384, while the pyridine ring interacted with Tyr211. Both compounds exhibited interaction patterns similar to those reported for BRL50481, involving π-π stacking interactions with Phe416. Additionally, they demonstrated hydrophobic interactions with Phe384, Val380, Leu401, and Phe416 along with a hydrogen bond interaction with Gln413 (Elfeky et al., 2020). The combination of docking analysis and MM-GBSA calculations offers compelling evidence supporting the efficacy of compounds 4b and 5f as potential candidate for further exploration as novel PDE7A inhibitor.

#### Molecular dynamic simulations

**FIGURE 4:**
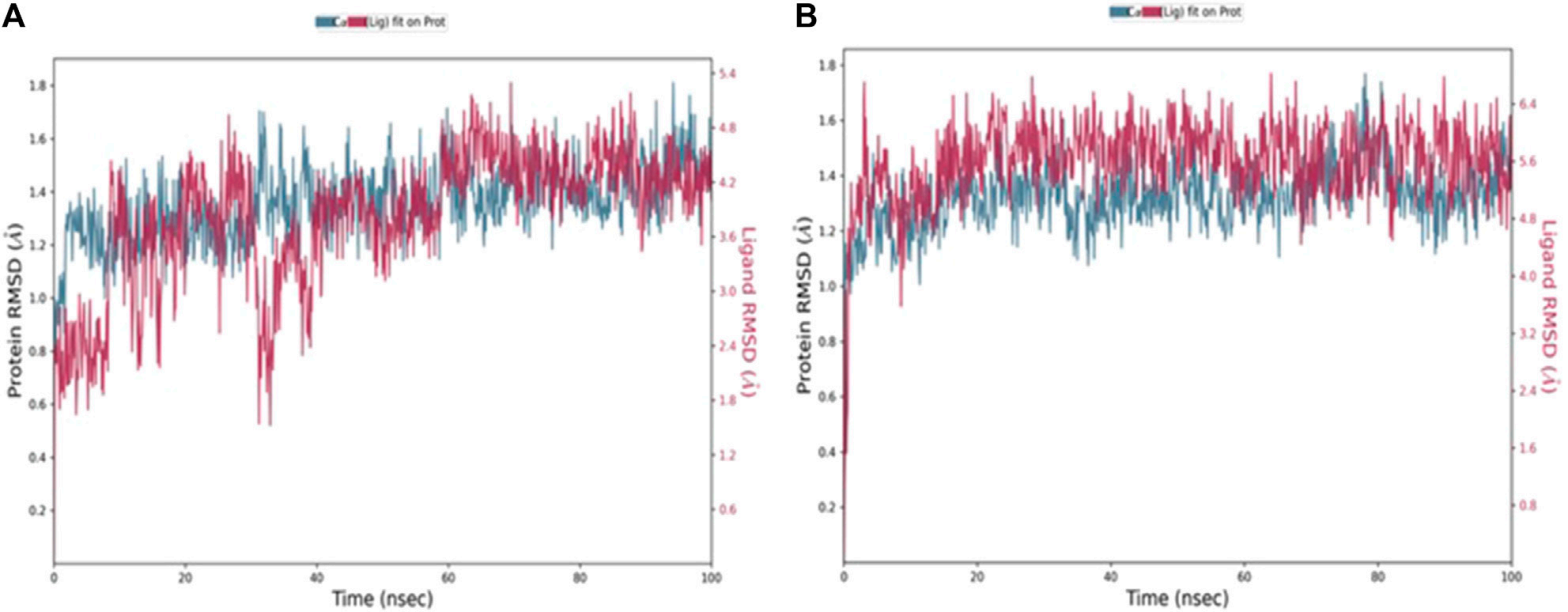
RMSD values throughout 100 ns MD simulations of **(A)** **4b** and **(B)** **5f** bound with PD7A. The left y-axis of the plot indicates the RMSD values for the Cα protein, whereas the right y-axis represents the ligand RMSD. The RMSD graph for Cα and ligand fit are shown in blue and red color, respectively.

**FIGURE 5:**
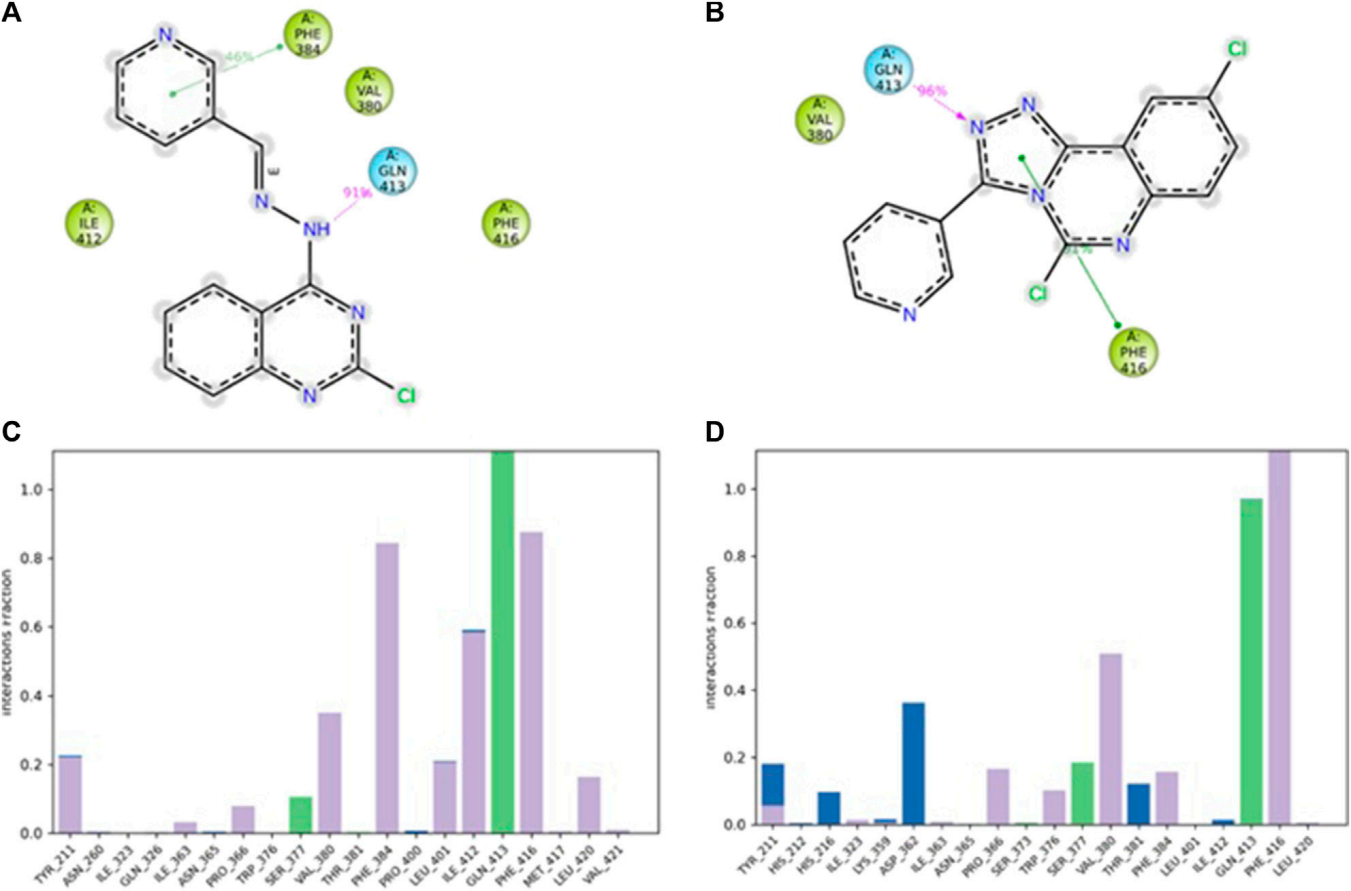
The interaction of compounds **4b** and **5f** in complex with PDE7A throughout the simulation. **(A)** 2D schematic representation of **4b** interaction with PDE7A that occurred for > 30% of the simulation time. **(B)** 2D schematic representation of **5f** interaction with PDE7A that occurred for > 30% of the simulation time. The Stacked bar chart represents **(C)** PDE7A–**4b** and **(D)** PDE7A–**5f** interactions. The interactions were classified into ionic, water bonds, hydrophobic, and hydrogen bonds, and each was classified into subtypes. The interactions were normalized throughout the trajectory; for example, a value of 0.8 suggested that the specific interaction was maintained across 80% of the simulation time. Values over 1.0 indicated that some protein residue might make multiple interactions of the same subtype with the ligand.

MD simulation is a computational tool utilized to further assess a protein-ligand complex’s dynamic behavior and conformational stability under simulated physiological conditions (Hollingsworth and Dror, 2018). Following the molecular docking study, 100 ns MD simulations was conducted for compounds, **4b** and **5f**, using Desmond from the Schrödinger suite (Schrödinger Release: Desmond Molecular Dynamics System, 2023; Bowers et al., 2006). Based on the results obtained from the MD simulation, a simulation interaction diagram was generated including root mean square deviation (RMSD) and a Protein–Ligand interaction plot. The RMSD is a parameter that gives insight into the conformational changes of the protein and ligand framework by plotting the calculated deviation of the PDE7A backbone and the ligands against the simulation time (ns). Figure 4 represents the RMSD of the protein bound to **4b** (Figure 4A) and **5f** (Figure 4B) throughout 100 ns simulation. The backbone of both complexes showed insignificant fluctuation with RMSD values of about 0.8 Å. The RMSD value for **4b** fitted on the protein (Figure 4A) showed minimal fluctuation on the first 40 ns with an RMSD value of 3 Å before stabilizing throughout the rest of the simulation (RMSD 2 Å), while that of **5f** (Figure 4B) exhibited stability with an RMSD value of 2.3 Å across the entire simulation trajectory. The RMSD values for both complexes are within the acceptable ranges of (1-3 Å), suggesting the stability of the docked complex during the 100 ns simulation duration.

A 2D schematic representation (Figures 5A, B) was generated to show the interactions between each compound and the residues at the binding site. Only interactions that occurred for over 30% of the simulation time were taken into account. The binding interaction of **4b** (Figure 5A) showed that the hydrazine nitrogen interacted with Gln413 (91%) and the pyridine ring interacted with Phe384 (46%). The interaction diagram of compound **5f** (Figure 5B) showed that the triazole ring interacted with Phe416 (91%) and Gln413 (96%). The analysis of PDE7A-**4b** and PDE7A-**5f** interactions was also illustrated as stacked bar charts in Figures 5C, D, respectively. The charts represent the residues contact with the compound and the interaction throughout the entire duration of the MD trajectory. It was observed that both compounds shared some amino acid residues that were conserved for the entire simulation run. The PDE7A residue contacts with **4b** (Figure 5C) showed that Gln413 had a value of more than 0.1, indicating that Gln413 formed hydrogen bond interactions that persisted for more than 100% of the simulation time. This could be due to the formation of multiple hydrogen bonds of the same subtypes, such as backbone acceptor, backbone donor, side-chain acceptor, and side-chain donor.

**FIGURE 6:**
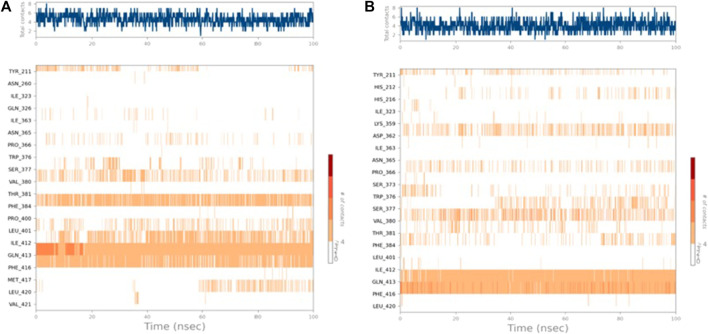
Timeline representation of **(A)** PDE7A–**4b** and **(B)** PDE7A–**5f** interactions. The top panel represents the total number of specific interactions of the protein with the ligand during the MD trajectory. The bottom panel showed the residue interactions with the ligand in each trajectory frame. The dark orange colour indicates the presence of more than one interaction between some residues and the ligand.

The residues Phe416 and Phe384 were involved in π-π stacking interactions that lasted for over 80%, while Ile 412 formed a hydrophobic interaction that remained for 60% of the simulation time. In the case of PDE7A complexed with **5f** (Figure 5D), the residue contacts showed that Gln413 formed a hydrogen bond interaction that remained for over 90%. Additionally, Phe416 was involved in π-π stacking interaction that lasted for more than 100% of the simulation time. The timeline representation of the residue contacts (Figures 6A, B) depicts, in the top panel, the total specific contacts between the ligand and the protein over the MD trajectory. The bottom panel illustrates the protein residues interacting with the ligand at each trajectory frame. Notably, Gln413, Phe416, Phe384, and Ile412 exhibit multiple specific contact with compound **4b**, evident by the darker shade of orange in the trajectory plot (Figure 6A), emphasizing the stability of these interactions. For compound **5f**, Figure 6B shows consistent involvement in multiple specific contacts with only Gln413 and Phe416 throughout the simulation. Based on the results, the mode of interaction of the presented compounds is similar to the previously reported inhibitors of PDE7A, which include hydrogen bonding with Gln413 and hydrophobic interaction with different amino acids, such as Phe416 and Phe384.

## Conclusion

In the present work, hydrazinoquinazolines **4a-h** and triazoloquinazolines **5a-h** were synthesized. All these new 16 compounds were screened for PDE7A inhibition activity using BRL50481 and theophylline as standards for *in vitro* assay. Compounds **4b** and **5f** were the highest selective PDE7A inhibitors among the prepared quinazoline derivatives. Molecular docking and molecular dynamic simulations studies of the mentioned compounds indicate perfect fitting and good interactions with the pocket of the PDE7A enzyme. These observations suggest that compounds **4b** and **5f** represent new scaffolds to design potent PDE7A inhibitors in anti-inflammatory therapy.

## Data Availability

The original contributions presented in the study are included in the article/Supplementary Material, further inquiries can be directed to the corresponding author.
