# Supplementary material for: Design, synthesis, molecular docking, and molecular dynamic studies of novel quinazoline derivatives as phosphodiesterase 7 inhibitors
Source: Front Pharmacol. 2024 Apr 22;15:1389076. doi: 10.3389/fphar.2024.1389076 (PMC11070508; doi:10.3389/fphar.2024.1389076)
Supplement: Supplementary file 1 [file DataSheet2.pdf]

Afaf ElMalah_H_15

Microanalytical Unit - FOPCU - NMR laboratory
www.pharma.cu.edu.eg dir-mau.fopcu@pharma.cu.edu.eg

BRUKER

4a

¹HNMR

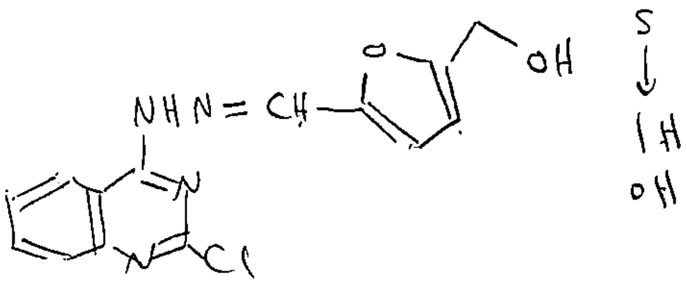


```
Current Data Parameters
NAME      Afaf ElMalah_H_15
EXPNO                   10
PROCNO                   1

F2 - Acquisition Parameters
Date_             20230215
Time                  5.44
INSTRUM              spect
PROBHD   5 mm PABBO BB/
PULPROG               zg30
TD                   65536
SOLVENT               DMSO
NS                      32
DS                       2
SWH             8012.820 Hz
FIDRES          0.122266 Hz
AQ             4.0894465 sec
RG                   91.58
DW                  62.400 usec
DE                    6.50 usec
TE                   298.1 K
D1            1.00000000 sec
TD0                      1

======== CHANNEL f1 ========
SFO1         400.1924713 MHz
NUC1                    1H
P1                   15.00 usec
PLW1         10.39999962 W

F2 - Processing parameters
SI                   65536
SF           400.1900000 MHz
WDW                     EM
SSB      0
LB                    0.30 Hz
GB       0
PC                    1.00
```

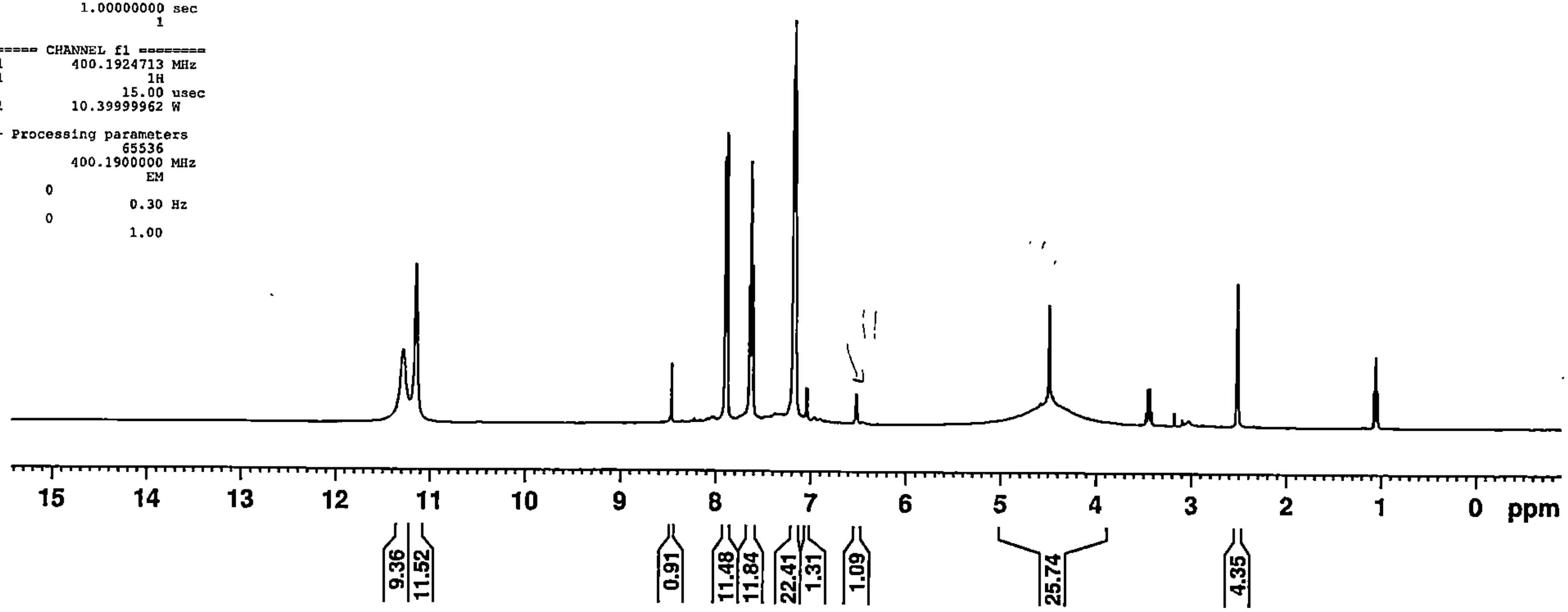

Afaf ElMalah_H_15_D2O

Microanalytical Unit - FOPCU - NMR laboratory
www.pharma.cu.edu.eg dir-mau.fopcu@pharma.cu.edu.eg

BRUKER

4a
^{1}HNMR + D2O

```
Current Data Parameters
NAME      Afaf ElMalah_H_15_D2O
EXPNO                 10
PROCNO                 1

F2 - Acquisition Parameters
Date_           20230215
Time               13.16
INSTRUM            spect
PROBHD   5 mm PABBO BB/
PULPROG             zg30
TD                 65536
SOLVENT             DMSO
NS                    32
DS                     2
SWH            8012.820 Hz
FIDRES         0.122266 Hz
AQ            4.0894465 sec
RG                106.37
DW                62.400 usec
DE                  6.50 usec
TE                 298.0 K
D1            1.00000000 sec
TD0                    1

======== CHANNEL f1 ========
SFO1         400.1924713 MHz
NUC1                  1H
P1                 15.00 usec
PLW1         10.39999962 W

F2 - Processing parameters
SI                 65536
SF           400.1900000 MHz
WDW                   EM
SSB      0
LB                  0.30 Hz
GB       0
PC                  1.00
```

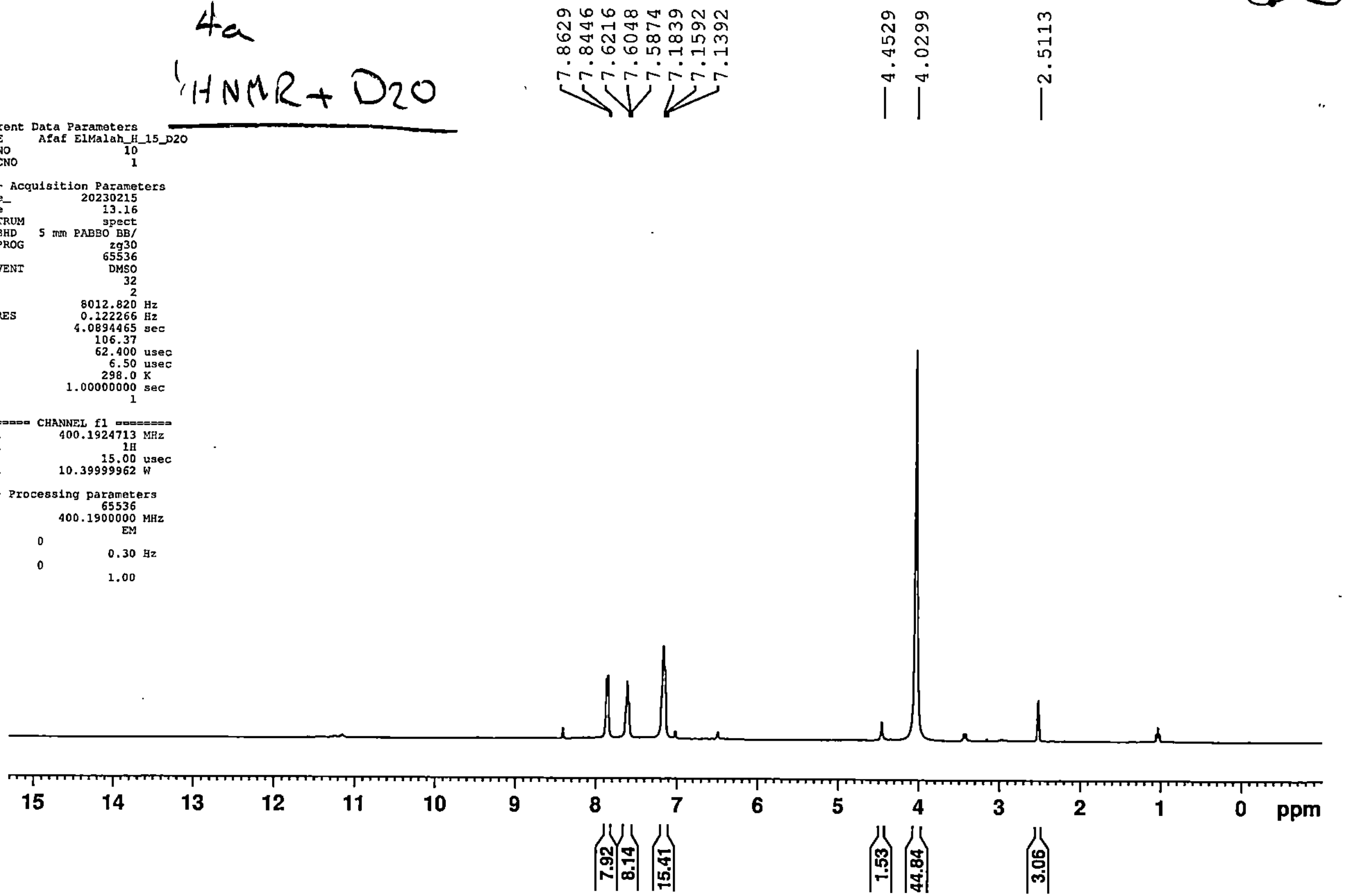

Afaf ElMalah_C_15

Microanalytical Unit - FOPCU - NMR laboratory
www.pharma.cu.edu.eg dir-mau.fopcu@pharma.cu.edu.eg

BRUKER

4a
^{13}CNMR

```
Current Data Parameters
NAME        Afaf ElMalah_C_15
EXPNO                    10
PROCNO                    1

F2 - Acquisition Parameters
Date_              20230215
Time                   6.54
INSTRUM               spect
PROBHD    5 mm PABBO BB/
PULPROG             zgpg30
TD                   65536
SOLVENT               DMSO
NS                    1200
DS                       4
SWH              24038.461 Hz
FIDRES            0.366798 Hz
AQ               1.3631488 sec
RG                  202.37
DW                  20.800 usec
DE                    6.50 usec
TE                   298.1 K
D1              2.00000000 sec
D11             0.03000000 sec
TD0                      1

======== CHANNEL f1 ========
SFO1           100.6379178 MHz
NUC1                   13C
P1                   10.00 usec
PLW1           45.00000000 W

======== CHANNEL f2 ========
SFO2           400.1916008 MHz
NUC2                    1H
CPDPRG[2           waltz16
```

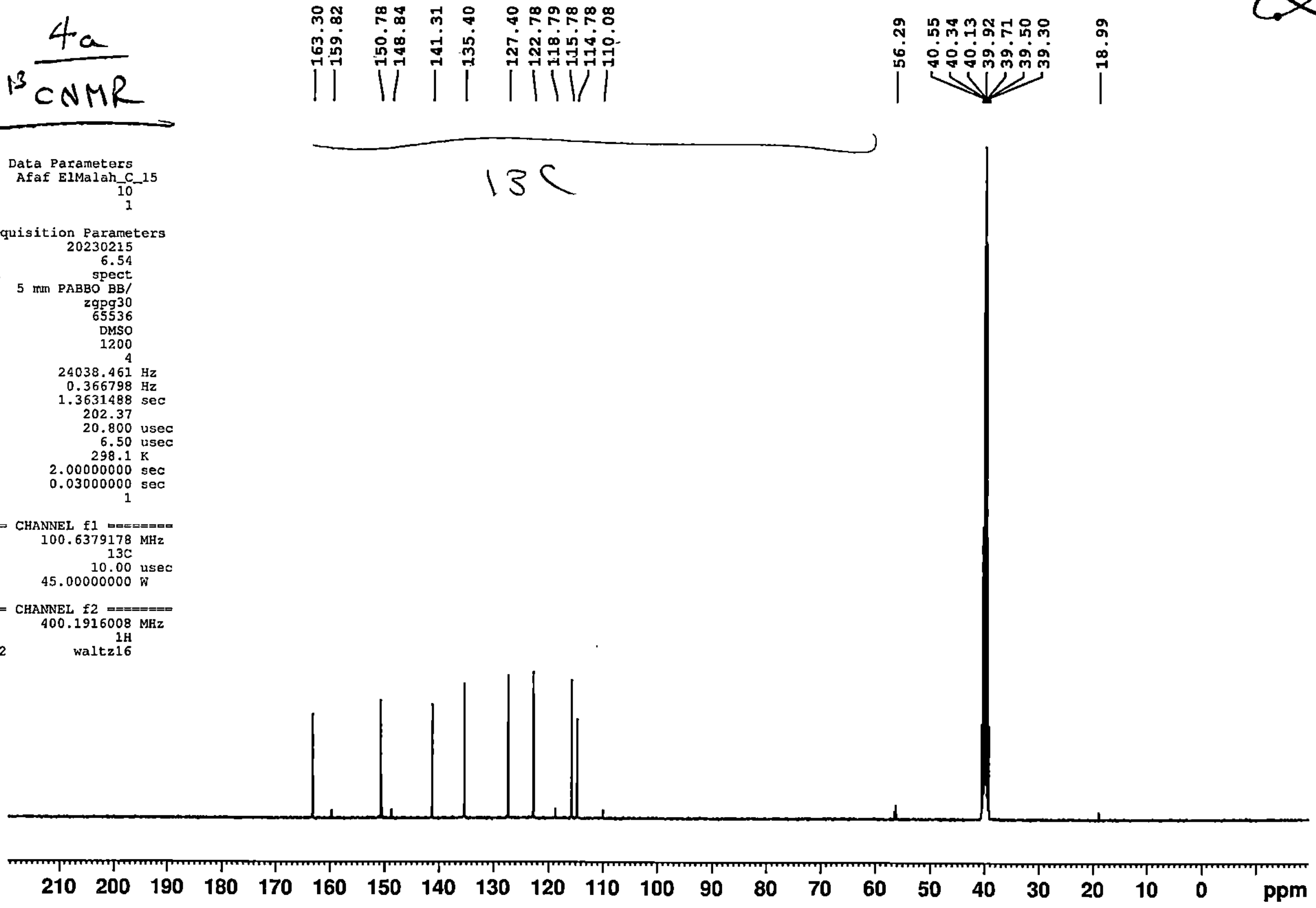

Afaf ElMalah_H_13

4b

^{1}HNMR

Microanalytical Unit - FOPCU - NMR laboratory
www.pharma.cu.edu.eg dir-mau.fopcu@pharma.cu.edu.eg

BRUKER

```
Current Data Parameters
NAME        Afaf ElMalah_H_13
EXPNO                      10
PROCNO                      1

F2 - Acquisition Parameters
Date_              20230215
Time                   4.30
INSTRUM               spect
PROBHD   5 mm PABBO BB/
PULPROG                zg30
TD                    65536
SOLVENT                DMSO
NS                       32
DS                        2
SWH            8012.820 Hz
FIDRES         0.122266 Hz
AQ            4.0894465 sec
RG                    91.58
DW               62.400 usec
DE                 6.50 usec
TE                298.1 K
D1         1.00000000 sec
TD0                       1

======== CHANNEL f1 ========
SFO1       400.1924713 MHz
NUC1                     1H
P1                15.00 usec
PLW1        10.39999962 W

F2 - Processing parameters
SI                    65536
SF         400.1900000 MHz
WDW                      EM
SSB       0
LB                  0.30 Hz
GB        0
PC                     1.00
```

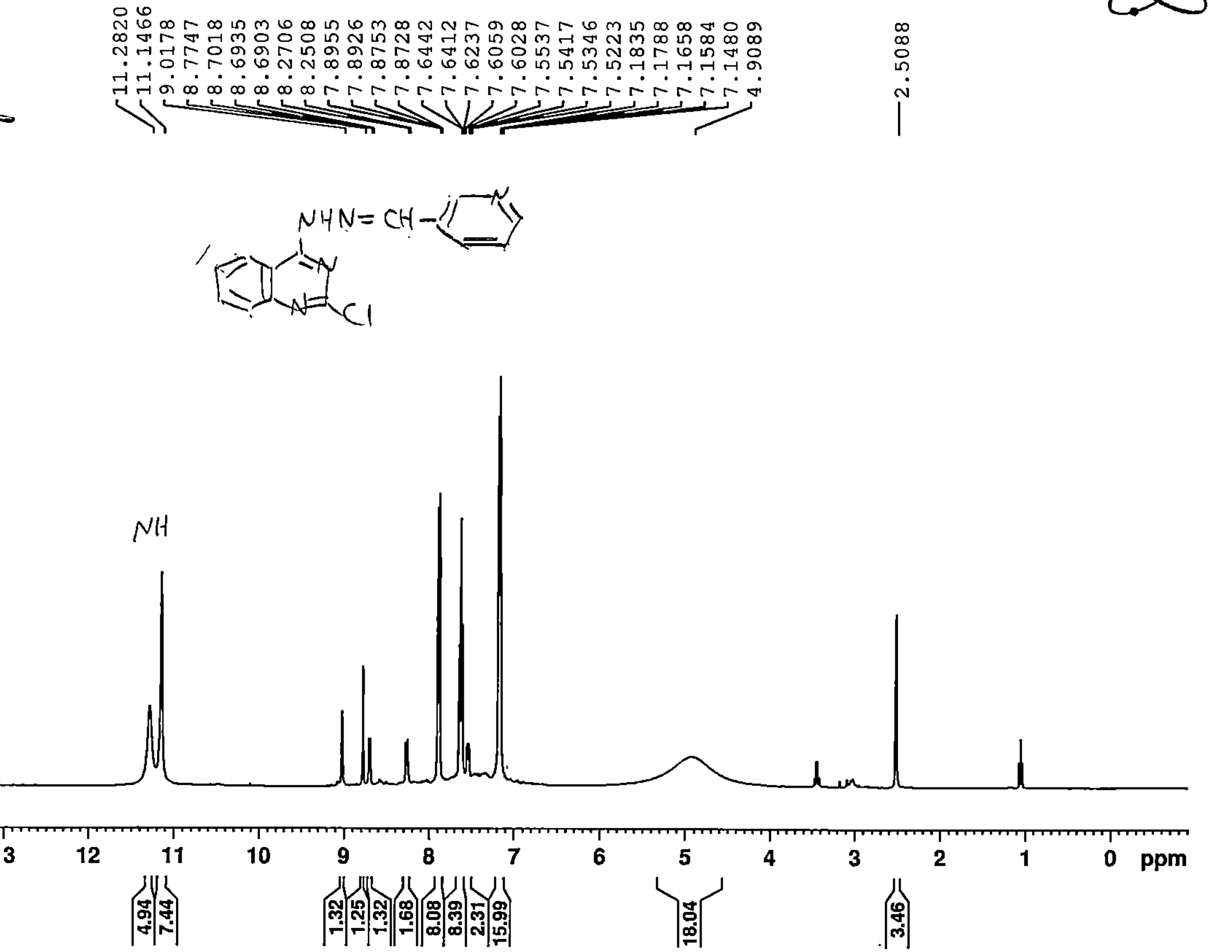

Afaf ElMalah_H_13_D2O

Microanalytical Unit - FOPCU - NMR laboratory
www.pharma.cu.edu.eg dir-mau.fopcu@pharma.cu.edu.eg

BRUKER

4b
1HNMR+D2O

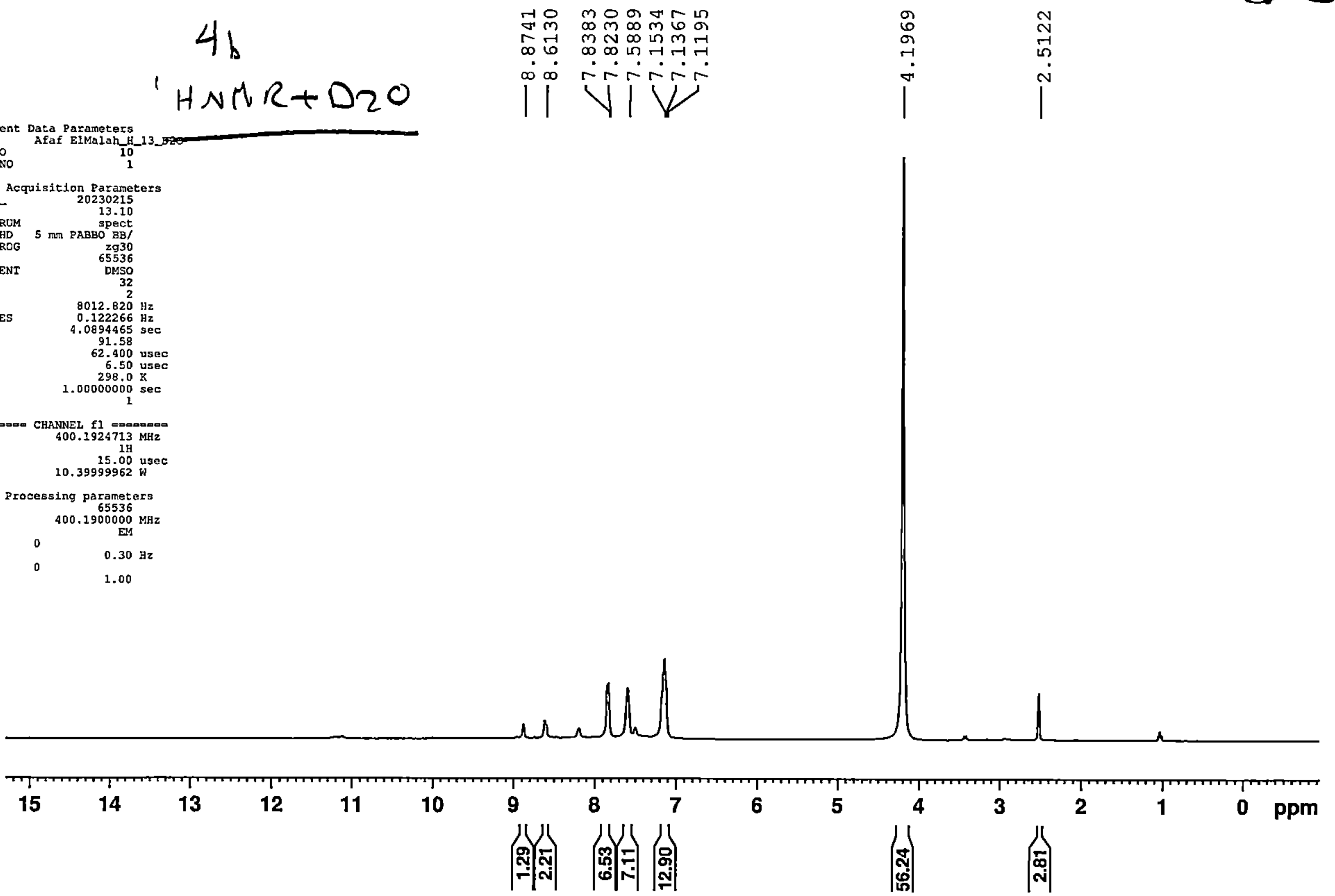

Current Data Parameters
NAME Afaf ElMalah_H_13_D2O
EXPNO 10
PROCNO 1

F2 - Acquisition Parameters
Date_ 20230215
Time 13.10
INSTRUM spect
PROBHD 5 mm PABBO BB/
PULPROG zg30
TD 65536
SOLVENT DMSO
NS 32
DS 2
SWH 8012.820 Hz
FIDRES 0.122266 Hz
AQ 4.0894465 sec
RG 91.58
DW 62.400 usec
DE 6.50 usec
TE 298.0 K
D1 1.00000000 sec
TD0 1

======== CHANNEL f1 ========
SFO1 400.1924713 MHz
NUC1 1H
P1 15.00 usec
PLW1 10.39999962 W

F2 - Processing parameters
SI 65536
SF 400.1900000 MHz
WDW EM
SSB 0
LB 0.30 Hz
GB 0
PC 1.00

Afaf ElMalah_C_13

Microanalytical Unit - FOPCU - NMR laboratory
www.pharma.cu.edu.eg dir-mau.fopcu@pharma.cu.edu.eg

4b
^{13}CNMR

```
Current Data Parameters
NAME      Afaf ElMalah_C_13
EXPNO                   10
PROCNO                   1

F2 - Acquisition Parameters
Date_             20230215
Time                  5.39
INSTRUM              spect
PROBHD   5 mm PABBO BB/
PULPROG            zgpg30
TD                  65536
SOLVENT              DMSO
NS                   1200
DS                      4
SWH             24038.461 Hz
FIDRES           0.366798 Hz
AQ              1.3631488 sec
RG                 202.37
DW                 20.800 usec
DE                   6.50 usec
TE                  298.0 K
D1             2.00000000 sec
D11            0.03000000 sec
TD0                     1

======== CHANNEL f1 ========
SFO1          100.6379178 MHz
NUC1                  13C
P1                  10.00 usec
PLW1          45.00000000 W

======== CHANNEL f2 ========
SFO2          400.1916008 MHz
NUC2                   1H
CPDPRG[2          waltz16
```

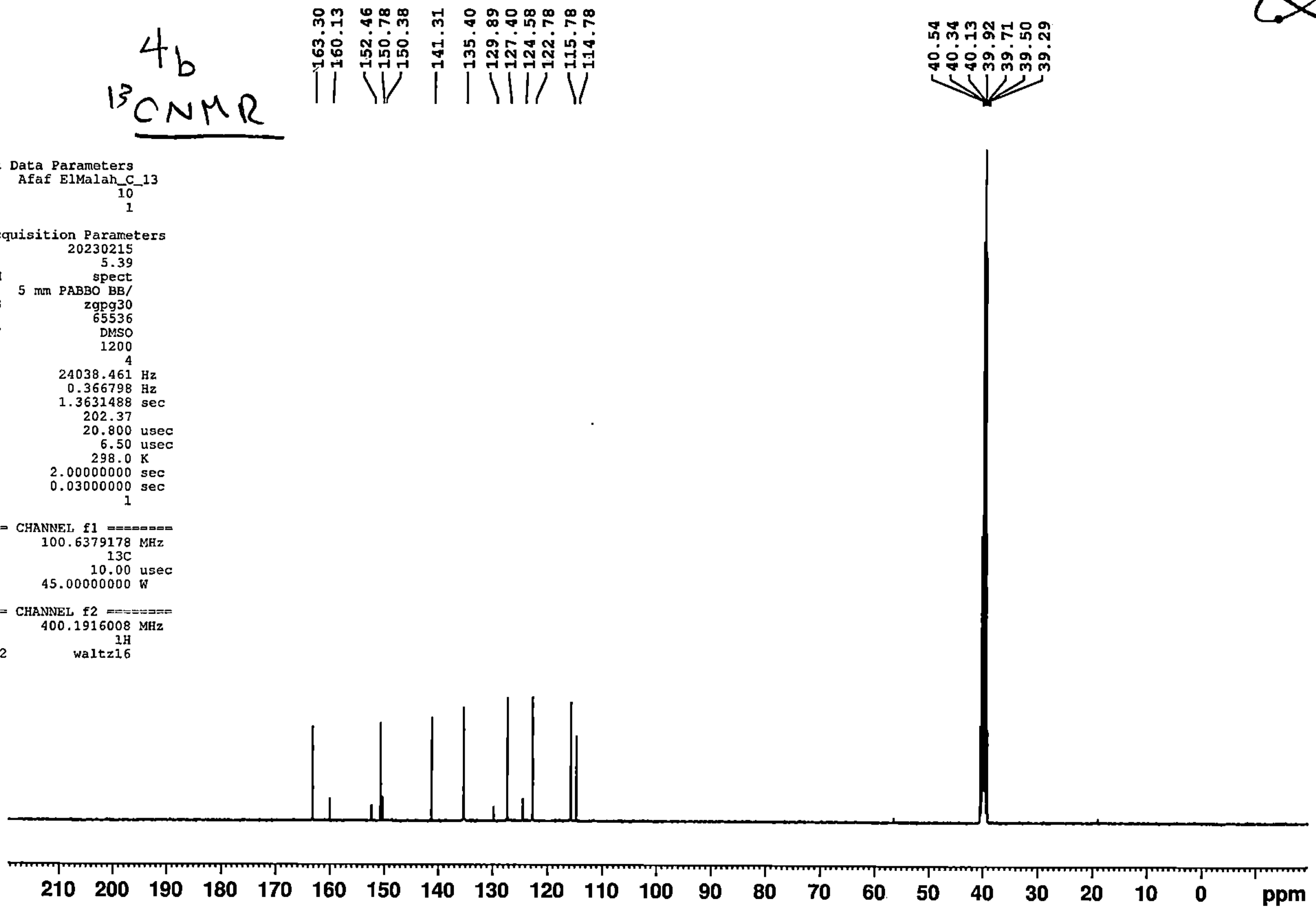

Afaf ElMalah_H_11

Microanalytical Unit - FOPCU - NMR laboratory
www.pharma.cu.edu.eg dir-mau.fopcu@pharma.cu.edu.eg

BRUKER

4c
^{1}H NMR

```
Current Data Parameters
NAME     Afaf ElMalah_H_11
EXPNO                  10
PROCNO                  1

F2 - Acquisition Parameters
Date_            20230215
Time                 3.15
INSTRUM             spect
PROBHD   5 mm PABBO BB/
PULPROG              zg30
TD                  65536
SOLVENT              DMSO
NS                     32
DS                      2
SWH            8012.820 Hz
FIDRES         0.122266 Hz
AQ            4.0894465 sec
RG                 129.43
DW               62.400 usec
DE                 6.50 usec
TE                298.1 K
D1           1.00000000 sec
TD0                     1

======== CHANNEL f1 ========
SFO1        400.1924713 MHz
NUC1                   1H
P1                15.00 usec
PLW1        10.39999962 W

F2 - Processing parameters
SI                  65536
SF          400.1900000 MHz
WDW                    EM
SSB      0
LB                 0.30 Hz
GB       0
PC                   1.00
```

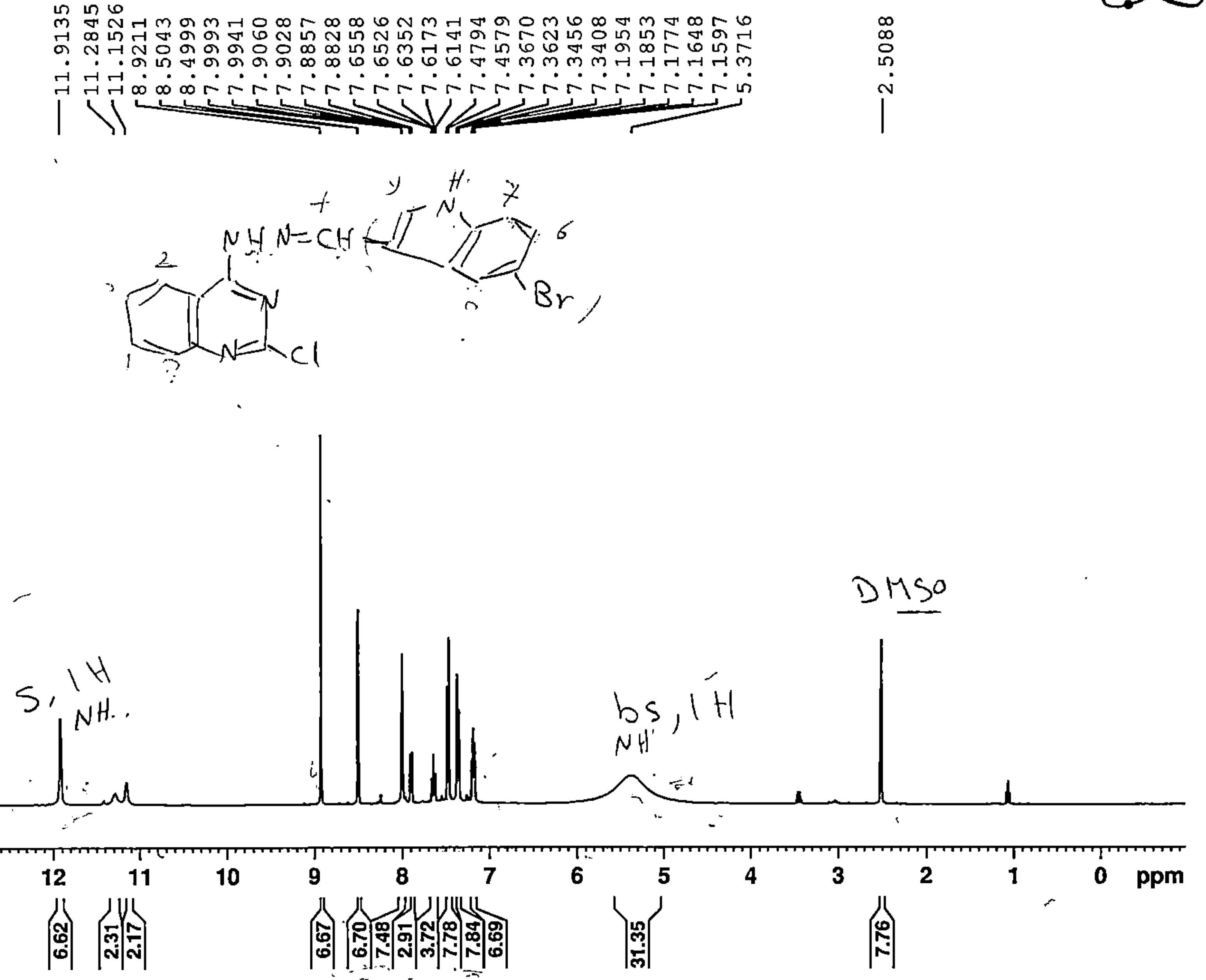

Afaf ElMalah_H_11_D2O

Microanalytical Unit - FOPCU - NMR laboratory
www.pharma.cu.edu.eg dir-mau.fopcu@pharma.cu.edu.eg

BRUKER

4c

1HNMR+D2O

```
Current Data Parameters
NAME     Afaf ElMalah_H_11_D2O
EXPNO              10
PROCNO              1

F2 - Acquisition Parameters
Date_          20230215
Time              13.05
INSTRUM           spect
PROBHD   5 mm PABBO BB/
PULPROG            zg30
TD                65536
SOLVENT            DMSO
NS                   32
DS                    2
SWH            8012.820 Hz
FIDRES         0.122266 Hz
AQ            4.0894465 sec
RG               146.06
DW               62.400 usec
DE                 6.50 usec
TE                298.1 K
D1           1.00000000 sec
TD0                   1

======== CHANNEL f1 ========
SFO1        400.1924713 MHz
NUC1                 1H
P1                15.00 usec
PLW1        10.39999962 W

F2 - Processing parameters
SI                65536
SF          400.1900000 MHz
WDW                  EM
SSB       0
LB                 0.30 Hz
GB        0
PC                 1.00
```

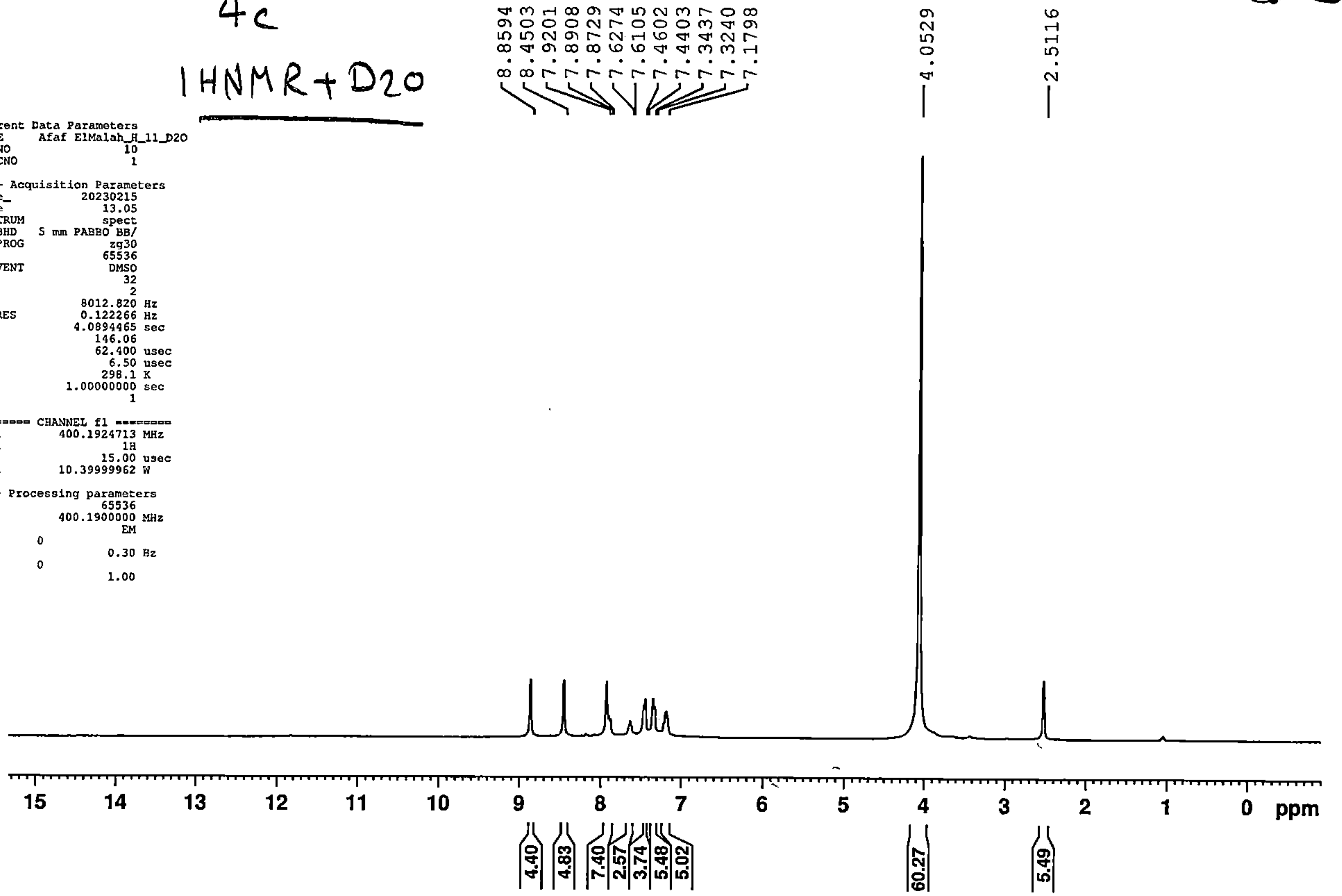

Afaf ElMalah_C_11

Microanalytical Unit - FOPCU - NMR laboratory
www.pharma.cu.edu.eg dir-mau.fopcu@pharma.cu.edu.eg

BRUKER

4c
^{13}C NMR

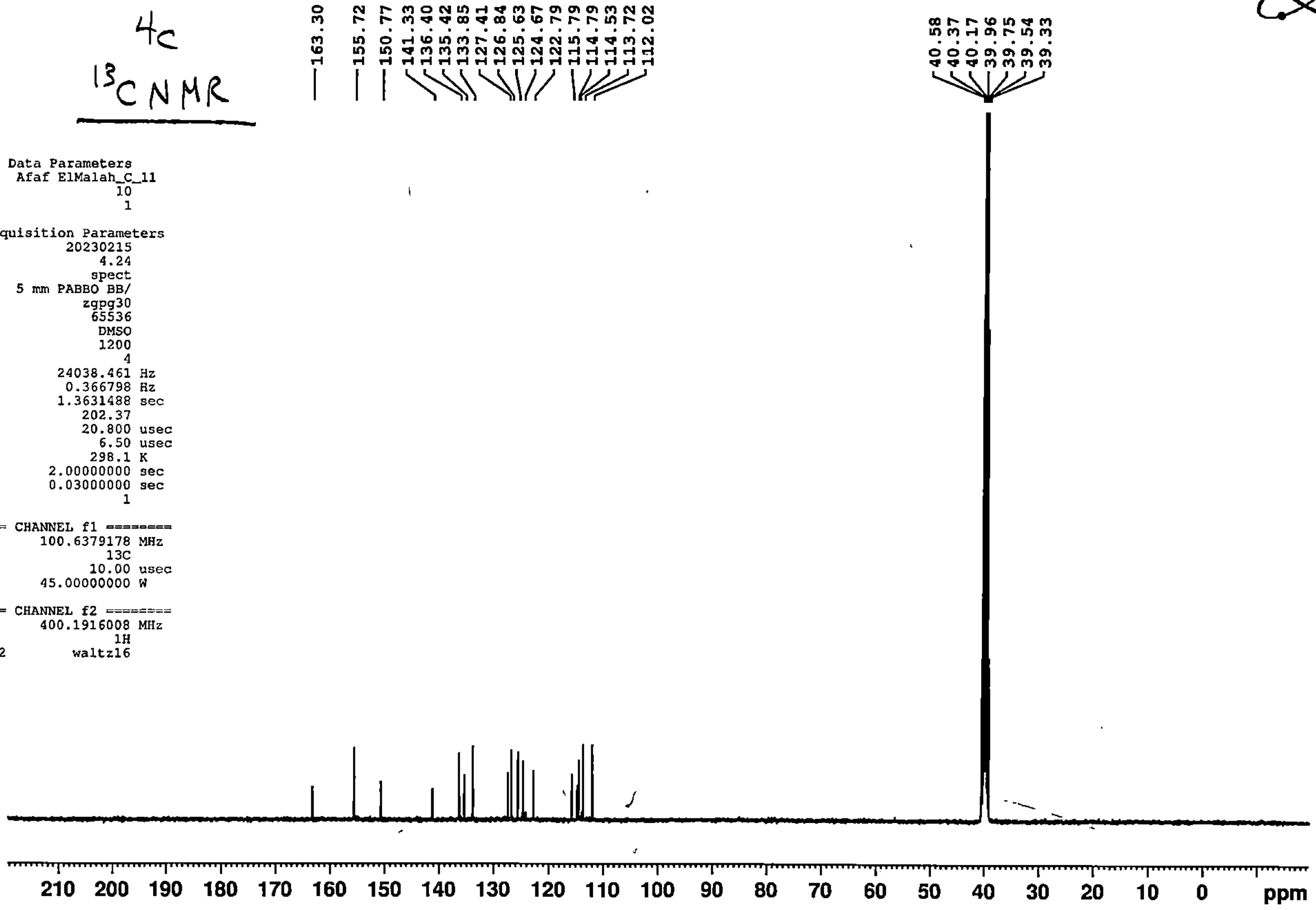


```
Current Data Parameters
NAME       Afaf ElMalah_C_11
EXPNO                     10
PROCNO                     1

F2 - Acquisition Parameters
Date_           20230215
Time                4.24
INSTRUM            spect
PROBHD   5 mm PABBO BB/
PULPROG          zgpg30
TD                65536
SOLVENT            DMSO
NS                 1200
DS                    4
SWH           24038.461 Hz
FIDRES         0.366798 Hz
AQ            1.3631488 sec
RG               202.37
DW               20.800 usec
DE                 6.50 usec
TE                298.1 K
D1           2.00000000 sec
D11          0.03000000 sec
TD0                   1

======== CHANNEL f1 ========
SFO1       100.6379178 MHz
NUC1               13C
P1               10.00 usec
PLW1       45.00000000 W

======== CHANNEL f2 ========
SFO2       400.1916008 MHz
NUC2                1H
CPDPRG[2        waltz16
```

Afaf ElMalah_H_9

Microanalytical Unit - FOPCU - NMR laboratory
www.pharma.cu.edu.eg dir-mau.fopcu@pharma.cu.edu.eg

BRUKER

4d
¹HNMR

```
Current Data Parameters
NAME      Afaf ElMalah_H_9
EXPNO                   10
PROCNO                   1

F2 - Acquisition Parameters
Date_             20230215
Time                  2.00
INSTRUM              spect
PROBHD    5 mm PABBO BB/
PULPROG               zg30
TD                   65536
SOLVENT               DMSO
NS                      32
DS                       2
SWH              8012.820 Hz
FIDRES           0.122266 Hz
AQ              4.0894465 sec
RG                  114.95
DW                  62.400 usec
DE                    6.50 usec
TE                   298.0 K
D1             1.00000000 sec
TD0                      1

======== CHANNEL f1 ========
SFO1          400.1924713 MHz
NUC1                    1H
P1                   15.00 usec
PLW1           10.39999962 W

F2 - Processing parameters
SI                   65536
SF             400.1900000 MHz
WDW                     EM
SSB      0
LB                    0.30 Hz
GB       0
PC                    1.00
```

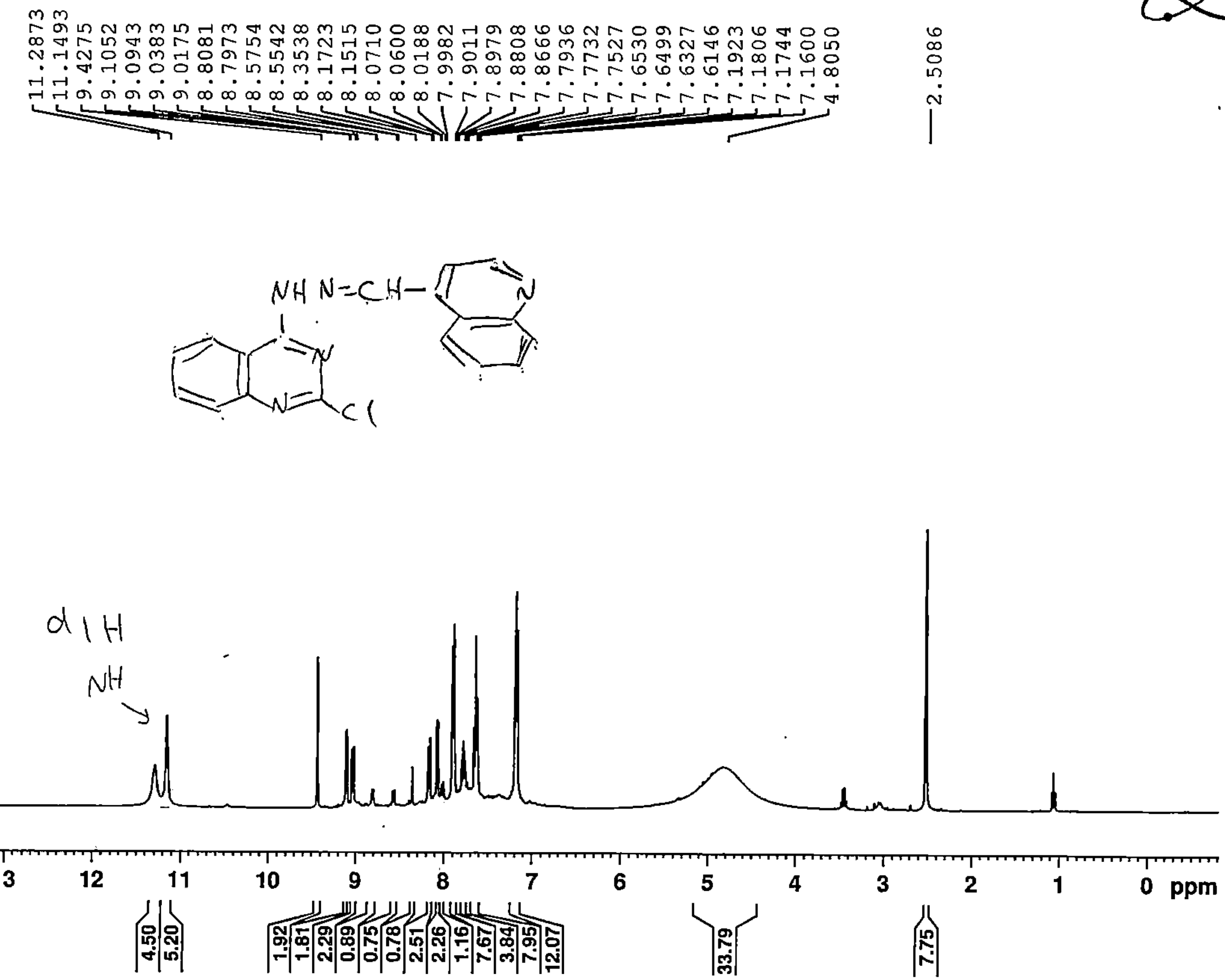

Afaf ElMalah_H_9_D2O

Microanalytical Unit - FOPCU - NMR laboratory
www.pharma.cu.edu.eg dir-mau.fopcu@pharma.cu.edu.eg

BRUKER

4d
^{1}HNMR+D2O

```
Current Data Parameters
NAME      Afaf ElMalah_H_9_D2O
EXPNO               10
PROCNO               1

F2 - Acquisition Parameters
Date_         20230215
Time             12.59
INSTRUM          spect
PROBHD   5 mm PABBO BB/
PULPROG           zg30
TD               65536
SOLVENT           DMSO
NS                  32
DS                   2
SWH           8012.820 Hz
FIDRES        0.122266 Hz
AQ           4.0894465 sec
RG              106.37
DW              62.400 usec
DE                6.50 usec
TE               298.0 K
D1          1.00000000 sec
TD0                  1

======== CHANNEL f1 ========
SFO1       400.1924713 MHz
NUC1                1H
P1               15.00 usec
PLW1       10.39999962 W

F2 - Processing parameters
SI               65536
SF         400.1900000 MHz
WDW                 EM
SSB        0
LB                0.30 Hz
GB         0
PC                1.00
```

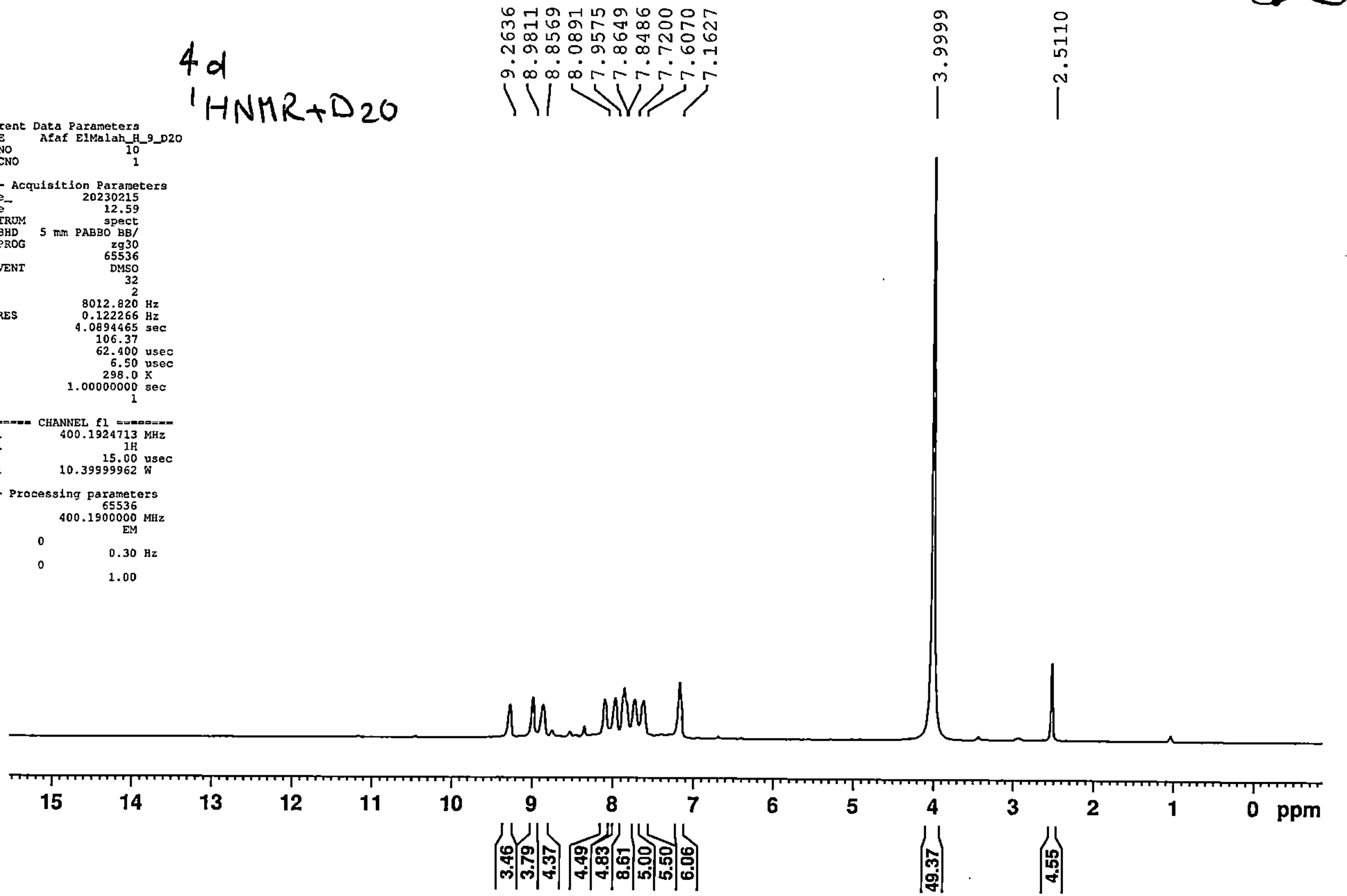

Afaf ElMalah_C_9

Microanalytical Unit - FOPCU - NMR laboratory
www.pharma.cu.edu.eg dir-mau.fopcu@pharma.cu.edu.eg

BRUKER

4d
^{13}C NMR

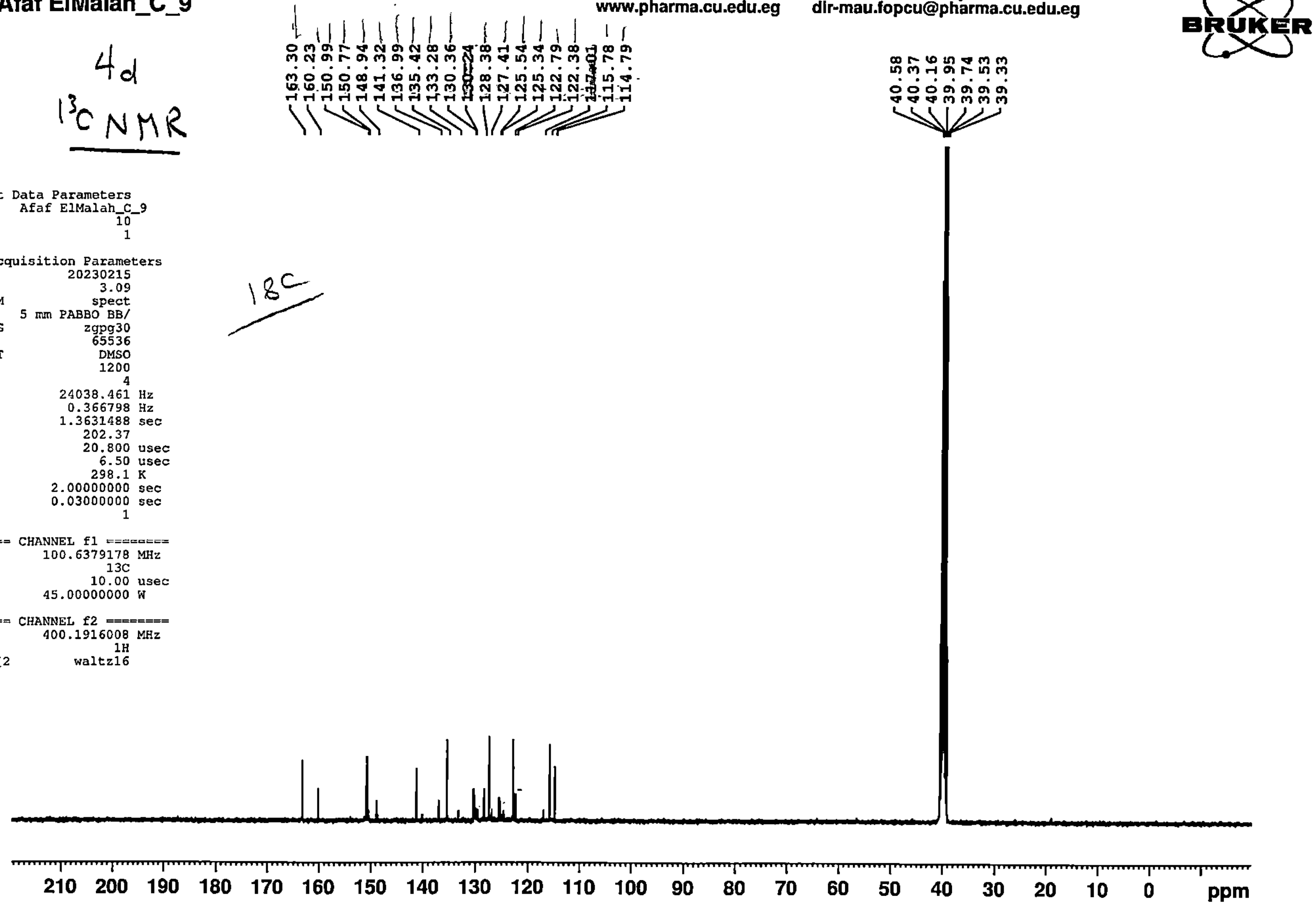

Current Data Parameters
NAME Afaf ElMalah_C_9
EXPNO 10
PROCNO 1

F2 - Acquisition Parameters

Date_	20230215	
Time	3.09	
INSTRUM	spect	
PROBHD	5 mm PABBO BB/	
PULPROG	zgpg30	
TD	65536	
SOLVENT	DMSO	
NS	1200	
DS	4	
SWH	24038.461	Hz
FIDRES	0.366798	Hz
AQ	1.3631488	sec
RG	202.37	
DW	20.800	usec
DE	6.50	usec
TE	298.1	K
D1	2.00000000	sec
D11	0.03000000	sec
TD0	1	

======== CHANNEL f1 ========

SFO1	100.6379178	MHz
NUC1	13C	
P1	10.00	usec
PLW1	45.00000000	W

======== CHANNEL f2 ========

SFO2	400.1916008	MHz
NUC2	1H	
CPDPRG[2	waltz16	

Afaf ElMalah_H_7

Microanalytical Unit - FOPCU - NMR laboratory
www.pharma.cu.edu.eg **dir-mau.fopcu@pharma.cu.edu.eg**

BRUKER

4e
1HNMR

Current Data Parameters
NAME Afaf ElMalah_H_7
EXPNO 10
PROCNO 1

F2 - Acquisition Parameters
Date_ 20230215
Time 0.45
INSTRUM spect
PROBHD 5 mm PABBO BB/
PULPROG zg30
TD 65536
SOLVENT DMSO
NS 32
DS 2
SWH 8012.820 Hz
FIDRES 0.122266 Hz
AQ 4.0894465 sec
RG 114.95
DW 62.400 usec
DE 6.50 usec
TE 298.4 K
D1 1.00000000 sec
TD0 1

======== CHANNEL f1 ========
SFO1 400.1924713 MHz
NUC1 1H
P1 15.00 usec
PLW1 10.39999962 W

F2 - Processing parameters
SI 65536
SF 400.1900000 MHz
WDW EM
SSB 0
LB 0.30 Hz
GB 0
PC 1.00

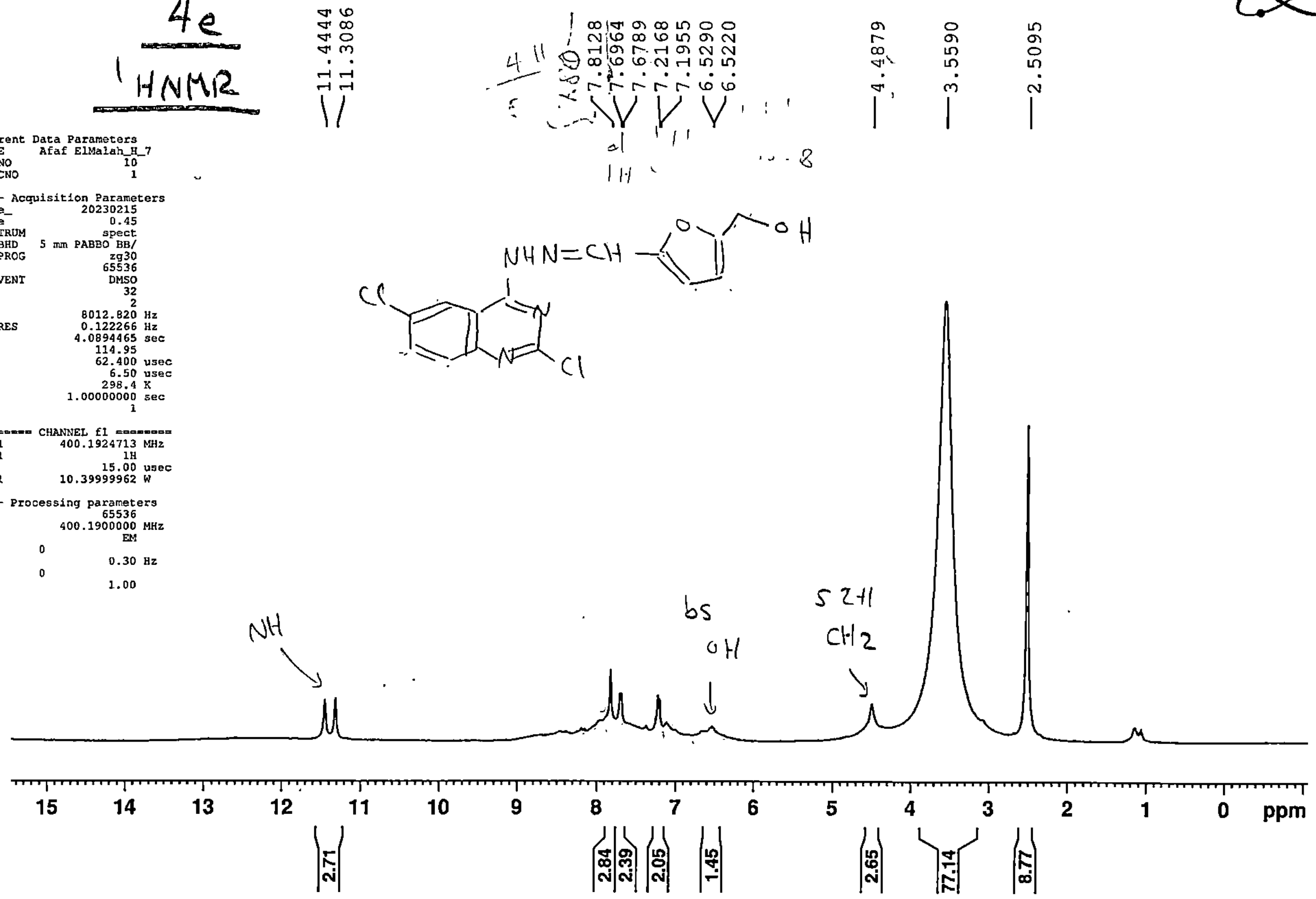

Afaf ElMalah_H_7_D2O

Microanalytical Unit - FOPCU - NMR laboratory
www.pharma.cu.edu.eg dir-mau.fopcu@pharma.cu.edu.eg

BRUKER

4e
^{1}HNMR+D2O

```
Current Data Parameters
NAME     Afaf ElMalah_H_7_D2O
EXPNO                  10
PROCNO                  1

F2 - Acquisition Parameters
Date_            20230215
Time                13.41
INSTRUM             spect
PROBHD   5 mm PABBO BB/
PULPROG              zg30
TD                  65536
SOLVENT              DMSO
NS                     32
DS                      2
SWH              8012.820 Hz
FIDRES           0.122266 Hz
AQ              4.0894465 sec
RG                 114.95
DW                 62.400 usec
DE                   6.50 usec
TE                  298.0 K
D1             1.00000000 sec
TD0                     1

======== CHANNEL f1 ========
SFO1          400.1924713 MHz
NUC1                   1H
P1                  15.00 usec
PLW1          10.39999962 W

F2 - Processing parameters
SI                  65536
SF            400.1900000 MHz
WDW                    EM
SSB       0
LB                   0.30 Hz
GB        0
PC                   1.00
```

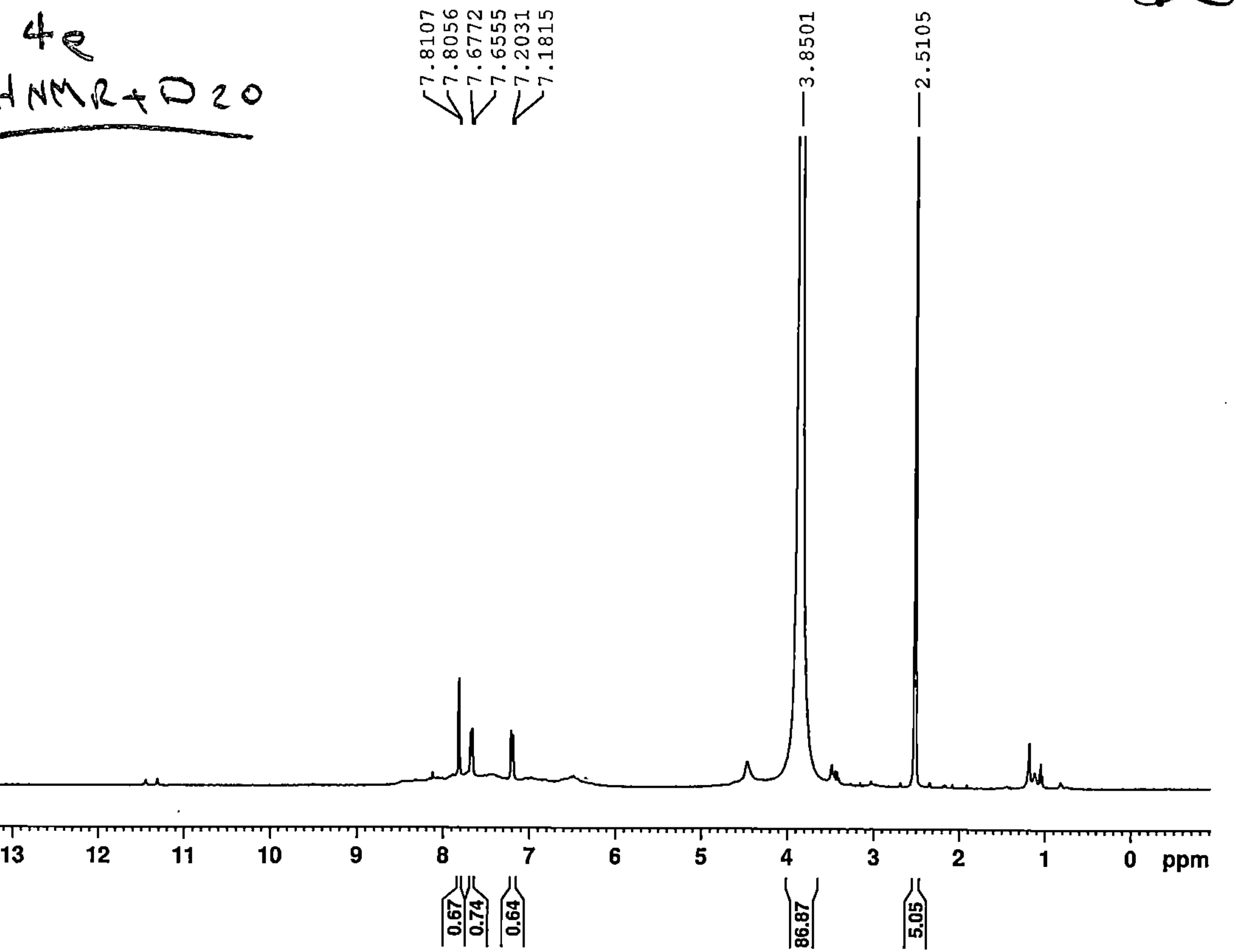

Afaf ElMalah_C_7

Microanalytical Unit - FOPCU - NMR laboratory
www.pharma.cu.edu.eg dir-mau.fopcu@pharma.cu.edu.eg

BRUKER

4e
13C NMR

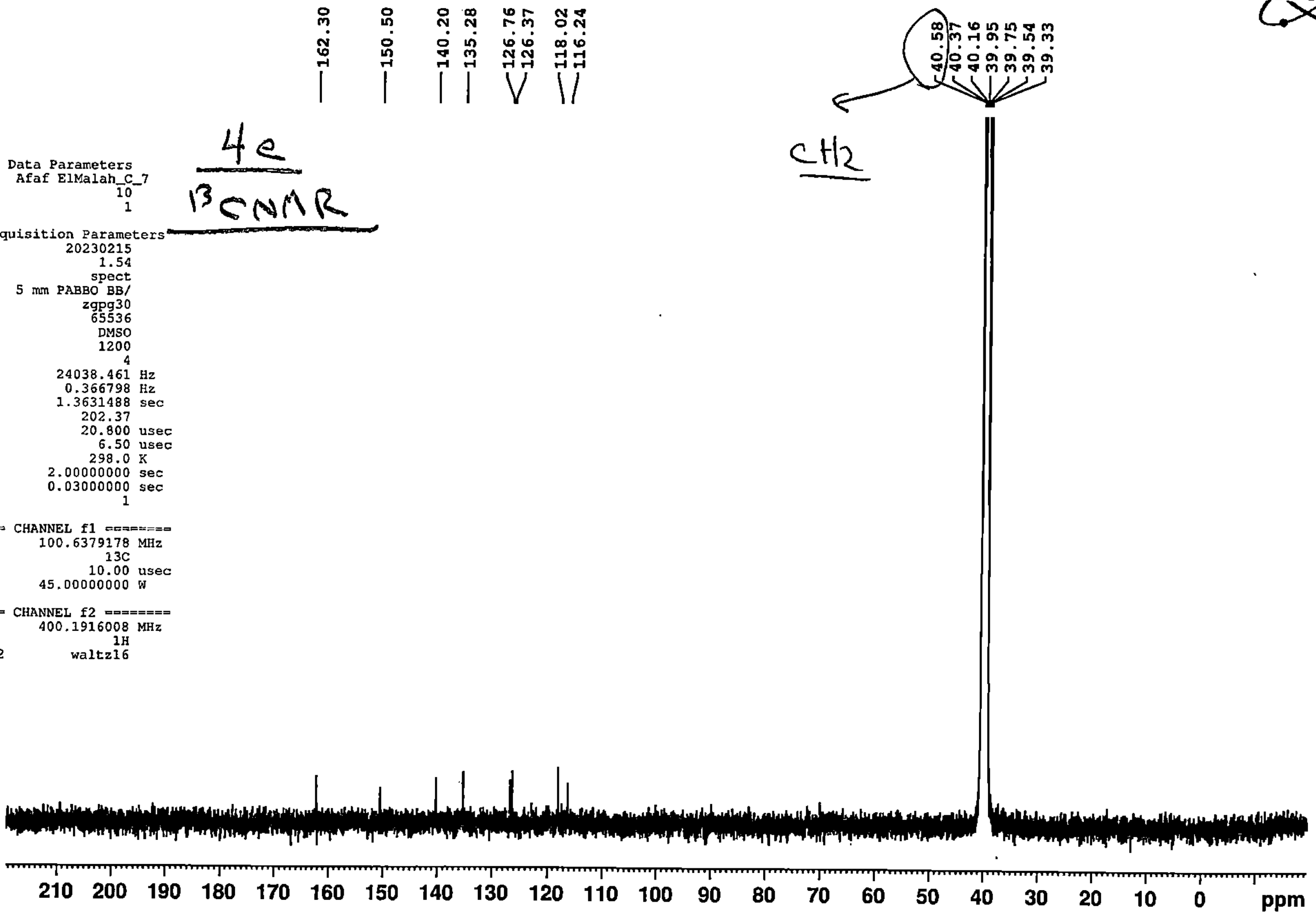


```
Current Data Parameters
NAME       Afaf ElMalah_C_7
EXPNO                    10
PROCNO                    1

F2 - Acquisition Parameters
Date_              20230215
Time                   1.54
INSTRUM               spect
PROBHD     5 mm PABBO BB/
PULPROG             zgpg30
TD                    65536
SOLVENT                DMSO
NS                     1200
DS                        4
SWH              24038.461 Hz
FIDRES            0.366798 Hz
AQ               1.3631488 sec
RG                   202.37
DW                   20.800 usec
DE                     6.50 usec
TE                    298.0 K
D1               2.00000000 sec
D11              0.03000000 sec
TD0                       1

======== CHANNEL f1 ========
SFO1           100.6379178 MHz
NUC1                     13C
P1                     10.00 usec
PLW1             45.00000000 W

======== CHANNEL f2 ========
SFO2           400.1916008 MHz
NUC2                      1H
CPDPRG[2             waltz16
```

Afaf ElMalah_H_5

Microanalytical Unit - FOPCU - NMR laboratory
www.pharma.cu.edu.eg dir-mau.fopcu@pharma.cu.edu.eg

BRUKER

4f

^{1}H NMR

```
Current Data Parameters
NAME     Afaf ElMalah_H_5
EXPNO                  10
PROCNO                  1

F2 - Acquisition Parameters
Date_            20230215
Time                11.28
INSTRUM             spect
PROBHD   5 mm PABBO BB/
PULPROG              zg30
TD                  65536
SOLVENT              DMSO
NS                     32
DS                      2
SWH             8012.820 Hz
FIDRES          0.122266 Hz
AQ             4.0894465 sec
RG                  32.12
DW                 62.400 usec
DE                   6.50 usec
TE                  298.1 K
D1             1.00000000 sec
TD0                     1

======== CHANNEL f1 ========
SFO1        400.1924713 MHz
NUC1                 1H
P1                  15.00 usec
PLW1         10.39999962 W

F2 - Processing parameters
SI                  65536
SF           400.1900000 MHz
WDW                    EM
SSB      0
LB                   0.30 Hz
GB       0
PC                   1.00
```

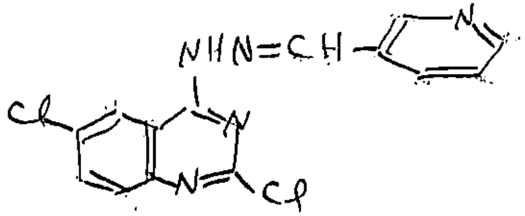

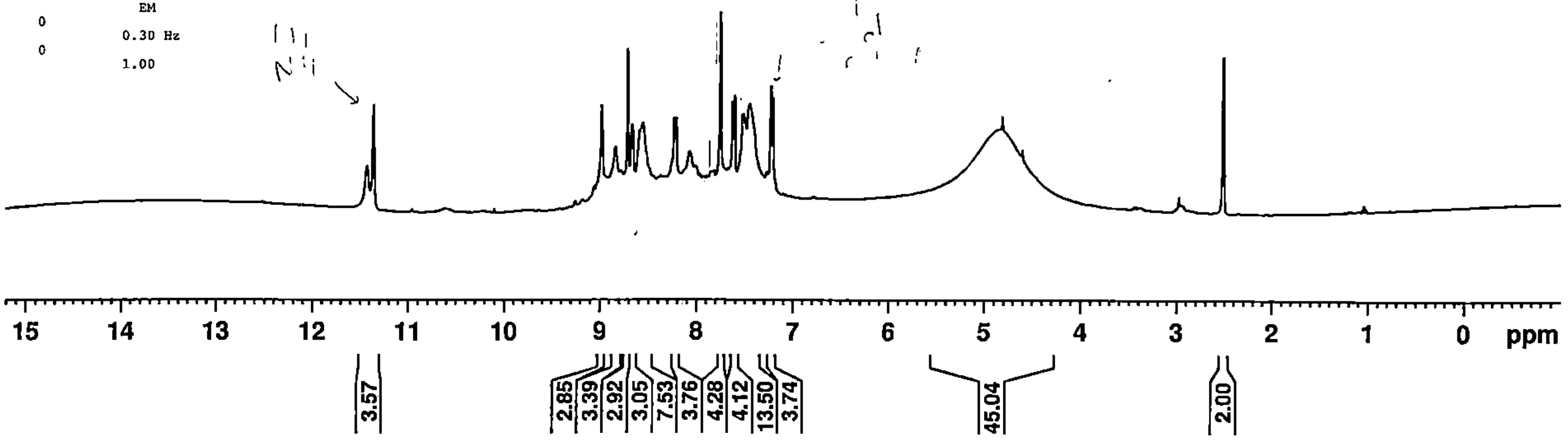

Afaf ElMalah_H_5_D2O

Microanalytical Unit - FOPCU - NMR laboratory
www.pharma.cu.edu.eg dir-mau.fopcu@pharma.cu.edu.eg

BRUKER

4f

^{1}HNMR+D2O

```
Current Data Parameters
NAME     Afaf ElMalah_H_5_D2O
EXPNO              10
PROCNO              1

F2 - Acquisition Parameters
Date_          20230215
Time              13.36
INSTRUM           spect
PROBHD   5 mm PABBO BB/
PULPROG            zg30
TD                65536
SOLVENT            DMSO
NS                   32
DS                    2
SWH           8012.820 Hz
FIDRES        0.122266 Hz
AQ           4.0894465 sec
RG                50.83
DW              62.400 usec
DE                6.50 usec
TE               298.0 K
D1          1.00000000 sec
TD0                   1

======== CHANNEL f1 ========
SFO1       400.1924713 MHz
NUC1                 1H
P1                15.00 usec
PLW1        10.39999962 W

F2 - Processing parameters
SI                65536
SF          400.1900000 MHz
WDW                  EM
SSB       0
LB                 0.30 Hz
GB        0
PC                 1.00
```

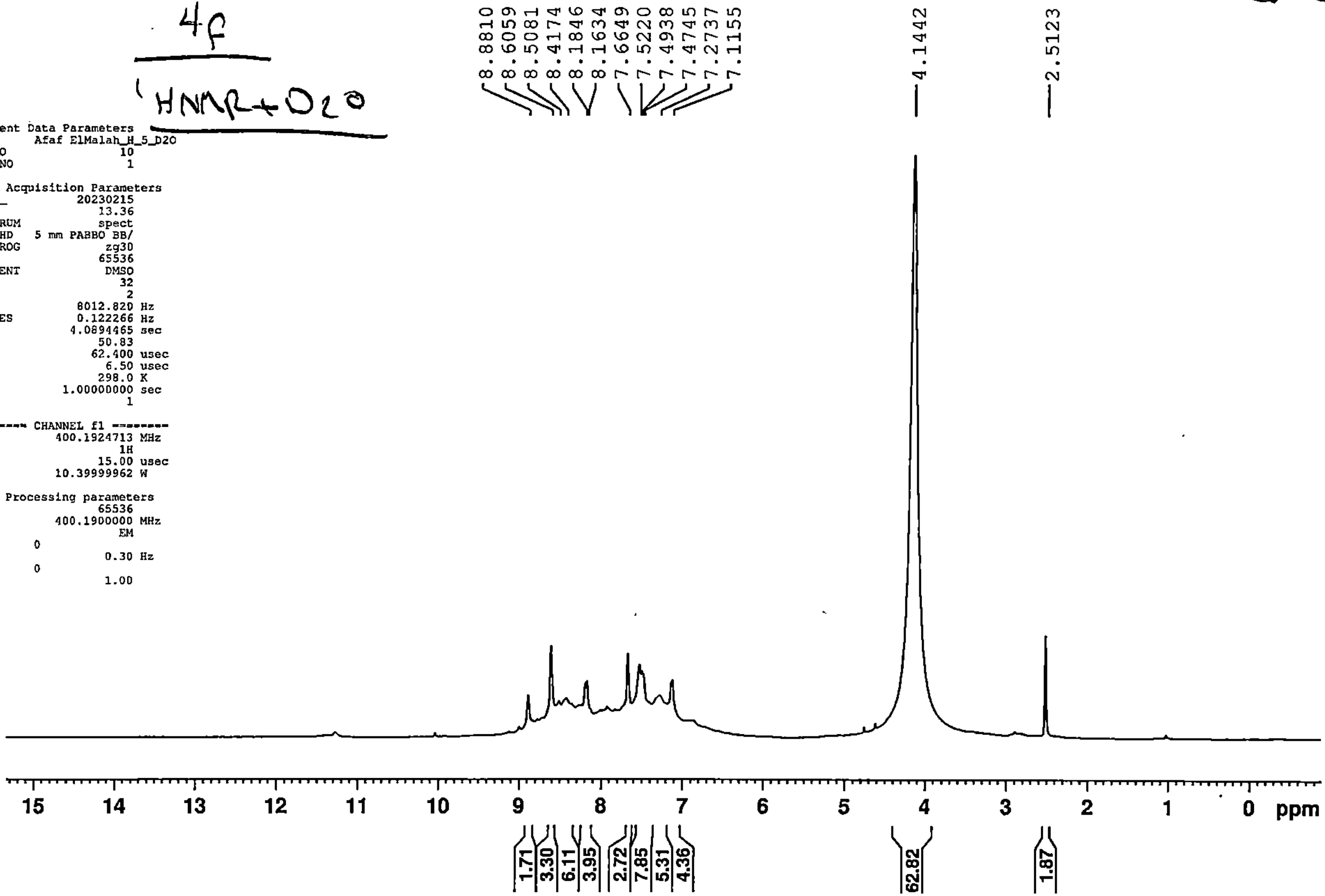

Afaf ElMalah_C_5

Microanalytical Unit - FOPCU - NMR laboratory
www.pharma.cu.edu.eg dir-mau.fopcu@pharma.cu.edu.eg

BRUKER

4e

13C NMR

14

Current Data Parameters
NAME Afaf ElMalah_C_5
EXPNO 10
PROCNO 1

F2 - Acquisition Parameters
Date_ 20230215
Time 11.57
INSTRUM spect
PROBHD 5 mm PABBO BB/
PULPROG zgpg30
TD 65536
SOLVENT DMSO
NS 500
DS 4
SWH 24038.461 Hz
FIDRES 0.366798 Hz
AQ 1.3631488 sec
RG 202.37
DW 20.800 usec
DE 6.50 usec
TE 298.0 K
D1 2.00000000 sec
D11 0.03000000 sec
TD0 1

======== CHANNEL f1 ========
SFO1 100.6379178 MHz
NUC1 13C
P1 10.00 usec
PLW1 45.00000000 W

======== CHANNEL f2 ========
SFO2 400.1916008 MHz
NUC2 1H
CPDPRG[2 waltz16

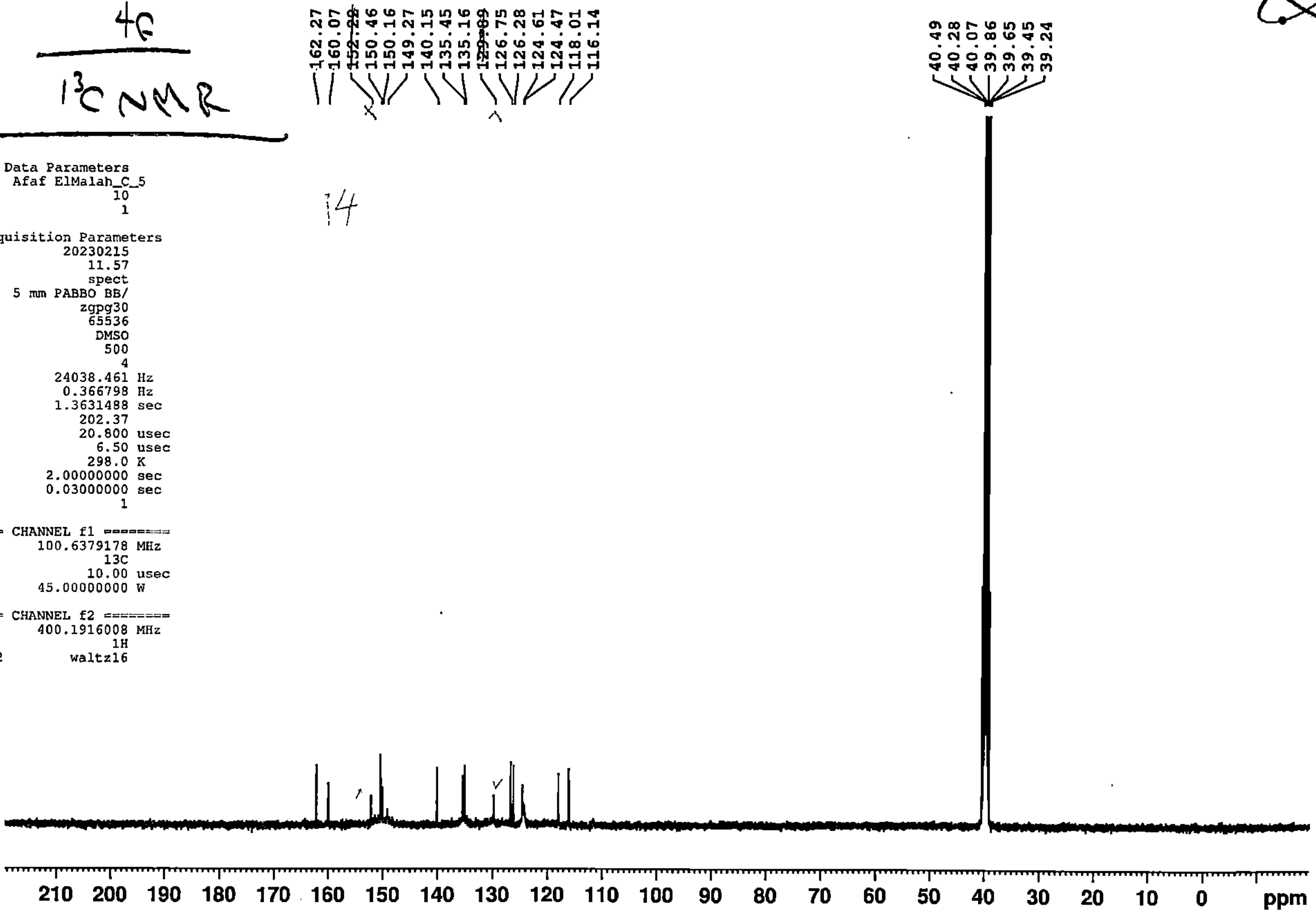

Afaf ElMalah_H_3

Microanalytical Unit - FOPCU - NMR laboratory
www.pharma.cu.edu.eg dir-mau.fopcu@pharma.cu.edu.eg

BRUKER

4g
^{1}HNMR

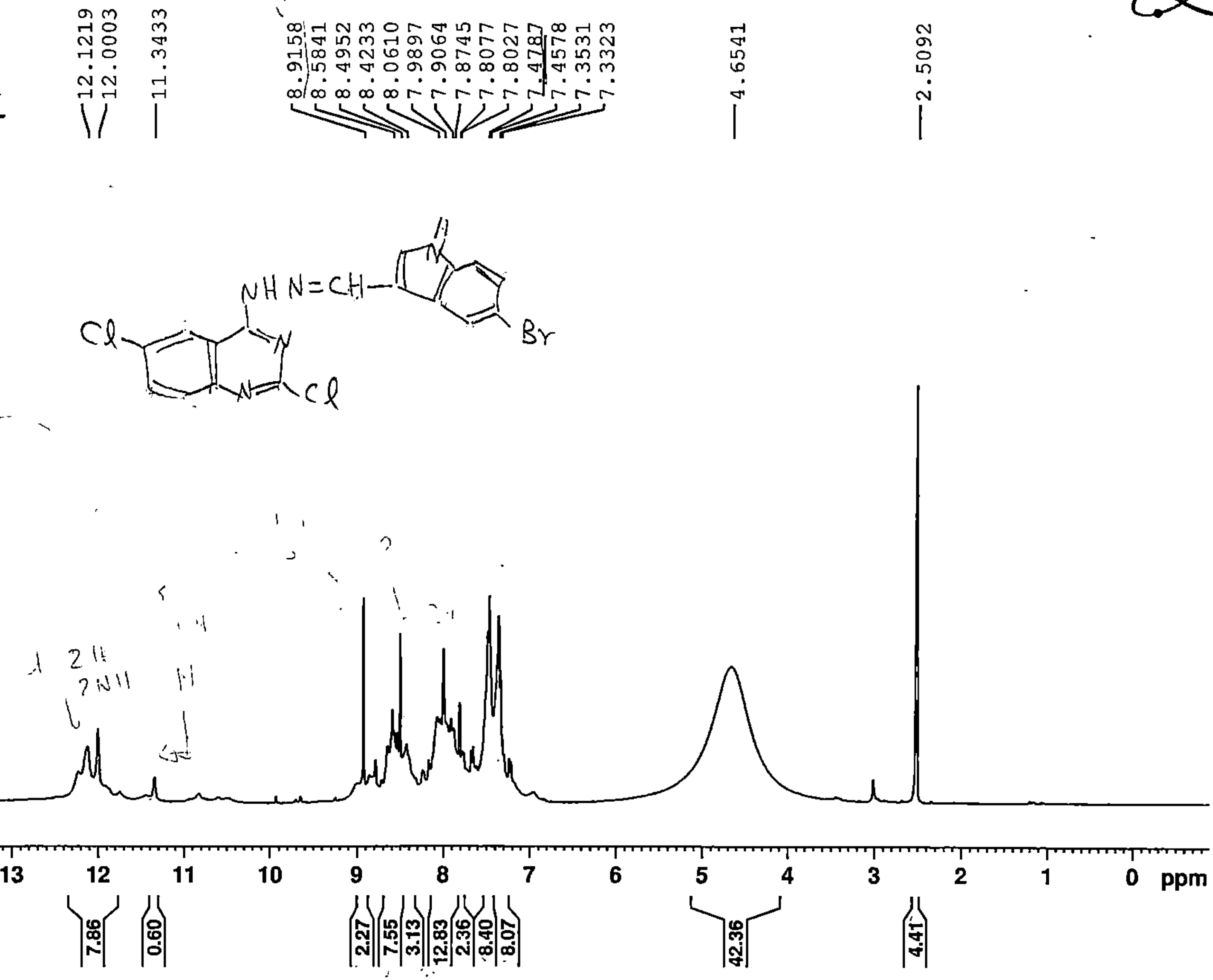


```
Current Data Parameters
NAME      Afaf ElMalah_H_3
EXPNO                  10
PROCNO                  1

F2 - Acquisition Parameters
Date_            20230215
Time                11.23
INSTRUM             spect
PROBHD   5 mm PABBO BB/
PULPROG              zg30
TD                  65536
SOLVENT              DMSO
NS                     32
DS                      2
SWH              8012.820 Hz
FIDRES           0.122266 Hz
AQ              4.0894465 sec
RG                  84.65
DW                 62.400 usec
DE                   6.50 usec
TE                  298.1 K
D1             1.00000000 sec
TD0                     1

======== CHANNEL f1 ========
SFO1          400.1924713 MHz
NUC1                   1H
P1                  15.00 usec
PLW1          10.39999962 W

F2 - Processing parameters
SI                  65536
SF            400.1900000 MHz
WDW                    EM
SSB      0
LB                   0.30 Hz
GB       0
PC                   1.00
```

Afaf ElMalah_H_3_D2O

Microanalytical Unit - FOPCU - NMR laboratory
www.pharma.cu.edu.eg dir-mau.fopcu@pharma.cu.edu.eg

BRUKER

4g
^{1}HNMR+D2O

```
Current Data Parameters
NAME      Afaf ElMalah_H_3_D2O
EXPNO                  10
PROCNO                  1

F2 - Acquisition Parameters
Date_            20230215
Time                13.29
INSTRUM             spect
PROBHD   5 mm PABBO BB/
PULPROG              zg30
TD                  65536
SOLVENT              DMSO
NS                     32
DS                      2
SWH              8012.820 Hz
FIDRES           0.122266 Hz
AQ              4.0894465 sec
RG                 106.37
DW                 62.400 usec
DE                   6.50 usec
TE                  298.0 K
D1             1.00000000 sec
TD0                     1

======== CHANNEL f1 ========
SFO1          400.1924713 MHz
NUC1                   1H
P1                  15.00 usec
PLW1          10.39999962 W

F2 - Processing parameters
SI                  65536
SF            400.1900000 MHz
WDW                    EM
SSB      0
LB                   0.30 Hz
GB       0
PC                   1.00
```

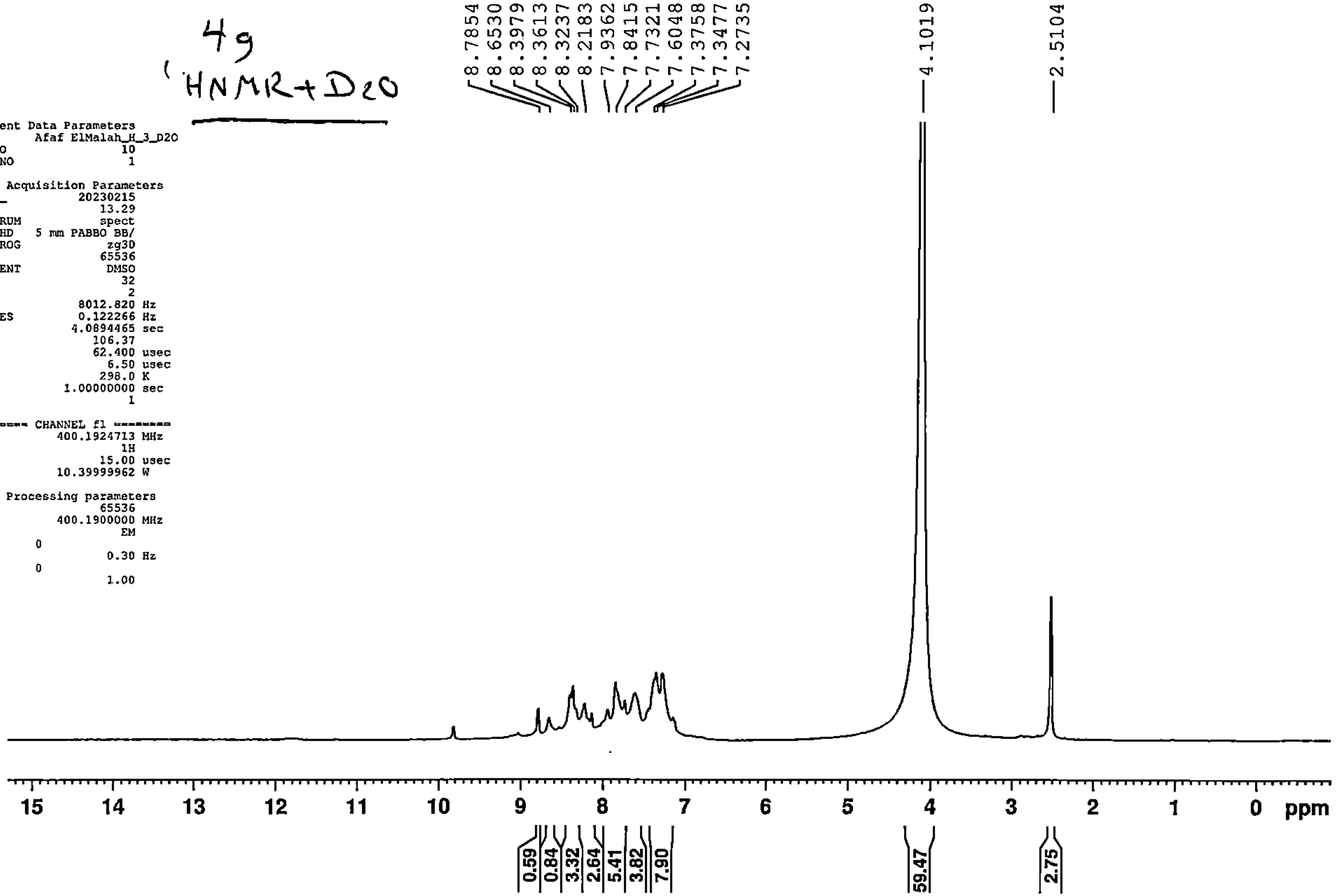

Afaf ElMalah_C_3

Microanalytical Unit - FOPCU - NMR laboratory
www.pharma.cu.edu.eg dir-mau.fopcu@pharma.cu.edu.eg

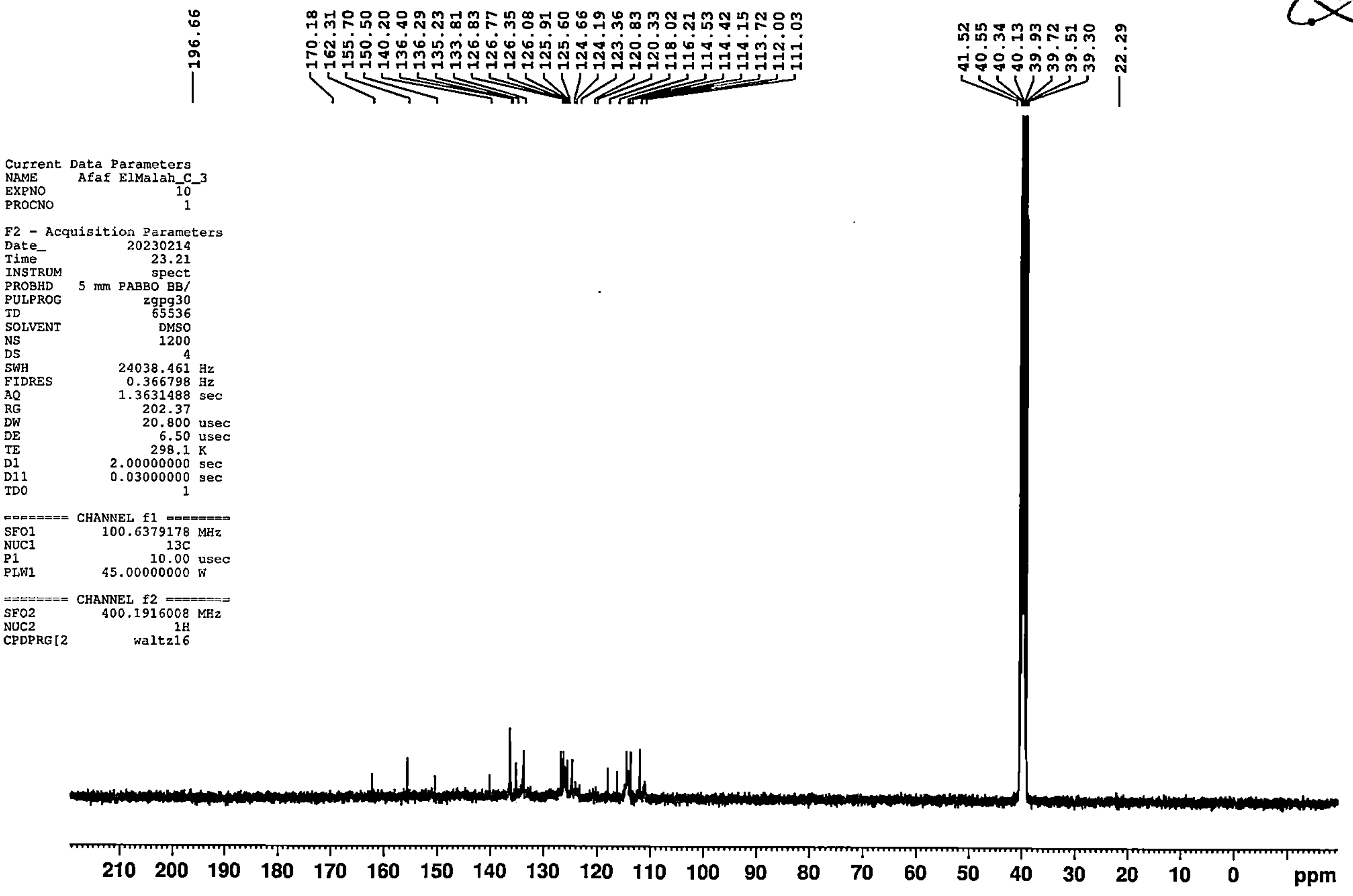


```
Current Data Parameters
NAME        Afaf ElMalah_C_3
EXPNO                     10
PROCNO                     1

F2 - Acquisition Parameters
Date_               20230214
Time                   23.21
INSTRUM                spect
PROBHD    5 mm PABBO BB/
PULPROG              zgpg30
TD                    65536
SOLVENT                DMSO
NS                     1200
DS                        4
SWH               24038.461 Hz
FIDRES             0.366798 Hz
AQ                1.3631488 sec
RG                   202.37
DW                   20.800 usec
DE                     6.50 usec
TE                    298.1 K
D1               2.00000000 sec
D11              0.03000000 sec
TD0                       1

======== CHANNEL f1 ========
SFO1           100.6379178 MHz
NUC1                    13C
P1                    10.00 usec
PLW1            45.00000000 W

======== CHANNEL f2 ========
SFO2           400.1916008 MHz
NUC2                     1H
CPDPRG[2            waltz16
```

Afaf ElMalah_H_1

4h
^{1}HNMR

Microanalytical Unit - FOPCU - NMR laboratory
www.pharma.cu.edu.eg dir-mau.fopcu@pharma.cu.edu.eg

BRUKER

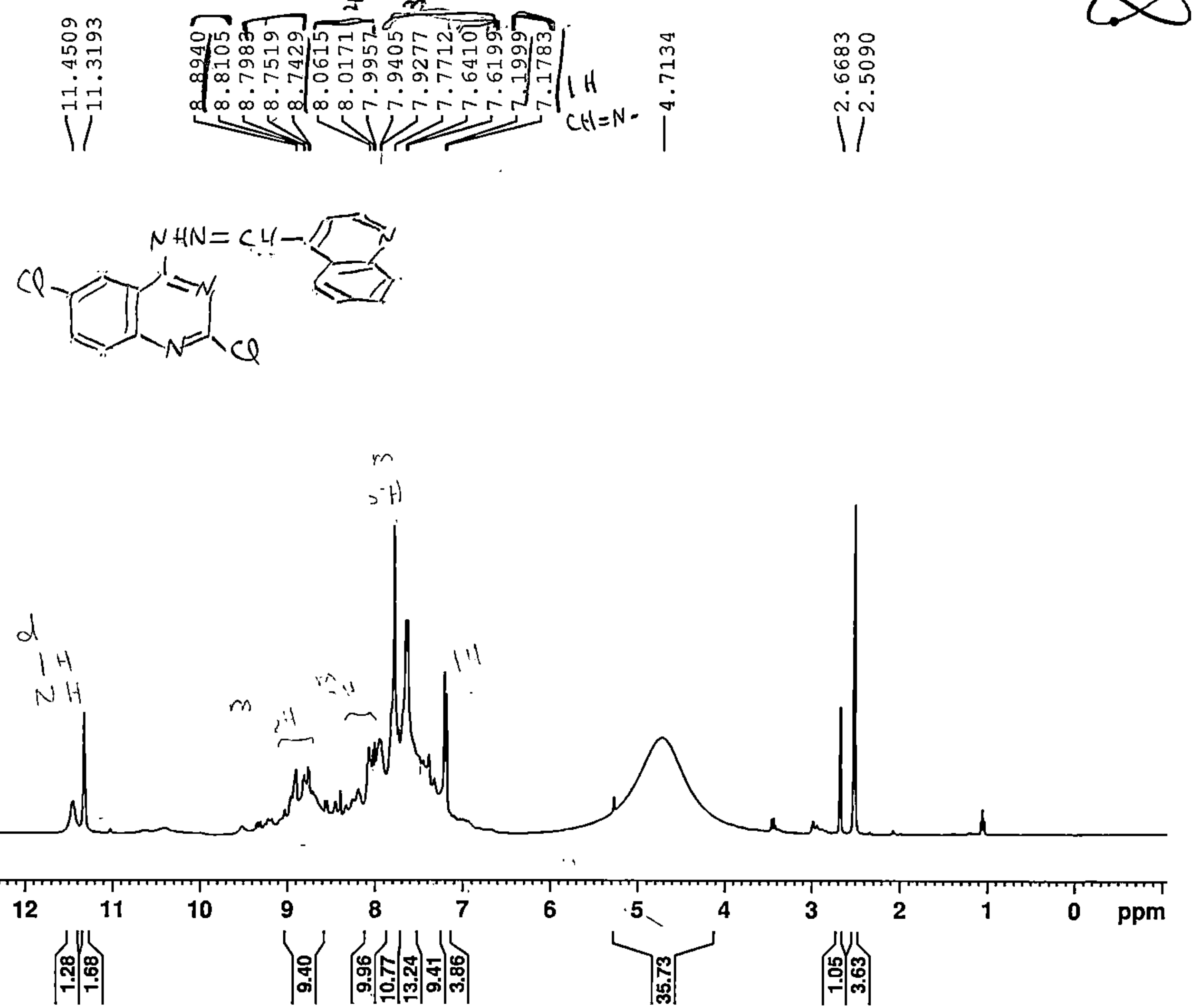


```
Current Data Parameters
NAME        Afaf ElMalah_H_1
EXPNO                     10
PROCNO                     1

F2 - Acquisition Parameters
Date_             20230215
Time                 10.36
INSTRUM              spect
PROBHD   5 mm PABBO BB/
PULPROG               zg30
TD                   65536
SOLVENT               DMSO
NS                      32
DS                       2
SWH            8012.820 Hz
FIDRES         0.122266 Hz
AQ            4.0894465 sec
RG                   64.21
DW             62.400 usec
DE               6.50 usec
TE                298.1 K
D1         1.00000000 sec
TD0                      1

======== CHANNEL f1 ========
SFO1       400.1924713 MHz
NUC1                    1H
P1               15.00 usec
PLW1       10.39999962 W

F2 - Processing parameters
SI                   65536
SF         400.1900000 MHz
WDW                     EM
SSB      0
LB                 0.30 Hz
GB       0
PC                    1.00
```

Afaf ElMalah_H_1_D2O

Microanalytical Unit - FOPCU - NMR laboratory
www.pharma.cu.edu.eg dir-mau.fopcu@pharma.cu.edu.eg

4h
^{1}HNMR+D2O

```
Current Data Parameters
NAME        Afaf ElMalah_H_1_D2O
EXPNO                         10
PROCNO                         1

F2 - Acquisition Parameters
Date_                   20230215
Time                       13.23
INSTRUM                    spect
PROBHD     5 mm PABBO BB/
PULPROG                     zg30
TD                         65536
SOLVENT                     DMSO
NS                            32
DS                             2
SWH                     8012.820 Hz
FIDRES                  0.122266 Hz
AQ                     4.0894465 sec
RG                         84.65
DW                        62.400 usec
DE                          6.50 usec
TE                         298.0 K
D1                    1.00000000 sec
TD0                            1

======== CHANNEL f1 ========
SFO1                 400.1924713 MHz
NUC1                          1H
P1                         15.00 usec
PLW1                 10.39999962 W

F2 - Processing parameters
SI                         65536
SF                   400.1900000 MHz
WDW                           EM
SSB        0
LB                          0.30 Hz
GB         0
PC                          1.00
```

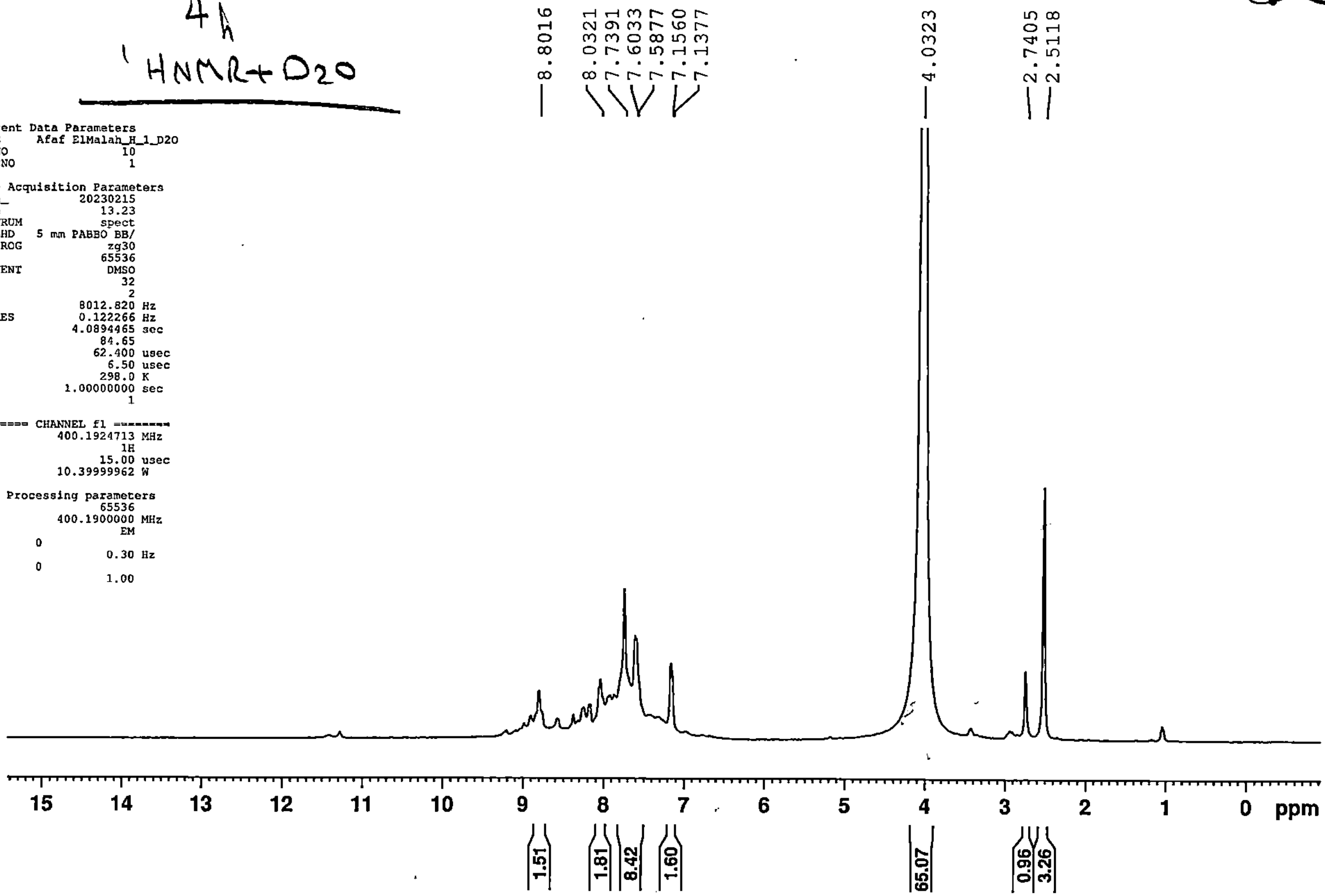

Afaf ElMalah_C_1

Microanalytical Unit - FOPCU - NMR laboratory
www.pharma.cu.edu.eg dir-mau.fopcu@pharma.cu.edu.eg

BRUKER

4h
13C NMR

```
Current Data Parameters
NAME      Afaf ElMalah_C_1
EXPNO                   10
PROCNO                   1

F2 - Acquisition Parameters
Date_             20230215
Time                 11.16
INSTRUM              spect
PROBHD    5 mm PABBO BB/
PULPROG            zgpg30
TD                  65536
SOLVENT              DMSO
NS                    300
DS                      4
SWH             24038.461 Hz
FIDRES           0.366798 Hz
AQ              1.3631488 sec
RG                 202.37
DW                 20.800 usec
DE                   6.50 usec
TE                  298.0 K
D1             2.00000000 sec
D11            0.03000000 sec
TD0                     1

======== CHANNEL f1 ========
SFO1         100.6379178 MHz
NUC1                 13C
P1                  10.00 usec
PLW1          45.00000000 W

======== CHANNEL f2 ========
SFO2         400.1916008 MHz
NUC2                  1H
CPDPRG[2          waltz16
```

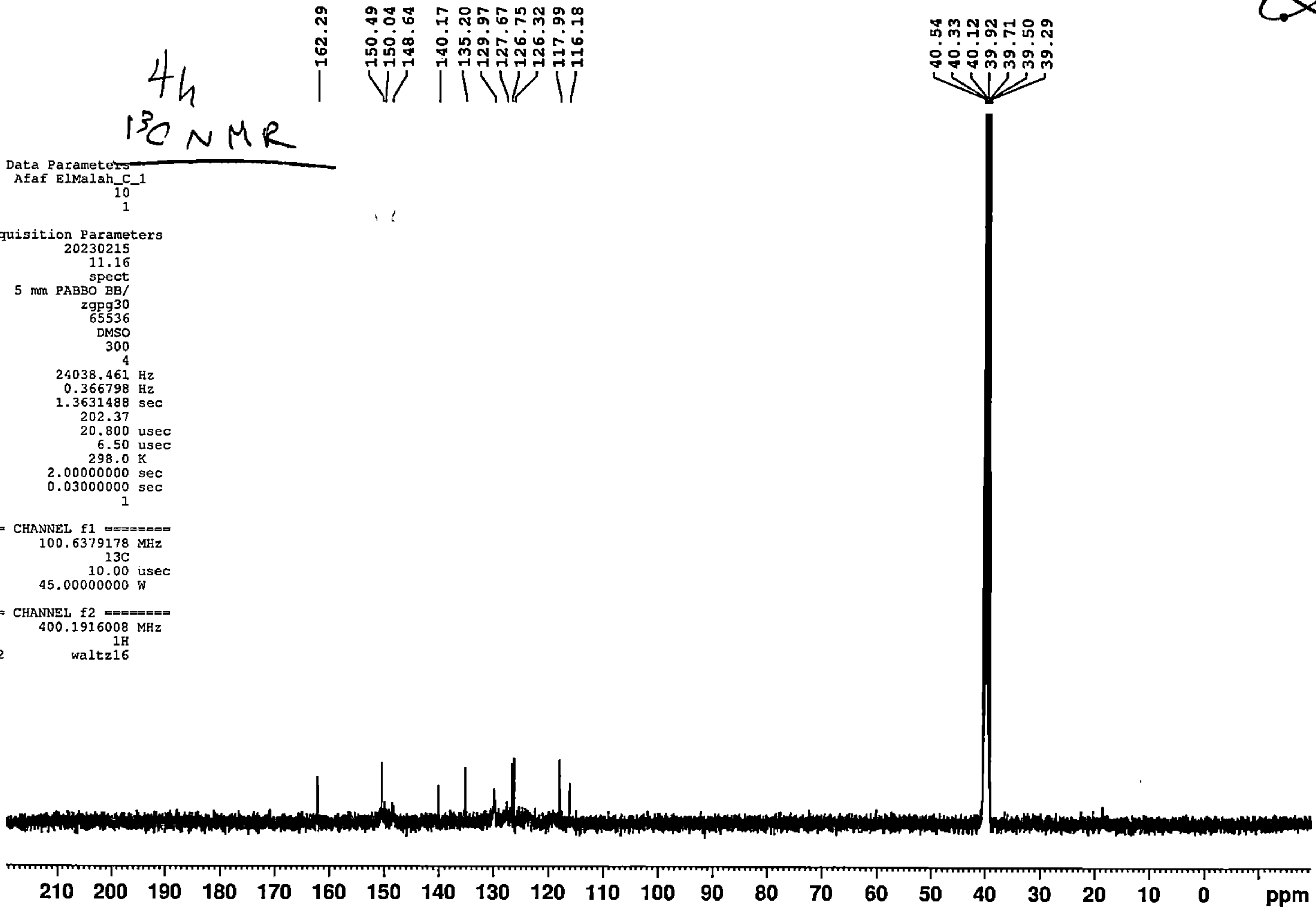

Afaf ElMalah_H_12

Microanalytical Unit - FOPCU - NMR laboratory
www.pharma.cu.edu.eg dir-mau.fopcu@pharma.cu.edu.eg

BRUKER

5c

^{1}H NMR

```
Current Data Parameters
NAME      Afaf ElMalah_H_12
EXPNO                   10
PROCNO                   1

F2 - Acquisition Parameters
Date_             20230216
Time                 12.17
INSTRUM              spect
PROBHD    5 mm PABBO BB/
PULPROG               zg30
TD                   65536
SOLVENT               DMSO
NS                      32
DS                       2
SWH               8012.820 Hz
FIDRES            0.122266 Hz
AQ               4.0894465 sec
RG                  146.06
DW                  62.400 usec
DE                    6.50 usec
TE                   298.0 K
D1              1.00000000 sec
TD0                      1

======== CHANNEL f1 ========
SFO1           400.1924713 MHz
NUC1                    1H
P1                   15.00 usec
PLW1           10.39999962 W

F2 - Processing parameters
SI                   65536
SF             400.1900000 MHz
WDW                     EM
SSB      0
LB                    0.30 Hz
GB       0
PC                    1.00
```

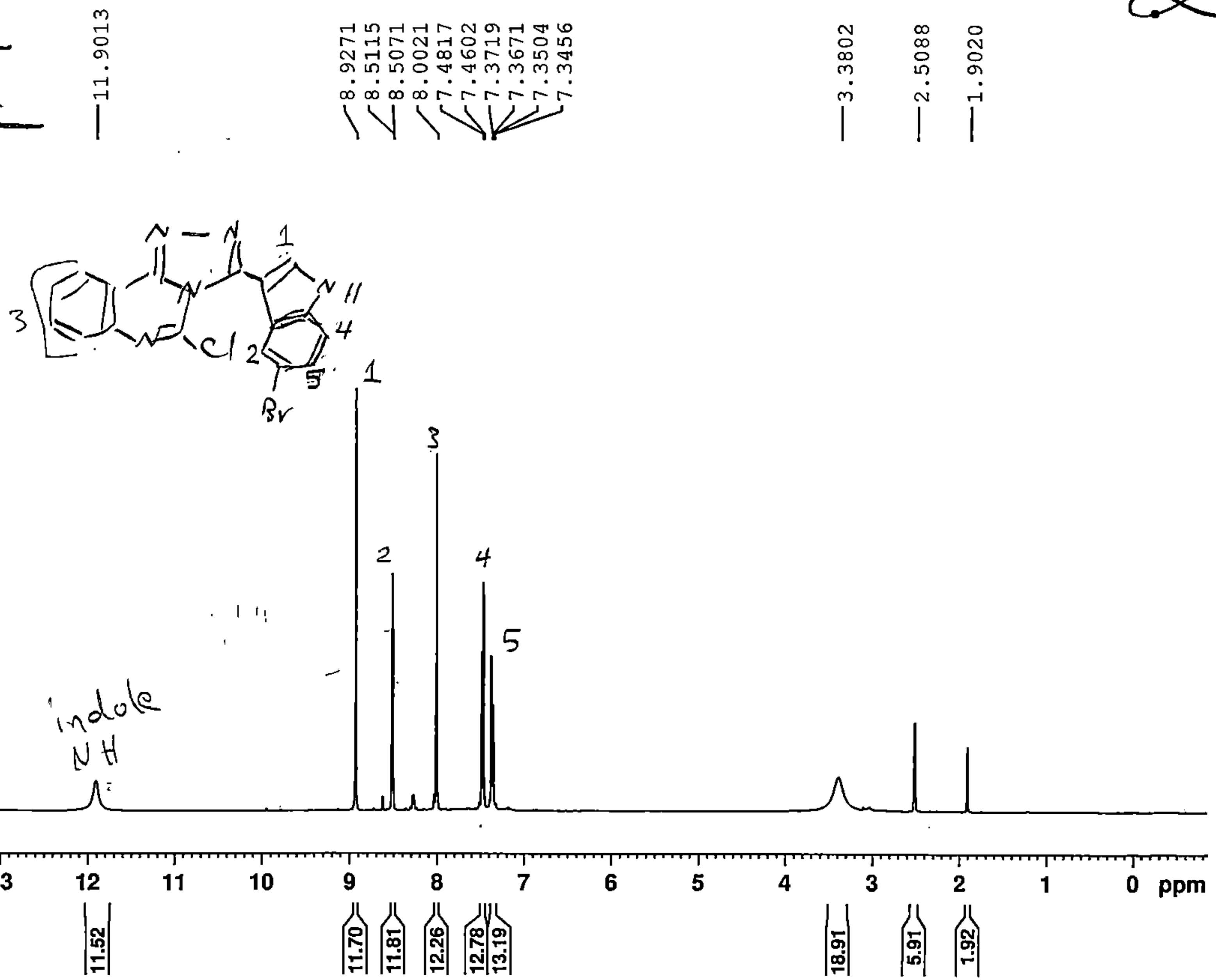

Afaf ElMalah_C_12

5c

^{13}C NMR

Microanalytical Unit - FOPCU - NMR laboratory
www.pharma.cu.edu.eg dir-mau.fopcu@pharma.cu.edu.eg

BRUKER

```
Current Data Parameters
NAME     Afaf ElMalah_C_12
EXPNO                  10
PROCNO                  1

F2 - Acquisition Parameters
Date_            20230216
Time                 7.59
INSTRUM             spect
PROBHD   5 mm PABBO BB/
PULPROG           zgpg30
TD                 65536
SOLVENT             DMSO
NS                  1200
DS                     4
SWH            24038.461 Hz
FIDRES          0.366798 Hz
AQ             1.3631488 sec
RG                202.37
DW                20.800 usec
DE                  6.50 usec
TE                 298.1 K
D1            2.00000000 sec
D11           0.03000000 sec
TD0                    1

======== CHANNEL f1 ========
SFO1        100.6379178 MHz
NUC1                 13C
P1                 10.00 usec
PLW1         45.00000000 W

======== CHANNEL f2 ========
SFO2        400.1916008 MHz
NUC2                  1H
CPDPRG[2         waltz16
```

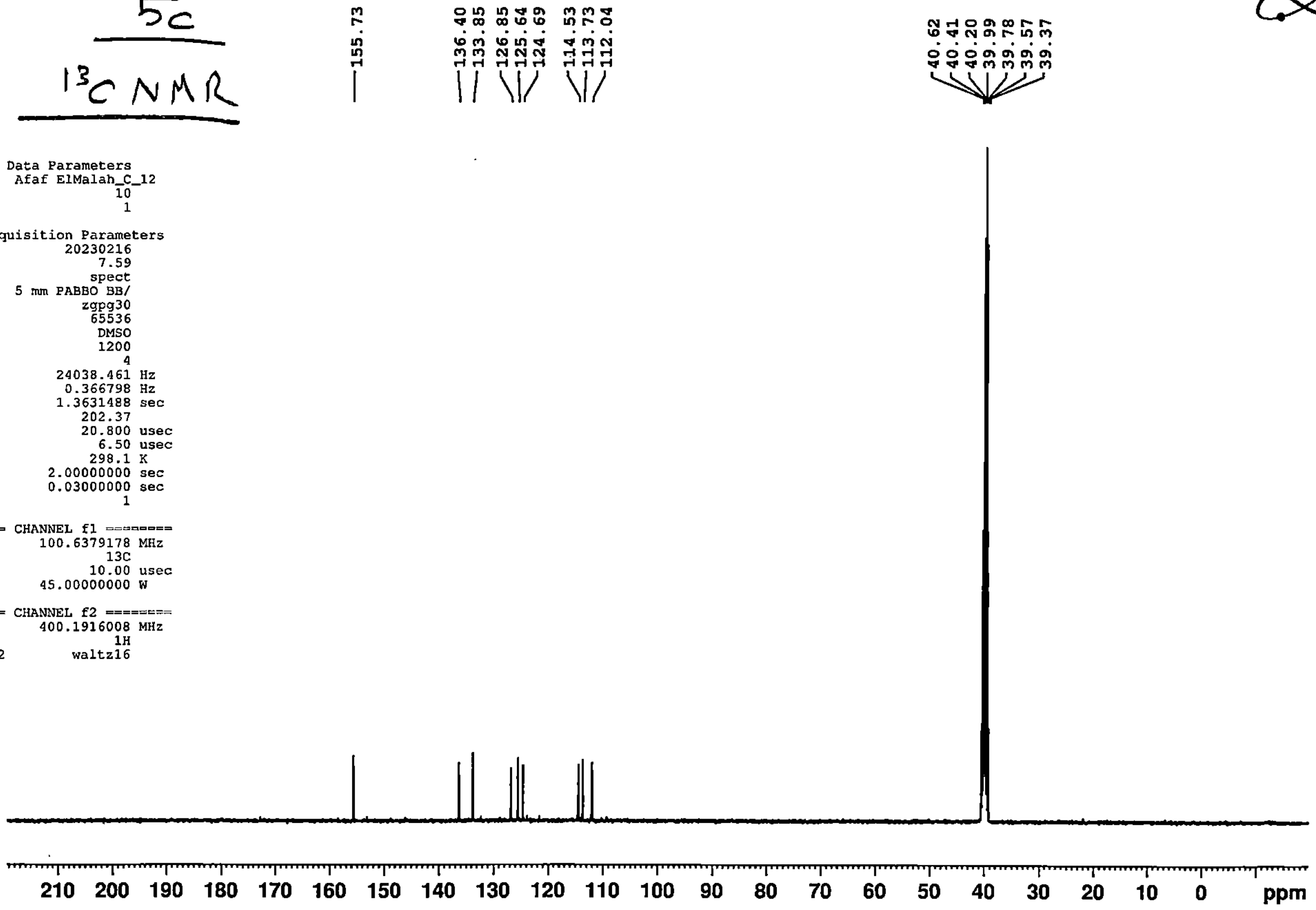

Afaf ElMalah_H_10

Microanalytical Unit - FOPCU - NMR laboratory
www.pharma.cu.edu.eg dir-mau.fopcu@pharma.cu.edu.eg

5d
1HNMR

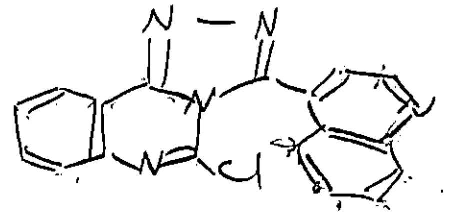

```
Current Data Parameters
NAME       Afaf ElMalah_H_10
EXPNO                     10
PROCNO                     1

F2 - Acquisition Parameters
Date_               20230216
Time                   11.54
INSTRUM                spect
PROBHD   5 mm PABBO BB/
PULPROG                 zg30
TD                     65536
SOLVENT                 DMSO
NS                        32
DS                         2
SWH                 8012.820 Hz
FIDRES              0.122266 Hz
AQ                 4.0894465 sec
RG                    146.06
DW                    62.400 usec
DE                      6.50 usec
TE                     298.0 K
D1                1.00000000 sec
TD0                        1

======== CHANNEL f1 ========
SFO1             400.1924713 MHz
NUC1                      1H
P1                     15.00 usec
PLW1             10.39999962 W

F2 - Processing parameters
SI                     65536
SF               400.1900000 MHz
WDW                       EM
SSB      0
LB                      0.30 Hz
GB       0
PC                      1.00
```

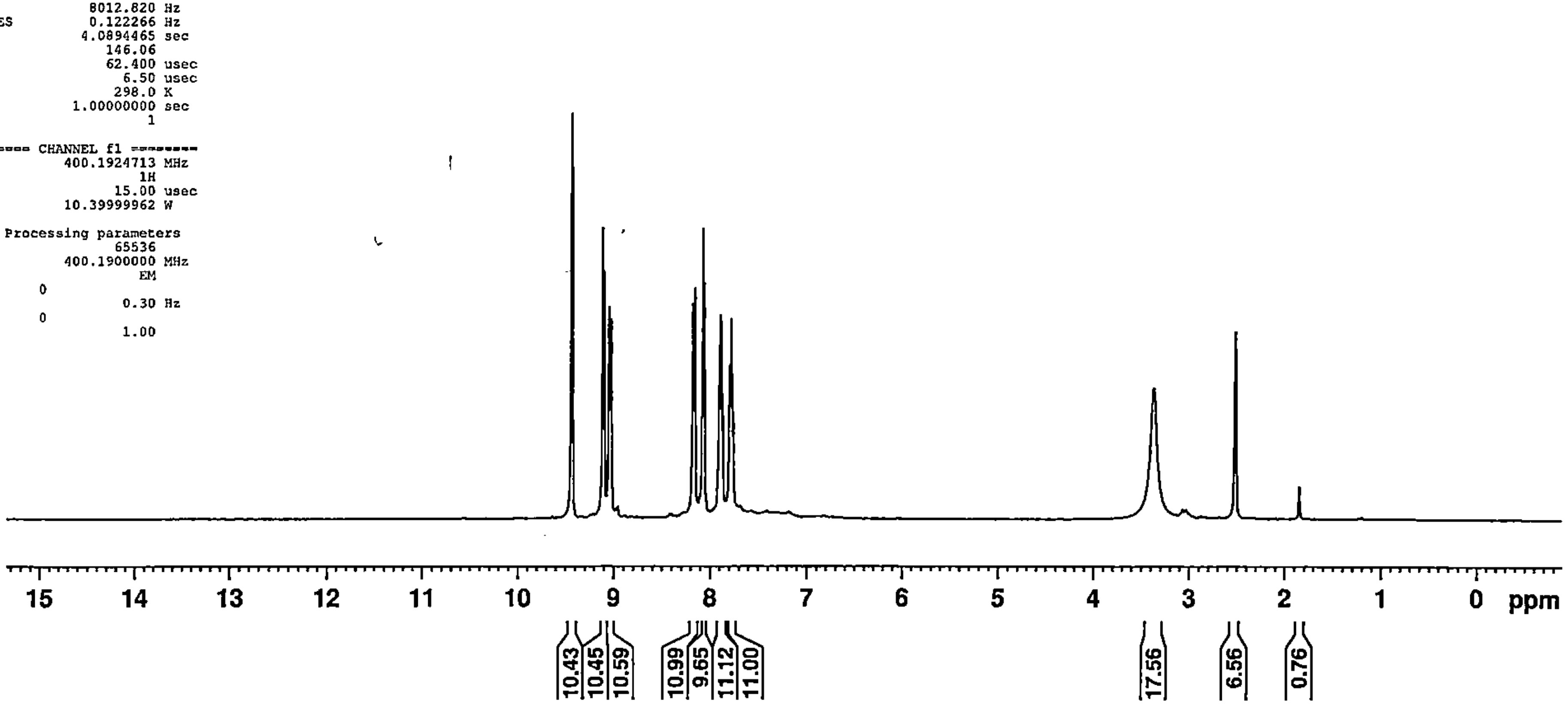

Afaf ElMalah_C_10

Microanalytical Unit - FOPCU - NMR laboratory
www.pharma.cu.edu.eg dir-mau.fopcu@pharma.cu.edu.eg

BRUKER

5d
^{13}C NMR

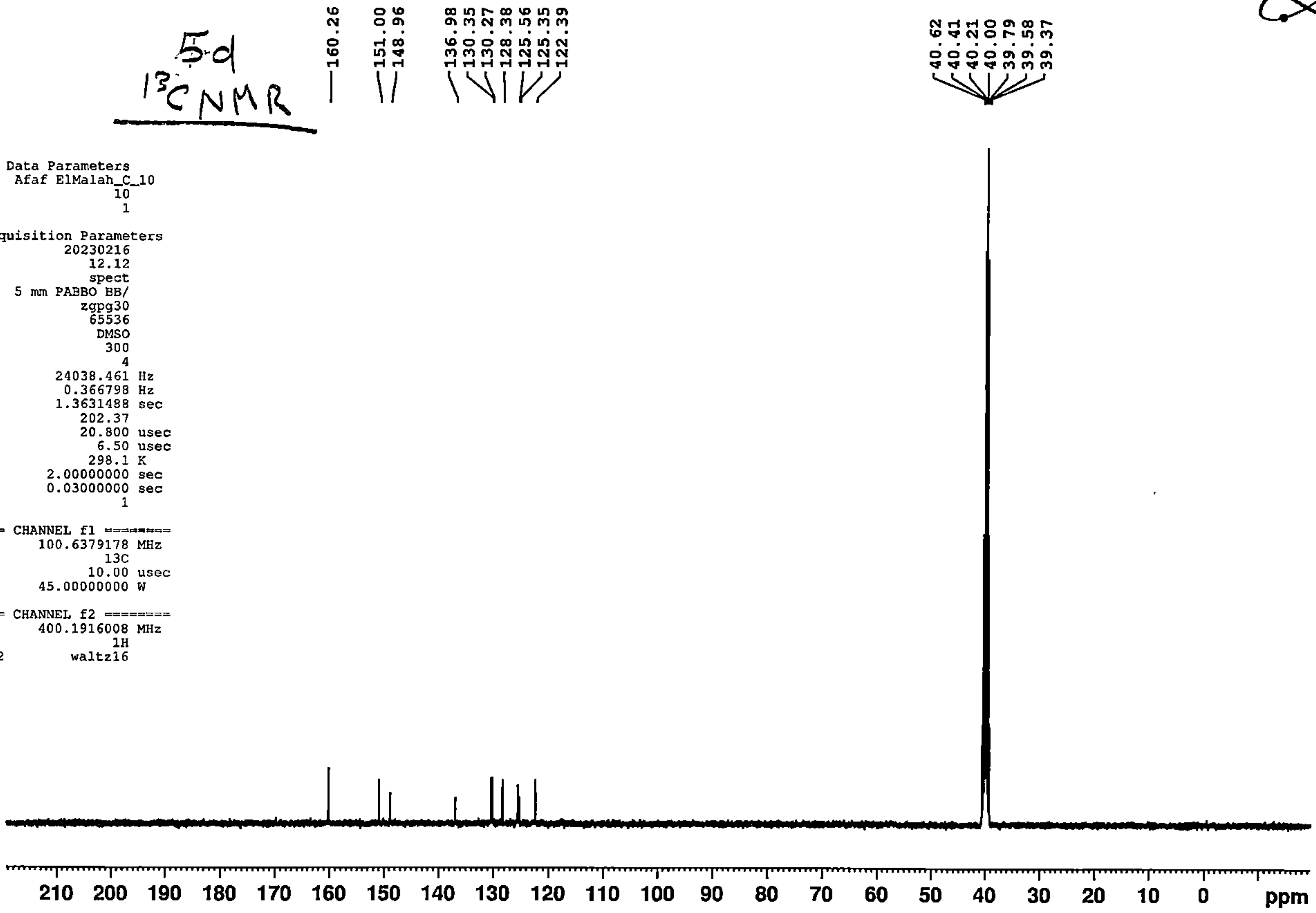


```
Current Data Parameters
NAME      Afaf ElMalah_C_10
EXPNO                  10
PROCNO                  1

F2 - Acquisition Parameters
Date_            20230216
Time                12.12
INSTRUM             spect
PROBHD    5 mm PABBO BB/
PULPROG           zgpg30
TD                 65536
SOLVENT             DMSO
NS                   300
DS                     4
SWH            24038.461 Hz
FIDRES          0.366798 Hz
AQ             1.3631488 sec
RG                202.37
DW                20.800 usec
DE                  6.50 usec
TE                 298.1 K
D1            2.00000000 sec
D11           0.03000000 sec
TD0                    1

======== CHANNEL f1 ========
SFO1        100.6379178 MHz
NUC1                 13C
P1                 10.00 usec
PLW1         45.00000000 W

======== CHANNEL f2 ========
SFO2        400.1916008 MHz
NUC2                  1H
CPDPRG[2          waltz16
```

Afaf ElMalah_H_8

Microanalytical Unit - FOPCU - NMR laboratory
www.pharma.cu.edu.eg dir-mau.fopcu@pharma.cu.edu.eg

BRUKER

5e

^{1}HNMR

```
Current Data Parameters
NAME      Afaf ElMalah_H_8
EXPNO                   10
PROCNO                   1

F2 - Acquisition Parameters
Date_             20230216
Time                  4.16
INSTRUM              spect
PROBHD   5 mm PABBO BB/
PULPROG               zg30
TD                   65536
SOLVENT               DMSO
NS                      32
DS                       2
SWH             8012.820 Hz
FIDRES          0.122266 Hz
AQ             4.0894465 sec
RG                  202.37
DW                  62.400 usec
DE                    6.50 usec
TE                   298.1 K
D1            1.00000000 sec
TD0                      1

======== CHANNEL f1 ========
SFO1         400.1924713 MHz
NUC1                    1H
P1                   15.00 usec
PLW1         10.39999962 W

F2 - Processing parameters
SI                   65536
SF           400.1900000 MHz
WDW                     EM
SSB       0
LB                    0.30 Hz
GB        0
PC                    1.00
```

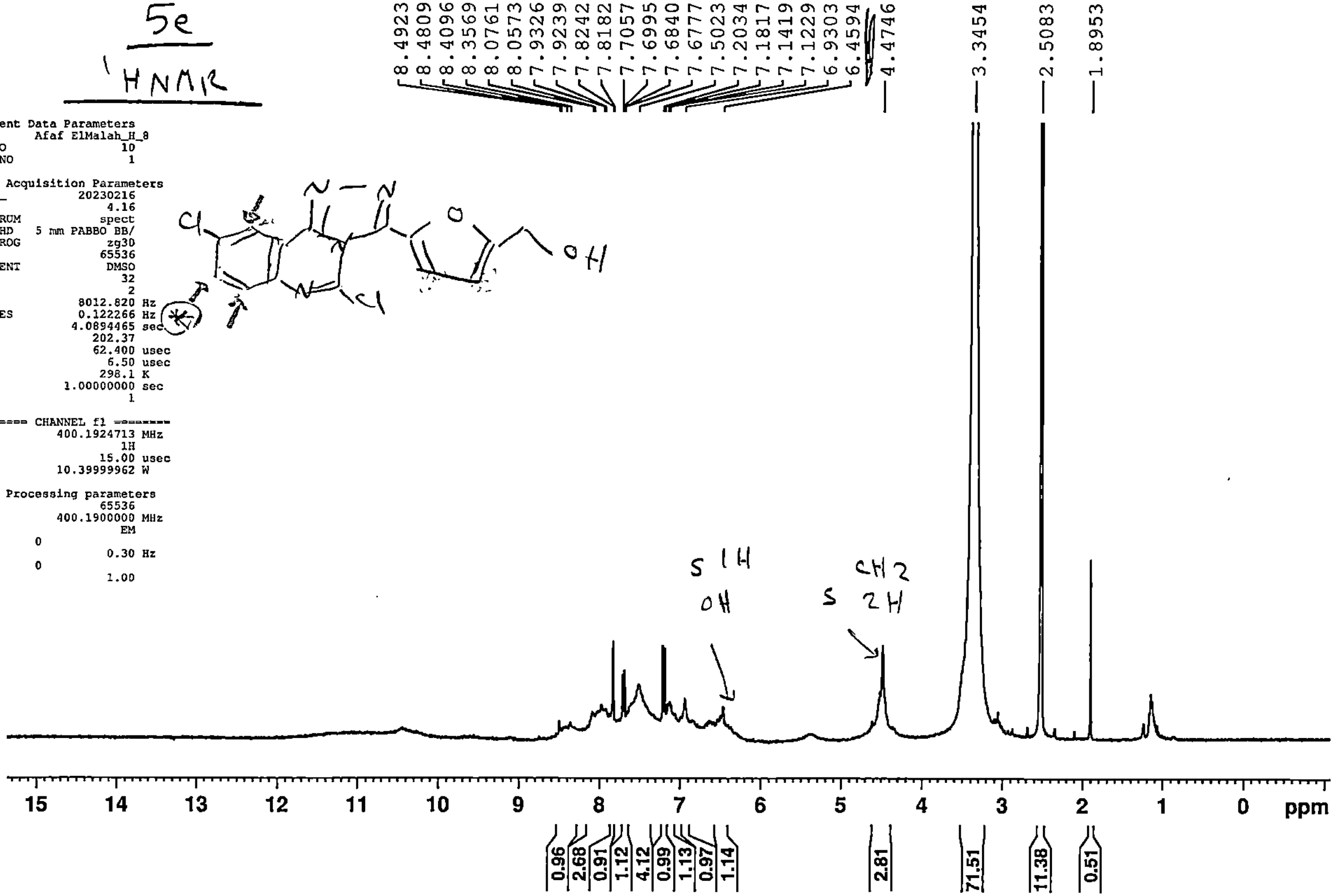

Afaf ElMalah_H_6

Microanalytical Unit - FOPCU - NMR laboratory
www.pharma.cu.edu.eg dir-mau.fopcu@pharma.cu.edu.eg

5f

1HNMR

Current Data Parameters
NAME Afaf ElMalah_H_6
EXPNO 10
PROCNO 1

F2 - Acquisition Parameters
Date_ 20230216
Time 11.29
INSTRUM spect
PROBHD 5 mm PABBO BB/
PULPROG zg30
TD 65536
SOLVENT DMSO
NS 32
DS 2
SWH 8012.820 Hz
FIDRES 0.122266 Hz
AQ 4.0894465 sec
RG 72.66
DW 62.400 usec
DE 6.50 usec
TE 298.0 K
D1 1.00000000 sec
TD0 1

======== CHANNEL f1 ========
SFO1 400.1924713 MHz
NUC1 1H
P1 15.00 usec
PLW1 10.39999962 W

F2 - Processing parameters
SI 65536
SF 400.1900000 MHz
WDW EM
SSB 0
LB 0.30 Hz
GB 0
PC 1.00

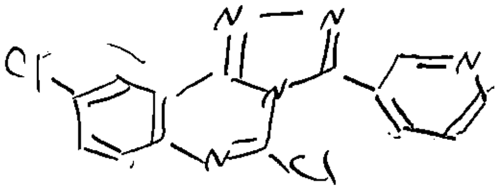

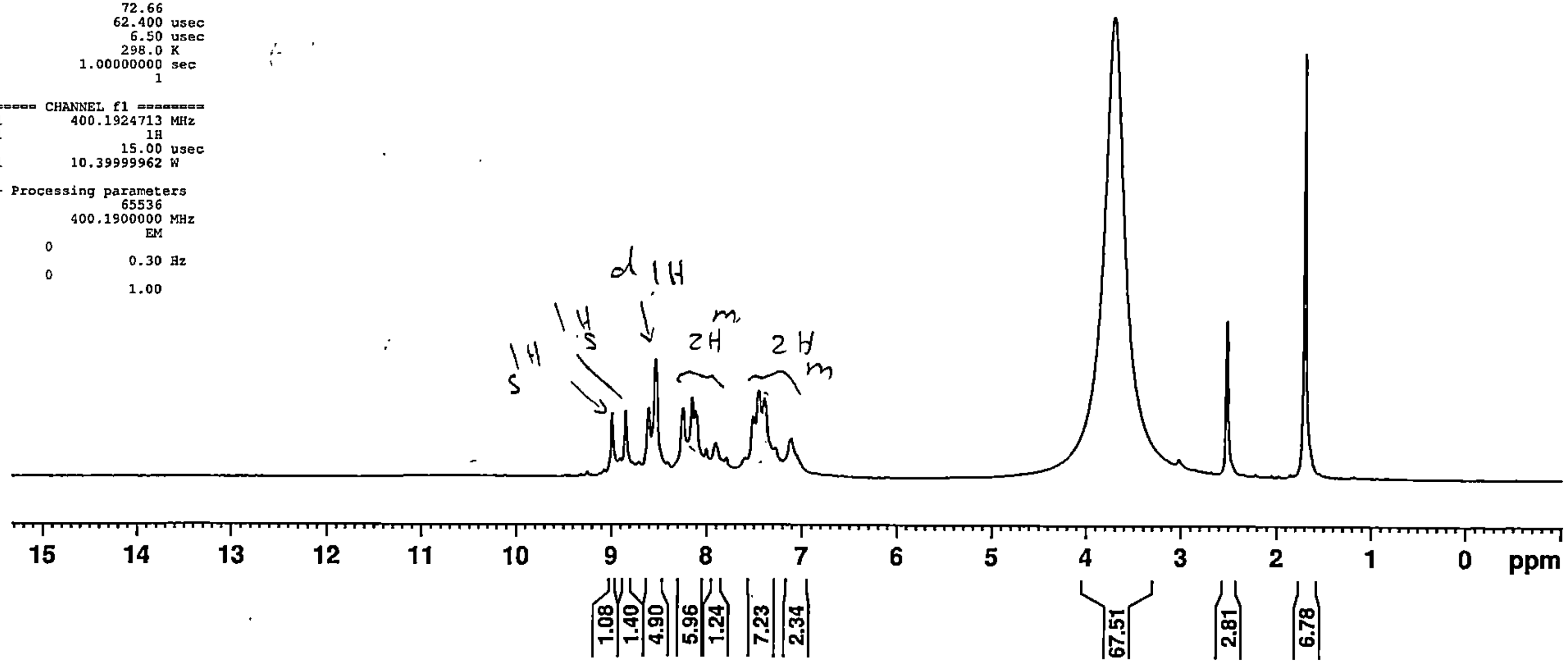

Afaf ElMalah_H_4

Microanalytical Unit - FOPCU - NMR laboratory
www.pharma.cu.edu.eg dir-mau.fopcu@pharma.cu.edu.eg

5g
1HNMR

```
Current Data Parameters
NAME      Afaf ElMalah_H_4
EXPNO                   10
PROCNO                   1

F2 - Acquisition Parameters
Date_             20230216
Time                  1.46
INSTRUM              spect
PROBHD   5 mm PABBO BB/
PULPROG               zg30
TD                   65536
SOLVENT               DMSO
NS                      32
DS                       2
SWH            8012.820 Hz
FIDRES         0.122266 Hz
AQ            4.0894465 sec
RG                  114.95
DW               62.400 usec
DE                 6.50 usec
TE                 298.1 K
D1          1.00000000 sec
TD0                      1

======== CHANNEL f1 ========
SFO1        400.1924713 MHz
NUC1                    1H
P1                15.00 usec
PLW1        10.39999962 W

F2 - Processing parameters
SI                   65536
SF          400.1900000 MHz
WDW                     EM
SSB       0
LB                 0.30 Hz
GB        0
PC                    1.00
```

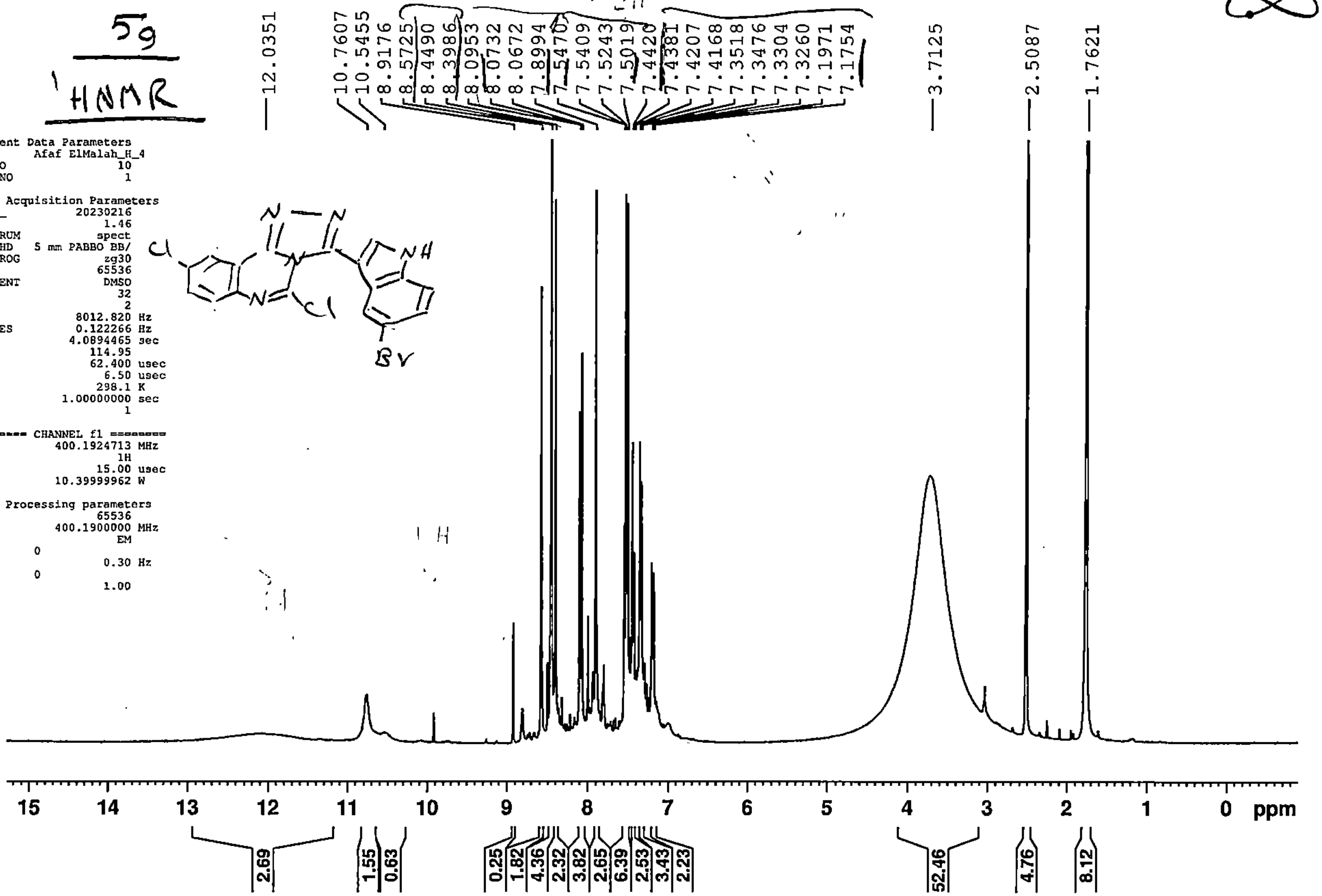

Afaf ElMalah_C_4

Microanalytical Unit - FOPCU - NMR laboratory
www.pharma.cu.edu.eg dir-mau.fopcu@pharma.cu.edu.eg

BRUKER

5g

13CNMR

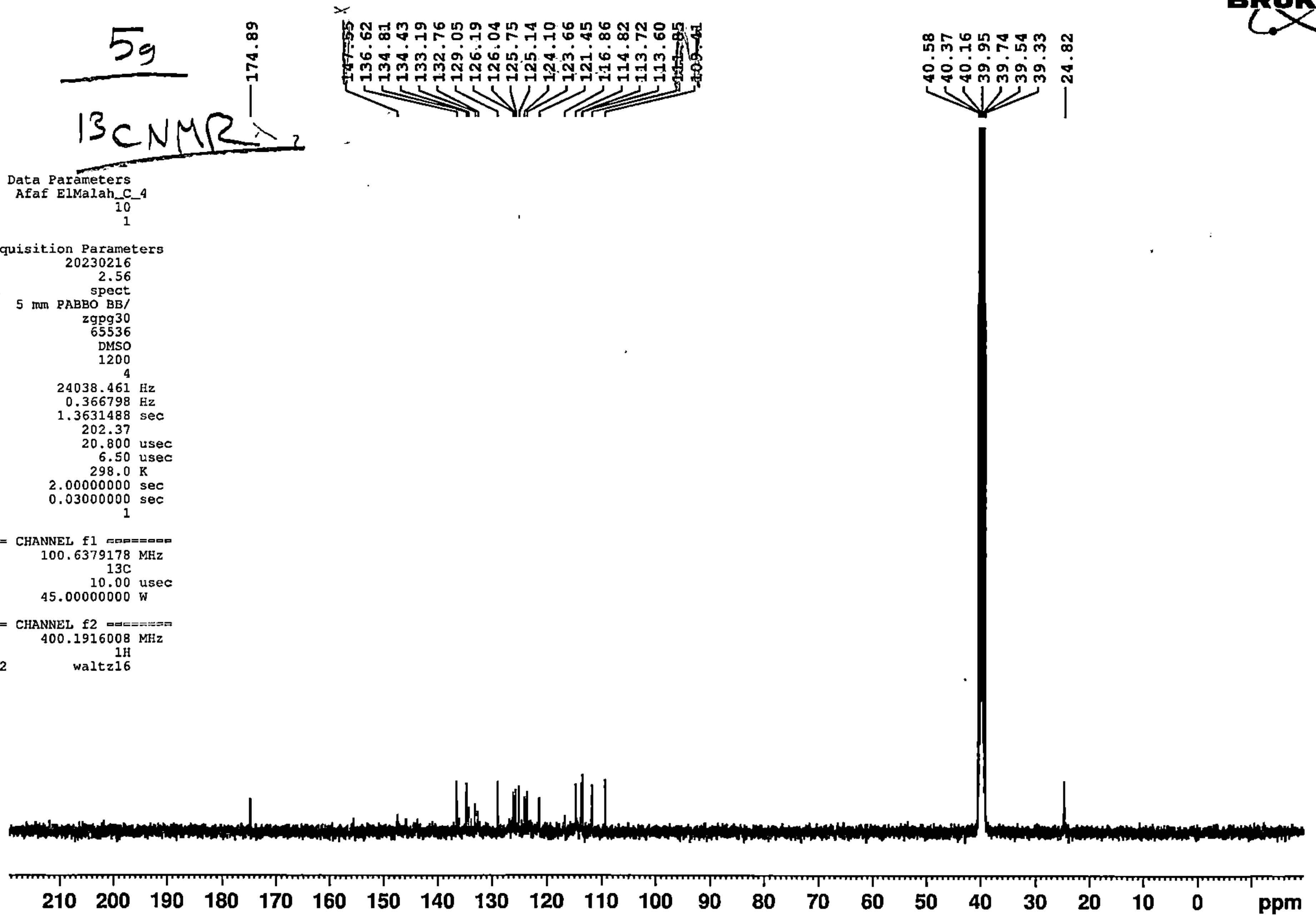


```
Current Data Parameters
NAME      Afaf ElMalah_C_4
EXPNO                   10
PROCNO                   1

F2 - Acquisition Parameters
Date_             20230216
Time                  2.56
INSTRUM              spect
PROBHD    5 mm PABBO BB/
PULPROG             zgpg30
TD                   65536
SOLVENT               DMSO
NS                    1200
DS                       4
SWH              24038.461 Hz
FIDRES            0.366798 Hz
AQ               1.3631488 sec
RG                  202.37
DW                  20.800 usec
DE                    6.50 usec
TE                   298.0 K
D1              2.00000000 sec
D11             0.03000000 sec
TD0                      1

======== CHANNEL f1 ========
SFO1         100.6379178 MHz
NUC1                   13C
P1                   10.00 usec
PLW1           45.00000000 W

======== CHANNEL f2 ========
SFO2         400.1916008 MHz
NUC2                    1H
CPDPRG[2           waltz16
```

Afaf ElMalah_H_2

5h

1HNMR

Microanalytical Unit - FOPCU - NMR laboratory
www.pharma.cu.edu.eg dir-mau.fopcu@pharma.cu.edu.eg

BRUKER

```
Current Data Parameters
NAME      Afaf ElMalah_H_2
EXPNO                   10
PROCNO                   1

F2 - Acquisition Parameters
Date_             20230216
Time                  0.31
INSTRUM              spect
PROBHD   5 mm PABBO BB/
PULPROG               zg30
TD                   65536
SOLVENT               DMSO
NS                      32
DS                       2
SWH             8012.820 Hz
FIDRES          0.122266 Hz
AQ             4.0894465 sec
RG                 106.37
DW                62.400 usec
DE                  6.50 usec
TE                 298.1 K
D1            1.00000000 sec
TD0                      1

======== CHANNEL f1 ========
SFO1         400.1924713 MHz
NUC1                    1H
P1                   15.00 usec
PLW1           10.39999962 W

F2 - Processing parameters
SI                   65536
SF            400.1900000 MHz
WDW                     EM
SSB         0
LB                    0.30 Hz
GB          0
PC                    1.00
```

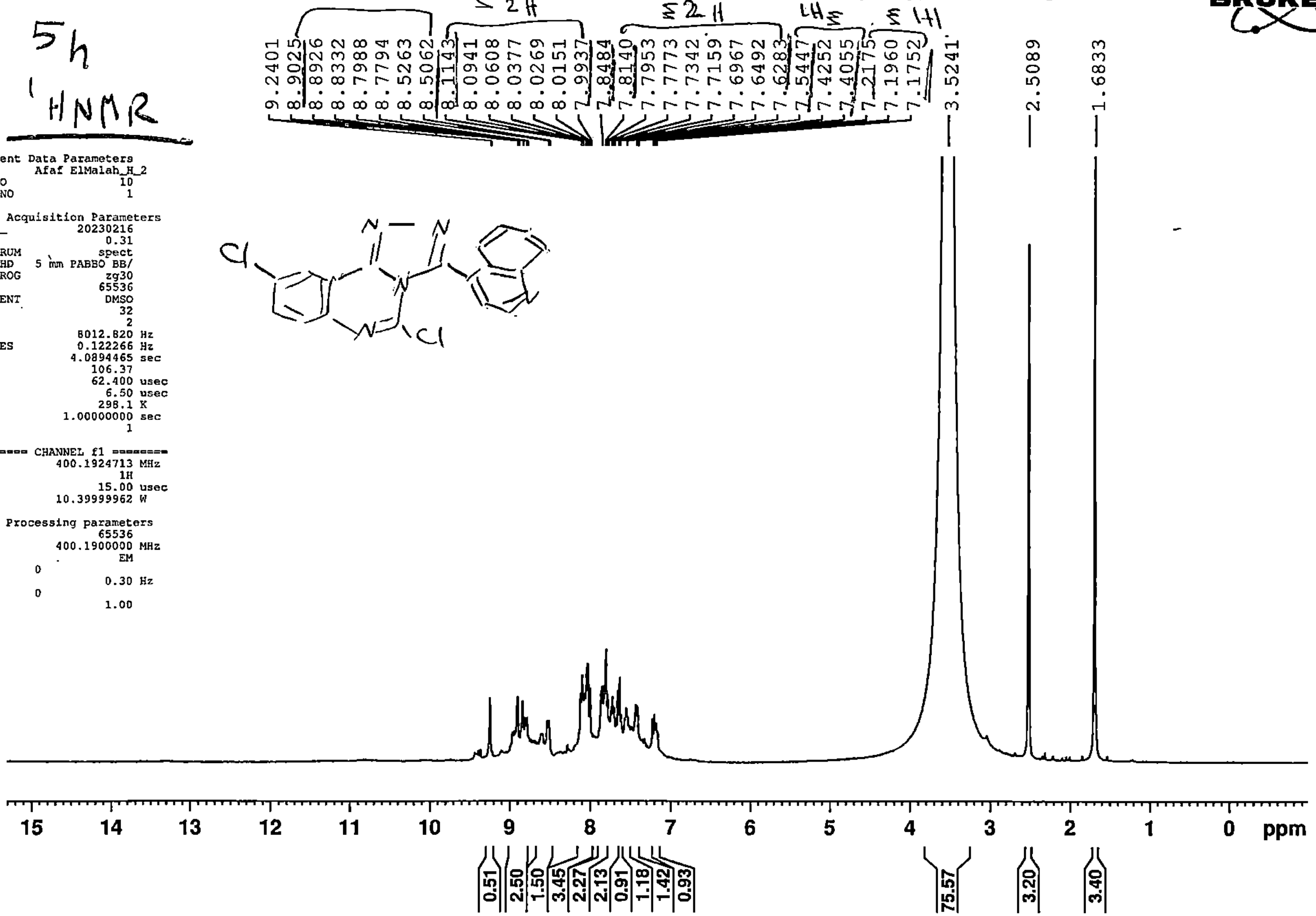

Afaf ElMalah_C_2

Microanalytical Unit - FOPCU - NMR laboratory
www.pharma.cu.edu.eg dir-mau.fopcu@pharma.cu.edu.eg

BRUKER

5h
13C NMR

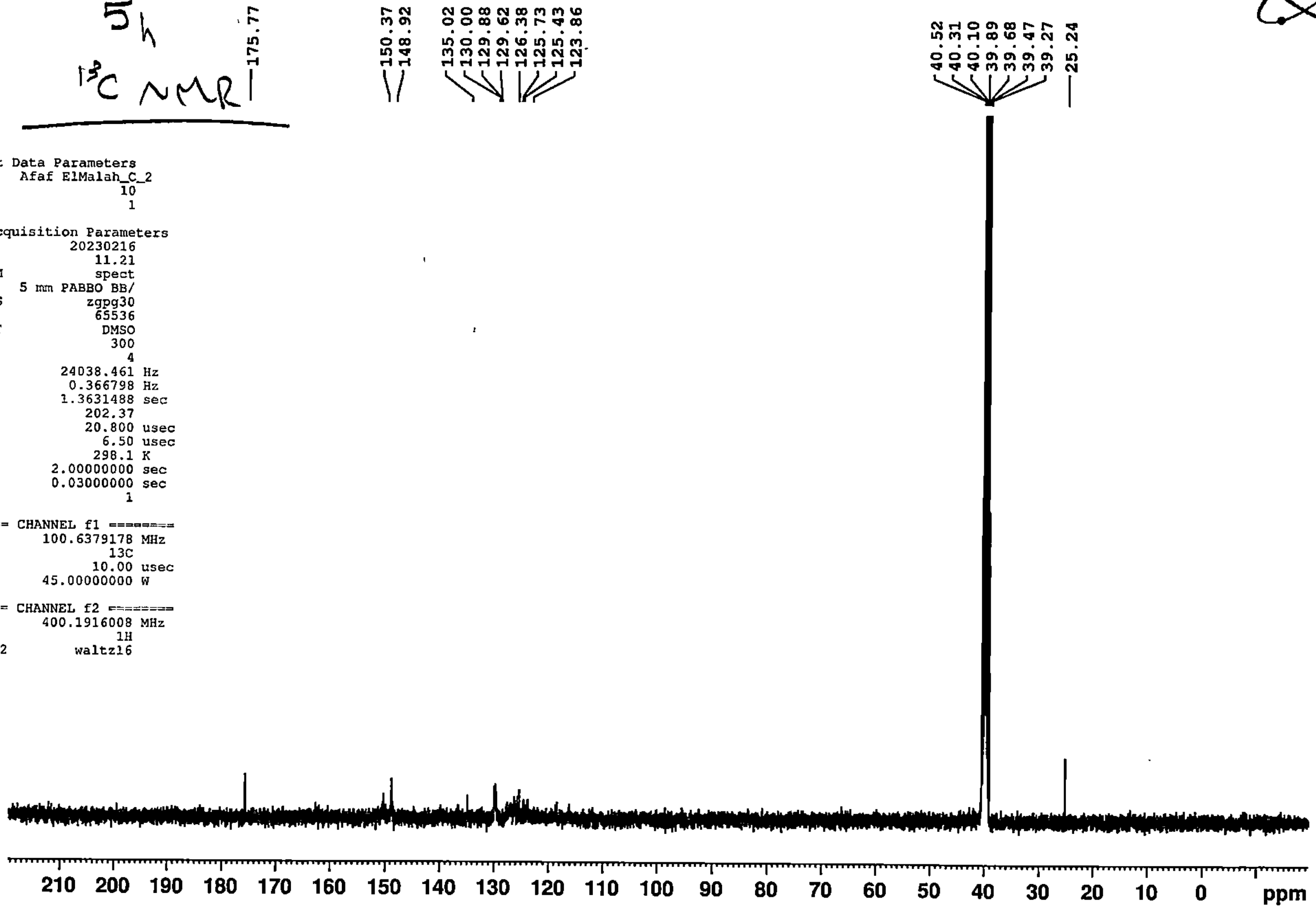


```
Current Data Parameters
NAME       Afaf ElMalah_C_2
EXPNO                    10
PROCNO                    1

F2 - Acquisition Parameters
Date_              20230216
Time                  11.21
INSTRUM               spect
PROBHD     5 mm PABBO BB/
PULPROG             zgpg30
TD                   65536
SOLVENT               DMSO
NS                      300
DS                        4
SWH              24038.461 Hz
FIDRES            0.366798 Hz
AQ               1.3631488 sec
RG                   202.37
DW                   20.800 usec
DE                     6.50 usec
TE                    298.1 K
D1               2.00000000 sec
D11              0.03000000 sec
TD0                       1

======== CHANNEL f1 ========
SFO1         100.6379178 MHz
NUC1                 13C
P1                 10.00 usec
PLW1         45.00000000 W

======== CHANNEL f2 ========
SFO2         400.1916008 MHz
NUC2                  1H
CPDPRG[2          waltz16
```
