# Supplementary material for: Design, synthesis, molecular docking, and molecular dynamic studies of novel quinazoline derivatives as phosphodiesterase 7 inhibitors
Source: Front Pharmacol. 2024 Apr 22;15:1389076. doi: 10.3389/fphar.2024.1389076 (PMC11070508; doi:10.3389/fphar.2024.1389076)

Researcher	: Dr.Afaf Ali email: Zeinab.mahmoud@pharma.cu.edu.eg Mob.01005840269
Assay	: PDE7A inh.assay
Samples	: 14 compounds
Cell lines	: —
Ref.	: —
Date	: 25-01-2023
Reader	: Tecan Spark Reader
Kit used	: —.
Solvent	: DMSO

Lab Report

ser	Compound		PDE7A	SD
	code	MW g/mol	IC50 ug/ml	±
1	4h s1	—	**0.627**	0.024
2	5h s2	—	**1.802**	0.068
3	4g s3	—	**0.15**	0.006
4	5g s4	—	**0.407**	0.015
5	4f s5	—	**0.343**	0.013
6	5f s6	—	**0.142**	0.005
7	4e s7	—	**1.966**	0.074
8	5e s8	—	**1.338**	0.051
9	4d s9	—	**0.249**	0.009
10	5d s10	—	**0.894**	0.034
11	4c s11	—	**0.478**	0.018
12	5c s12	—	**0.18**	0.007
13	4b s13	—	**0.114**	0.004
14	4a s15	—	**0.653**	0.025
***	BRL50481	—	**0.034**	0.002

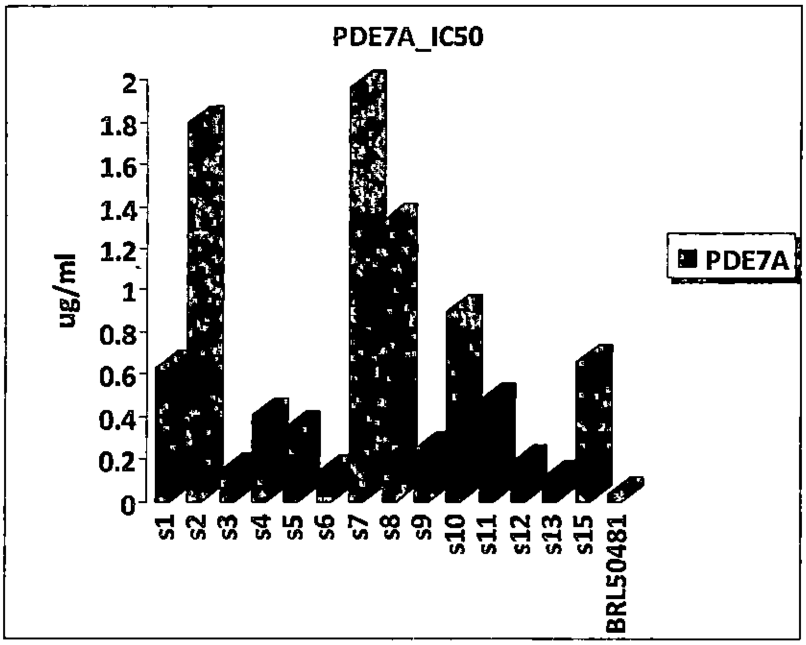

Detailed Results

PDE7A [redacted]

code	IC50	conc	log	%inh	T2	T1	ΔT	RFU2	RFU1	ΔRFU	slope	K.Activity
s1		100	2	90	30	0	30	9.61	0	9.61	3.33333	11.53201
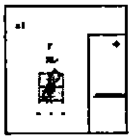		10	1	80	30	0	30	19.54	0	19.54	3.33333	23.44802
		1	0	52	30	0	30	47.82	0	47.82	3.33333	57.38406
		0.1	-1	35	30	0	30	65.19	0	65.19	3.33333	78.22808
		0.01	-2	13	30	0	30	87.42	0	87.42	3.33333	104.9041
EC				0	30	0	30	100	0	100	3.33333	120
	[redacted]			[redacted]			[redacted]		[redacted]		[redacted]	

code	IC50	conc	log	%inh	T2	T1	ΔT	RFU2	RFU1	ΔRFU	slope	K.Activity
s2		100	2	88	30	0	30	11.76	0	11.76	3.33333	14.11201
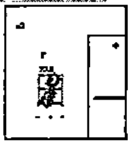		10	1	74	30	0	30	26.05	0	26.05	3.33333	31.26003
		1	0	35	30	0	30	64.53	0	64.53	3.33333	77.43608
		0.1	-1	19	30	0	30	81.33	0	81.33	3.33333	97.5961
		0.01	-2	5.4	30	0	30	94.58	0	94.58	3.33333	113.4961
EC				0	30	0	30	100	0	100	3.33333	120
	[redacted]			[redacted]			[redacted]		[redacted]		[redacted]	

code	IC50	conc	log	%inh	T2	T1	ΔT	RFU2	RFU1	ΔRFU	slope	K.Activity
s3		100	2	93	30	0	30	6.96	0	6.96	3.33333	8.352008
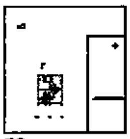		10	1	85	30	0	30	14.62	0	14.62	3.33333	17.54402
		1	0	66	30	0	30	33.82	0	33.82	3.33333	40.58404
		0.1	-1	43	30	0	30	57.16	0	57.16	3.33333	68.59207
		0.01	-2	31	30	0	30	68.91	0	68.91	3.33333	82.69208
EC				0	30	0	30	100	0	100	3.33333	120
	[redacted]			[redacted]			[redacted]		[redacted]		[redacted]	

code	IC50	conc	log	%inh	T2	T1	ΔT	RFU2	RFU1	ΔRFU	slope	K.Activity
s4		100	2	93	30	0	30	7.29	0	7.29	3.33333	8.748009
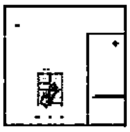		10	1	79	30	0	30	21.38	0	21.38	3.33333	25.65603
		1	0	61	30	0	30	38.59	0	38.59	3.33333	46.30805
		0.1	-1	38	30	0	30	62.02	0	62.02	3.33333	74.42407
		0.01	-2	17	30	0	30	83.16	0	83.16	3.33333	99.7921
EC				0	30	0	30	100	0	100	3.33333	120
	[redacted]			[redacted]			[redacted]		[redacted]		[redacted]	

code	IC50	conc	log	%inh	T2	T1	ΔT	RFU2	RFU1	ΔRFU	slope	K.Activity
s5		100	2	93	30	0	30	7.47	0	7.47	3.33333	8.964009
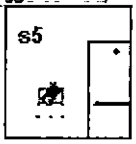		10	1	81	30	0	30	19.33	0	19.33	3.33333	23.19602
		1	0	59	30	0	30	41.22	0	41.22	3.33333	49.46405
		0.1	-1	35	30	0	30	64.58	0	64.58	3.33333	77.49608
		0.01	-2	22	30	0	30	78.39	0	78.39	3.33333	94.06809
EC				0	30	0	30	100	0	100	3.33333	120
	[redacted]			[redacted]			[redacted]		[redacted]		[redacted]	

code	IC50	conc	log	%inh	T2	T1	ΔT	RFU2	RFU1	ΔRFU	slope	K.Activity
s6		100	2	93	30	0	30	6.88	0	6.88	3.33333	8.256008
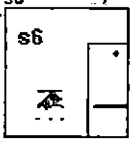		10	1	88	30	0	30	15.86	0	15.86	4.33333	14.64001
		1	0	66	30	0	30	34.02	0	34.02	3.33333	40.82404
		0.1	-1	44	30	0	30	55.79	0	55.79	3.33333	66.94807
		0.01	-2	32	30	0	30	68.43	0	68.43	3.33333	82.11608
EC				0	30	0	30	100	0	100	3.33333	120
	[redacted]			[redacted]			[redacted]		[redacted]		[redacted]	

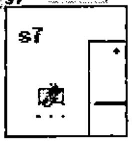

code	IC50	conc	log	%inh	T2	T1	ΔT	RFU2	RFU1	ΔRFU	slope	K.Activity
s7		100	2	89	30	0	30	11.06	0	11.06	3.33333	13.27201
		10	1	70	30	0	30	29.51	0	29.51	3.33333	35.41204
		1	0	35	30	0	30	65.45	0	65.45	3.33333	78.54008
		0.1	-1	18	30	0	30	82.42	0	82.42	3.33333	98.9041
		0.01	-2	6.5	30	0	30	93.53	0	93.53	3.33333	112.2361
EC				0	30	0	30	100	0	100	3.33333	120
	[redacted]			[redacted]			[redacted]		[redacted]		[redacted]	

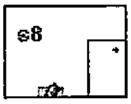

code	IC50	conc	log	%inh	T2	T1	ΔT	RFU2	RFU1	ΔRFU	slope	K.Activity
s8		100	2	91	30	0	30	8.65	0	8.65	3.33333	10.38001
		10	1	75	30	0	30	24.76	0	24.76	3.33333	29.71203
		1	0	40	30	0	30	59.66	0	59.66	3.33333	71.59207
		0.1	-1	19	30	0	30	81.03	0	81.03	3.33333	97.2361
		0.01	-2	10	30	0	30	89.72	0	89.72	3.33333	107.6641
EC				0	30	0	30	100	0	100	3.33333	120
	[redacted]			[redacted]			[redacted]		[redacted]		[redacted]	

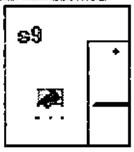

code	IC50	conc	log	%inh	T2	T1	ΔT	RFU2	RFU1	ΔRFU	slope	K.Activity
s9		100	2	94	30	0	30	6.42	0	6.42	3.33333	7.704008
		10	1	84	30	0	30	16.08	0	16.08	3.33333	19.29602
		1	0	64	30	0	30	35.79	0	35.79	3.33333	42.94804
		0.1	-1	38	30	0	30	62.03	0	62.03	3.33333	74.43607
		0.01	-2	25	30	0	30	74.59	0	74.59	3.33333	89.50809
EC				0	30	0	30	100	0	100	3.33333	120
	[redacted]			[redacted]			[redacted]		[redacted]		[redacted]	

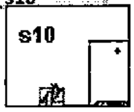

code	IC50	conc	log	%inh	T2	T1	ΔT	RFU2	RFU1	ΔRFU	slope	K.Activity
s10		100	2	91	30	0	30	8.81	0	8.81	3.33333	10.57201
		10	1	77	30	0	30	23.27	0	23.27	3.33333	27.92403
		1	0	46	30	0	30	54.16	0	54.16	3.33333	64.99206
		0.1	-1	29	30	0	30	71.35	0	71.35	3.33333	85.62009
		0.01	-2	13	30	0	30	87.42	0	87.42	3.33333	104.9041
EC				0	30	0	30	100	0	100	3.33333	120
	[redacted]			[redacted]			[redacted]		[redacted]		[redacted]	

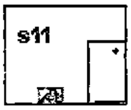

code	IC50	conc	log	%inh	T2	T1	ΔT	RFU2	RFU1	ΔRFU	slope	K.Activity
s11		100	2	93	30	0	30	6.96	0	6.96	3.33333	8.352008
		10	1	84	30	0	30	15.85	0	15.85	3.33333	19.02002
		1	0	57	30	0	30	42.89	0	42.89	3.33333	51.46805
		0.1	-1	33	30	0	30	66.71	0	66.71	3.33333	80.05208
		0.01	-2	15	30	0	30	84.59	0	84.59	3.33333	101.5081
EC				0	30	0	30	100	0	100	3.33333	120
	[redacted]			[redacted]			[redacted]		[redacted]		[redacted]	

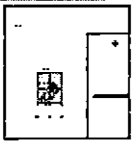

code	IC50	conc	log	%inh	T2	T1	ΔT	RFU2	RFU1	ΔRFU	slope	K.Activity
s12		100	2	94	30	0	30	5.75	0	5.75	3.33333	6.900007
		10	1	87	30	0	30	13.02	0	13.02	3.33333	15.62402
		1	0	62	30	0	30	37.82	0	37.82	3.33333	45.38405
		0.1	-1	45	30	0	30	54.89	0	54.89	3.33333	65.86807
		0.01	-2	27	30	0	30	72.91	0	72.91	3.33333	87.49209
EC				0	30	0	30	100	0	100	3.33333	120
	[redacted]			[redacted]			[redacted]		[redacted]		[redacted]	

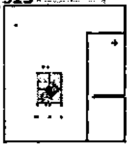

code	IC50	conc	log	%inh	T2	T1	ΔT	RFU2	RFU1	ΔRFU	slope	K.Activity
s13		100	2	94	30	0	30	5.91	0	5.91	3.33333	7.092007
		10	1	89	30	0	30	11.46	0	11.46	3.33333	13.75201
		1	0	71	30	0	30	28.52	0	28.52	3.33333	34.22403

		0.1	-1	48	30	0	30	51.55	0	51.55	3.33333	61.86006
		0.01	-2	28	30	0	30	71.62	0	71.62	3.33333	85.94409
EC				0	30	0	30	100	0	100	3.33333	120
		[redacted]		[redacted]	[redacted]		[redacted]		[redacted]		[redacted]	

code	IC50	conc	log	%inh	T2	T1	ΔT	RFU2	RFU1	ΔRFU	slope	K.Activity
s15		100	2	91	30	0	30	8.52	0	8.52	3.33333	10.22401
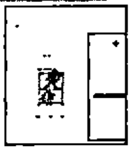		10	1	80	30	0	30	19.62	0	19.62	3.33333	23.54402
		1	0	54	30	0	30	45.99	0	45.99	3.33333	55.18806
		0.1	-1	27	30	0	30	72.63	0	72.63	3.33333	87.15609
		0.01	-2	16	30	0	30	84.31	0	84.31	3.33333	101.1721
EC				0	30	0	30	100	0	100	3.33333	120
		[redacted]		[redacted]	[redacted]		[redacted]		[redacted]		[redacted]	

code	IC50	conc	log	%inh	T2	T1	ΔT	RFU2	RFU1	ΔRFU	slope	K.Activity
BRL50481		100	2	95	30	0	30	5.29	0	5.29	3.33333	6.348006
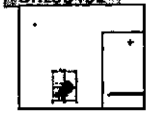		10	1	89	30	0	30	11.36	0	11.36	3.33333	13.63201
		1	0	79	30	0	30	21.44	0	21.44	3.33333	25.72803
		0.1	-1	57	30	0	30	43.02	0	43.02	3.33333	51.62405
		0.01	-2	38	30	0	30	62.26	0	62.26	3.33333	74.71207
EC				0	30	0	30	100	0	100	3.33333	120
		[redacted]		[redacted]	[redacted]		[redacted]		[redacted]	[redacted]	[redacted]	

**s1**	y = 20.127x + 54.084
**s2**	y = 22.092x + 44.35
**s3**	y = 16.644x + 63.706
**s4**	y = 19.238x + 57.512
**s5**	y = 18.709x + 57.802
**s6**	y = 16.669x + 64.536
**s7**	y = 21.785x + 43.606
**s8**	y = 21.841x + 47.236
**s9**	y = 18.229x + 61.018
**s10**	y = 20.53x + 50.998
**s11**	y = 20.612x + 56.6
**s12**	y = 17.619x + 63.122
**s13**	y = 17.151x + 66.188
**s15**	y = 20.459x + 53.786

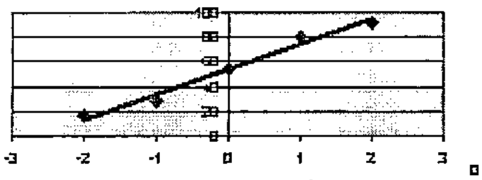

Researcher : Dr.Zeinab Mohamed email: Zeinab.mahmoud@pharma.cu.edu.eg
Mob. : 01005840269
Assay : **PDE7A** inh.assay
Samples : 12 compounds
Cell lines : ---
Ref. : ---
Date : 25-01-2023
Reader : Tecan Spark Reader
Kit used : ---.

Lab Report

ser	Compound		PDE7A	SD
	code	MW g/mol	IC50 ug/ml	±
1	4b	---	0.559	0.025
2	5c	---	0.227	0.01
3	7c	---	0.076	0.003
4	5b	---	0.753	0.033
5	7a	---	0.389	0.017
6	4a	---	1.121	0.049
7	7b	---	0.563	0.025
8	4c	---	1.35	0.059
9	5a — 6b	---	0.251	0.011
10	5a	---	1.083	0.048
11	5b — 6a	---	0.32	0.014
12	6c	---	0.062	0.003
***	Theophylline	---	0.539	0.024

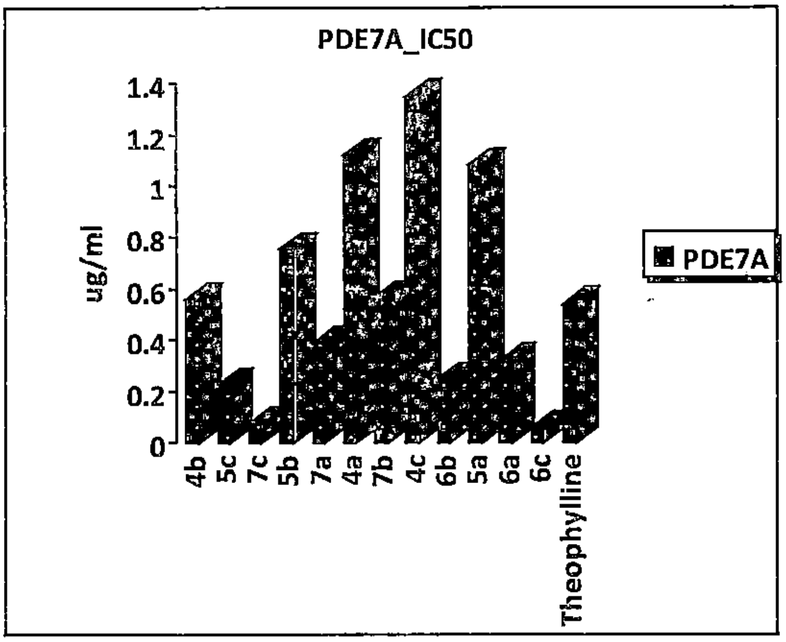

Detailed Results

PDE7A [redacted]

code	IC50	conc	log	%inh	T2	T1	ΔT	RFU2	RFU1	ΔRFU	slope	K.Activity
4b		100	2	89	30	0	30	11.4	0	11.4	3.33333	13.68001
		10	1	80	30	0	30	19.72	0	19.72	3.33333	23.66402
		1	0	56	30	0	30	43.64	0	43.64	3.33333	52.36805
		0.1	-1	33	30	0	30	66.82	0	66.82	3.33333	80.18408
		0.01	-2	16	30	0	30	84.13	0	84.13	3.33333	100.9561
EC				0	30	0	30	100	0	100	3.33333	120
		[redacted]		[redacted]			[redacted]		[redacted]		[redacted]	

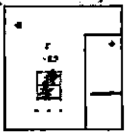

code	IC50	conc	log	%inh	T2	T1	ΔT	RFU2	RFU1	ΔRFU	slope	K.Activity
5c		100	2	93	30	0	30	6.61	0	6.61	3.33333	7.932008
		10	1	87	30	0	30	13.08	0	13.08	3.33333	15.69602
		1	0	60	30	0	30	39.55	0	39.55	3.33333	47.46005
		0.1	-1	42	30	0	30	58.44	0	58.44	3.33333	70.12807
		0.01	-2	26	30	0	30	74.16	0	74.16	3.33333	88.99209
EC				0	30	0	30	100	0	100	3.33333	120
		[redacted]		[redacted]			[redacted]		[redacted]		[redacted]	

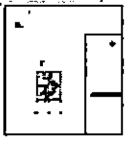

code	IC50	conc	log	%inh	T2	T1	ΔT	RFU2	RFU1	ΔRFU	slope	K.Activity
7c		100	2	94	30	0	30	6.03	0	6.03	3.33333	7.236007
		10	1	88	30	0	30	11.84	0	11.84	3.33333	14.20801
		1	0	71	30	0	30	28.95	0	28.95	3.33333	34.74003
		0.1	-1	49	30	0	30	51.03	0	51.03	3.33333	61.23606
		0.01	-2	35	30	0	30	64.72	0	64.72	3.33333	77.66408
EC				0	30	0	30	100	0	100	3.33333	120
		[redacted]		[redacted]			[redacted]		[redacted]		[redacted]	

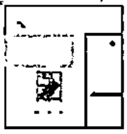

code	IC50	conc	log	%inh	T2	T1	ΔT	RFU2	RFU1	ΔRFU	slope	K.Activity
5b		100	2	89	30	0	30	11.28	0	11.28	3.33333	13.53601
		10	1	80	30	0	30	19.58	0	19.58	3.33333	23.49602
		1	0	51	30	0	30	49.33	0	49.33	3.33333	59.19606
		0.1	-1	28	30	0	30	72.03	0	72.03	3.33333	86.43609
		0.01	-2	15	30	0	30	85.44	0	85.44	3.33333	102.5281
EC				0	30	0	30	100	0	100	3.33333	120
		[redacted]		[redacted]			[redacted]		[redacted]		[redacted]	

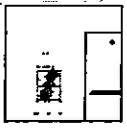

code	IC50	conc	log	%inh	T2	T1	ΔT	RFU2	RFU1	ΔRFU	slope	K.Activity
7a		100	2	91	30	0	30	9.42	0	9.42	3.33333	11.30401
7a		10	1	84	30	0	30	15.67	0	15.67	3.33333	18.80402
		1	0	58	30	0	30	41.55	0	41.55	3.33333	49.86005
		0.1	-1	37	30	0	30	62.79	0	62.79	3.33333	75.34808
		0.01	-2	19	30	0	30	81.42	0	81.42	3.33333	97.7041
EC				0	30	0	30	100	0	100	3.33333	120
		[redacted]		[redacted]			[redacted]		[redacted]		[redacted]	

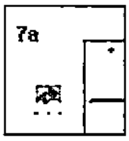

code	IC50	conc	log	%inh	T2	T1	ΔT	RFU2	RFU1	ΔRFU	slope	K.Activity

code	IC50	conc	log	%inh	T2	T1	ΔT	RFU2	RFU1	ΔRFU	slope	K.Activity
4a 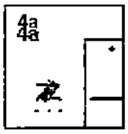		100	2	88	30	0	30	11.86	0	11.86	3.33333	14.23201
		10	1	78	30	0	30	28.49	0	28.49	4.33333	26.29848
		1	0	42	30	0	30	57.63	0	57.63	3.33333	69.15607
		0.1	-1	25	30	0	30	74.82	0	74.82	3.33333	89.78409
		0.01	-2	11	30	0	30	88.92	0	88.92	3.33333	106.7041
EC				0	30	0	30	100	0	100	3.33333	120
		[redacted]		[redacted]			[redacted]		[redacted]		[redacted]	

code	IC50	conc	log	%inh	T2	T1	ΔT	RFU2	RFU1	ΔRFU	slope	K.Activity
7b		100	2	92	30	0	30	8.32	0	8.32	3.33333	9.98401
7b 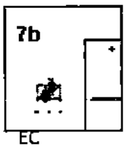		10	1	79	30	0	30	21.03	0	21.03	3.33333	25.23603
		1	0	53	30	0	30	47.28	0	47.28	3.33333	56.73606
		0.1	-1	33	30	0	30	67.03	0	67.03	3.33333	80.43608
		0.01	-2	18	30	0	30	82.19	0	82.19	3.33333	98.6281
EC				0	30	0	30	100	0	100	3.33333	120
		[redacted]		[redacted]			[redacted]		[redacted]		[redacted]	

code	IC50	conc	log	%inh	T2	T1	ΔT	RFU2	RFU1	ΔRFU	slope	K.Activity
4c		100	2	90	30	0	30	9.94	0	9.94	3.33333	11.92801
4c 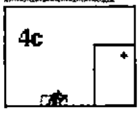		10	1	76	30	0	30	23.57	0	23.57	3.33333	28.28403
		1	0	43	30	0	30	57.44	0	57.44	3.33333	68.92807
		0.1	-1	19	30	0	30	81.02	0	81.02	3.33333	97.2241
		0.01	-2	7.4	30	0	30	92.55	0	92.55	3.33333	111.0601
EC				0	30	0	30	100	0	100	3.33333	120
		[redacted]		[redacted]			[redacted]		[redacted]		[redacted]	

code	IC50	conc	log	%inh	T2	T1	ΔT	RFU2	RFU1	ΔRFU	slope	K.Activity
6b		100	2	93	30	0.	30	7.29	0	7.29	3.33333	8.748009
6b 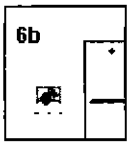		10	1	85	30	0	30	15.33	0	15.33	3.33333	18.39602
		1	0	70	30	0	30	29.94	0	29.94	3.33333	35.92804
		0.1	-1	38	30	0	30	62.42	0	62.42	3.33333	74.90407
		0.01	-2	22	30	0	30	78.28	0	78.28	3.33333	93.93609
EC				0	30	0	30	100	0	100	3.33333	120
		[redacted]		[redacted]			[redacted]		[redacted]		[redacted]	

code	IC50	conc	log	%inh	T2	T1	ΔT	RFU2	RFU1	ΔRFU	slope	K.Activity
5a		100	2	90	30	0	30	9.91	0	9.91	3.33333	11.89201
5a 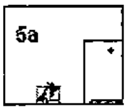		10	1	70	30	0	30	29.56	0	29.56	3.33333	35.47204
		1	0	47	30	0	30	53.28	0	53.28	3.33333	63.93606
		0.1	-1	28	30	0	30	72.09	0	72.09	3.33333	86.50809
		0.01	-2	11	30	0	30	88.64	0	88.64	3.33333	106.3681
EC				0	30	0	30	100	0	100	3.33333	120
		[redacted]		[redacted]			[redacted]		[redacted]		[redacted]	

code	IC50	conc	log	%inh	T2	T1	ΔT	RFU2	RFU1	ΔRFU	slope	K.Activity
6a		100	2	93	30	0	30	6.59	0	6.59	3.33333	7.908008
6a 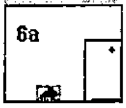		10	1	83	30	0	30	17.42	0	17.42	3.33333	20.90402
		1	0	62	30	0	30	38.11	0	38.11	3.33333	45.73205
		0.1	-1	35	30	0	30	65.02	0	65.02	3.33333	78.02408
		0.01	-2	23	30	0	30	76.51	0	76.51	3.33333	91.81209
EC				0	30	0	30	100	0	100	3.33333	120
		[redacted]		[redacted]			[redacted]		[redacted]		[redacted]	

code	IC50	conc	log	%inh	T2	T1	ΔT	RFU2	RFU1	ΔRFU	slope	K.Activity
6c		100	2	94	30	0	30	6.19	0	6.19	3.33333	7.428007
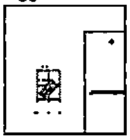		10	1	88	30	0	30	12.08	0	12.08	3.33333	14.49601
		1	0	73	30	0	30	27.42	0	27.42	3.33333	32.90403
		0.1	-1	54	30	0	30	45.82	0	45.82	3.33333	54.98405
		0.01	-2	34	30	0	30	66.03	0	66.03	3.33333	79.23608

EC 0 30 0 30 100 0 100 3.33333 120

[redacted] [redacted] [redacted] [redacted] [redacted]

code	IC50	conc	log	%inh	T2	T1	ΔT	RFU2	RFU1	ΔRFU	slope	K.Activity	EC
		100	2	92	30	0	30	8.28	0	8.28	3.33333	9.93601	120
Theophylline													
		10	1	75	30	0	30	24.54	0	24.54	3.33333	29.44803	120
		1	0	54	30	0	30	45.71	0	45.71	3.33333	54.85205	120
		0.1	-1	35	30	0	30	64.65	0	64.65	3.33333	77.58008	120
		0.01	-2	18	30	0	30	81.71	0	81.71	3.33333	98.0521	120
EC				0	30	0	30	100	0	100	3.33333	120	120
		[redacted]		[redacted]			[redacted]		[redacted]		[redacted]		

**4b**	y = 19.256x + 54.858
**5c**	y = 18.046x + 61.632
**7c**	y = 15.657x + 67.486
**5b**	y = 20.077x + 52.468
**7a**	y = 19.112x + 57.83
**4a**	y = 20.702x + 48.971
**7b**	y = 19.374x + 54.83
**4c**	y = 22.267x + 47.096
**6b**	y = 18.907x + 61.348
**5a**	y = 19.999x + 49.304
**6a**	y = 18.744x + 59.27
**6c**	y = 15.342x + 68.492
**Theophylline**	y = 18.697x + 55.022

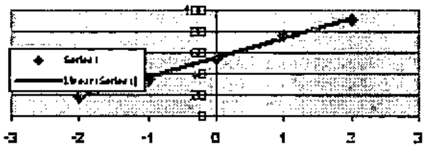

Supplement: Supplementary file 2 [file DataSheet1.PDF]
